# Comparison of out-of-hospital cardiac arrests during the COVID-19 pandemic with those before the pandemic: an updated systematic review and meta-analysis

**DOI:** 10.3389/fpubh.2023.1180511

**Published:** 2023-05-04

**Authors:** Jae Hwan Kim, Chiwon Ahn, Yeonkyung Park, Moonho Won

**Affiliations:** ^1^Department of Emergency Medicine, College of Medicine, Chung-Ang University, Seoul, Republic of Korea; ^2^Division of Pulmonary and Critical Care Medicine, Department of Internal Medicine, Veterans Health Service Medical Center, Seoul, Republic of Korea

**Keywords:** out-of-hospital cardiac arrest, COVID-19, meta-analysis, survival, epidemioloy

## Abstract

The coronavirus disease of 2019 (COVID-19) pandemic, directly and indirectly, affected the emergency medical care system and resulted in worse out-of-hospital cardiac arrest (OHCA) outcomes and epidemiological features compared with those before the pandemic. This review compares the regional and temporal features of OHCA prognosis and epidemiological characteristics. Various databases were searched to compare the OHCA outcomes and epidemiological characteristics during the COVID-19 pandemic with before the pandemic. During the COVID-19 pandemic, survival and favorable neurological outcome rates were significantly lower than before. Survival to hospitalization, return of spontaneous circulation, endotracheal intubation, and use of an automated external defibrillator (AED) decreased significantly, whereas the use of a supraglottic airway device, the incidence of cardiac arrest at home, and response time of emergency medical service (EMS) increased significantly. Bystander CPR, unwitnessed cardiac arrest, EMS transfer time, use of mechanical CPR, and in-hospital target temperature management did not differ significantly. A subgroup analysis of the studies that included only the first wave with those that included the subsequent waves revealed the overall outcomes in which the epidemiological features of OHCA exhibited similar patterns. No significant regional differences between the OHCA survival rates in Asia before and during the pandemic were observed, although other variables varied by region. The COVID-19 pandemic altered the epidemiologic characteristics, survival rates, and neurological prognosis of OHCA patients.

**Review registration**: PROSPERO (CRD42022339435).

## Introduction

Worldwide out-of-hospital cardiac arrest (OHCA) has a reported incidence of 55–88 per 100,000, and is an important national health problem that has a high mortality rate if not treated properly (1–6). The International Liaison Committee on Resuscitation reported that emergency medical service (EMS)-treated OHCA and bystander CPR increased steadily (7). As OHCA onset is difficult to predict and occurs in places other than hospitals, various social and medical components are required in the treatment process (8). In the past 40 years, the survival rate of OHCA patients worldwide has been improving, and the factors that influenced this improved survival include rapid recognition of patients by bystanders, bystander CPR, rapid EMS response, and the use of AEDs (8–10). Moreover, post-OHCA survival may be affected by the characteristics of the emergency medical care system in regions and countries where the cardiac arrest has occurred (1, 3).

Coronavirus disease of 2019 (COVID-19) first emerged in December 2019 and spread globally; on March 11, 2020, a pandemic was declared by the World Health Organization (WHO) (11). By October 2022, approximately 600 million people worldwide had been diagnosed with COVID-19, which resulted in approximately 6.5 million deaths (12). Paramedics were equipped with personal protective equipment and took additional steps to prepare ambulances for dispatch to access emergency patients at risk of infection, which contributed to delays in dispatch (13). Moreover, hospitals faced difficulties in securing isolation spaces and beds for critical care, which led to patient-capacity restrictions that affected the medical care for not only patients with fevers but also non-COVID-19 emergency patients (14–17). In the early days of the pandemic, the healthcare system was unprepared to face the pandemic, and the majority of medical resources were concentrated on the care of COVID-19 patients, which resulted in a collateral impact on the outcomes of various other conditions. Hospitalizations and mortality rates increased for patients with acute myocardial infarction and ischemic stroke, complication rates in patients with acute peritonitis increased, and time to surgery was delayed (14–16). The incidence and mortality rates of OHCA, which has a very high fatality rate when immediate appropriate treatment is not given, have also increased (18, 19). In addition, the desire to “social distance” may have led to a decrease in EMS or hospital utilization in serious cases.

Several previous observational studies and meta-analyses have reported the incidence rate, epidemiological characteristics, and prognosis of OHCA patients during the pandemic compared with the pre-COVID-19 pandemic period (20–23). Shockable rhythms, automatic external defibrillator application use, and endotracheal intubation use were reported to have decreased, whereas EMS reaction time, arrest-at-home frequency, and use of supraglottic airways were reported to have increased (20–23). Treatment-induced return of spontaneous circulation (ROSC), survival to admission, survival to discharge, 30-day survival, and favorable neurological outcomes all had poor results during the pandemic (20–23). The pandemic had a significant impact on the survival and neurological prognosis of OHCA patients. However, as the COVID-19 pandemic continues, meta-analyses of the entire pandemic period that do not evaluate the effects of each period and region can potentially distort the results.

The medical system experienced a decrease in the care of various other diseases due to the focus on COVID-19 treatments at the beginning of the pandemic. The development of therapeutics and vaccines, the reduction in deadly COVID-19 subvariants, the change in the dominant variant, and the improved awareness of the general population and medical staff, who initially suffered panic and fear, have led to improvements to the emergency medical care system (24, 25).

As the emergency medical care system improved over time and adapted to different regional changes, a targeted, stratified, and refined meta-analysis is necessary to address the knowledge gap that exists despite the existing meta-analyses. The purpose of this review is to examine the most recent trends of change in epidemiological factors, prehospital factors, and outcomes for OHCA affected by the COVID-19 pandemic.

## Methods

### Reporting guidelines and protocol registration

This systematic review and meta-analysis were performed in accordance with the Preferred Reporting Items for Systematic Reviews and Meta-analysis (PRISMA) guidelines (26). The review protocol was registered in The International Prospective Register of Systematic Reviews (PROSPERO; CRD42022339435).

### Eligibility criteria

The participants were adult OHCA patients (age ≥18 years) from before and after the start of the COVID-19 pandemic period. The survival rate was the primary outcome, and the secondary endpoint was comprised of the factors that are associated with each stage of the survival chain and good neurological prognoses (Supplementary Table S1).

### Search strategy

In October 2022, PubMed, EMBASE, Cochrane Library, bioRxiv, and medRxiv databases were searched to identify relevant studies. In addition, the reference lists of previous meta-analyses were reviewed to identify and include any missing studies. Analyzable studies were manually searched on Google Scholar.

An extensive search based on the two keywords, “COVID-19 pandemic” and “out-of-hospital cardiac arrest,” as well as the related MeSH terms and Embase subject headings, was conducted. The search strategies that were used for each database search are described in Supplementary Table S2.

### Study selection

Duplicated references were excluded using the bibliographic management program (Endnote 20; Clarivate Analytics, Philadelphia, PA, United States) based on the title, author, and year of publication. Two researchers independently checked the titles and abstracts of the articles to implement the primary exclusion. The full-text articles were subsequently extracted and reviewed, and a second exclusion was carried out. Through the consensus of two reviewers, the studies were selected for inclusion in the final analysis. If there was a disagreement about the studies to be included, the final decision was made after seeking the opinion of an expert in the field.

The exclusion criteria were as follows: the absence of comparative data between the time of the COVID-19 pandemic and the preceding period; the primary result was missing; studies that used redundant data; and review articles, case reports, editorials, commentaries, meta-analyses, animal studies, and molecular biology studies.

Finally, retrospective observational studies in OHCA patients aged 18 years and older that presented variables associated with prognosis and the chain of survival after cardiac arrest and compared the time of the COVID-19 pandemic with the preceding period were included in this systematic review and meta-analysis.

### Data extraction

Data extraction was conducted independently after a sufficient discussion by two researchers; if there was disagreement, the decision was made by a majority vote that incorporated the opinion of a third expert. The information provided in the studies was extracted as objectively and reliably as possible and included the following details: study information (randomly assigned study number, author name, year of publication, country where the study was conducted), research method (study design), study subjects (total number of participants, study period, age, sex), survival discharge rate, neurological prognosis, 30-day survival rate, rate of survival to hospital admission, target body temperature treatment, spontaneous circulation recovery rate, tracheal intubation, use of glottal gastric airway, CPR performed using mechanical CPR, automatic external defibrillator use, witness CPR, unwitnessed cardiac arrest, shock required rhythm, cardiac arrest at home, EMS response time, and EMS transport time. The EMS response time was defined as the time it took for the paramedic to make contact with the patient after the cardiac arrest was first reported to the emergency agency. The EMS transport time was defined as the time it took from the cardiac arrest site to the arrival at the medical institution.

Data on the EMS response time and EMS transport time were collected as the mean and standard deviation as a continuity variable, and in studies where the data were presented as the median and quartiles, these were converted to the mean and standard deviation (27).

### Quality assessment in individual studies

A quality assessment of the included studies was performed to determine whether the studies were conducted appropriately for the stated purpose. The studies included in this meta-analysis were non-randomized clinical trials (NRCT) and were evaluated using the Risk of Bias in Non-randomized Studies of Interventions (ROBINS-I) (28). ROBINS-I consists of seven areas: (1) bias due to confounding, (2) bias in study subject selection, (3) bias in the classification of interventions during interventions, assessed to identify pre-intervention confounding, (4) bias due to the deviation from the intended intervention, (5) bias due to missing values, (6) bias in measuring intervention outcomes, and (7) bias in the selection of reported study outcomes to assess post-intervention bias.

### Statistical analysis

This meta-analysis investigated the epidemiological characteristics and outcomes of OHCA during the COVID-19 pandemic compared with that of before the pandemic. Individual and pooled statistics were calculated as the odds ratio (OR) and mean difference with a 95% confidence interval (CI). The random-effects model was used to determine the pooled outcome from the included studies, based on the diversity of the medical system according to the nation, region, and study period. We estimated the proportion of inter-study inconsistency using the *I*^2^ statistic to assess the heterogeneity. We considered *I*^2^ values of 25%, 50%, and 75% as low, moderate, and high heterogeneity, respectively (29).

Subgroup analyses were conducted according to the study period (with the first wave-only versus with sequential wave periods with and without the first wave) and regional (Asia, Europe, and the United States and Australia). Emergency care systems had different tendencies in the first wave compared with the subsequent waves of the COVID-10 pandemic. Previous studies have shown that there were lower severity and mortality rates in COVID-19 patients after the first wave due to changes in the system (30–33). In the subgroup analysis in this study, studies that analyzed only the first wave and those that included the subsequent wave periods were separately analyzed. However, in the group of studies including subsequent wave periods, data from the first wave were included. The first wave was from March to June 2020, according to the WHO’s COVID-19 incidence graph. In addition, there were differences in the prevalence of the COVID-19 pandemic by country and region due to a combination of healthcare systems and social and cultural factors in response to the COVID-19 pandemic. Stratified analyses were performed on the studies by categorizing and grouping them into Asia, Europe, and the United States and Australia.

We performed the meta-analysis using R (version 4.0.0, The R Foundation for Statistical Computing, Vienna, Austria), with the packages “meta” (version 4.11-0) and “metaphor” (version 2.1-0). A *p*-value less than 0.05 was considered statistically significant. Publication bias was assessed using a funnel plot. Asymmetry of the funnel plot was an indicator of bias.

## Results

### Study selection

**Figure 1:**
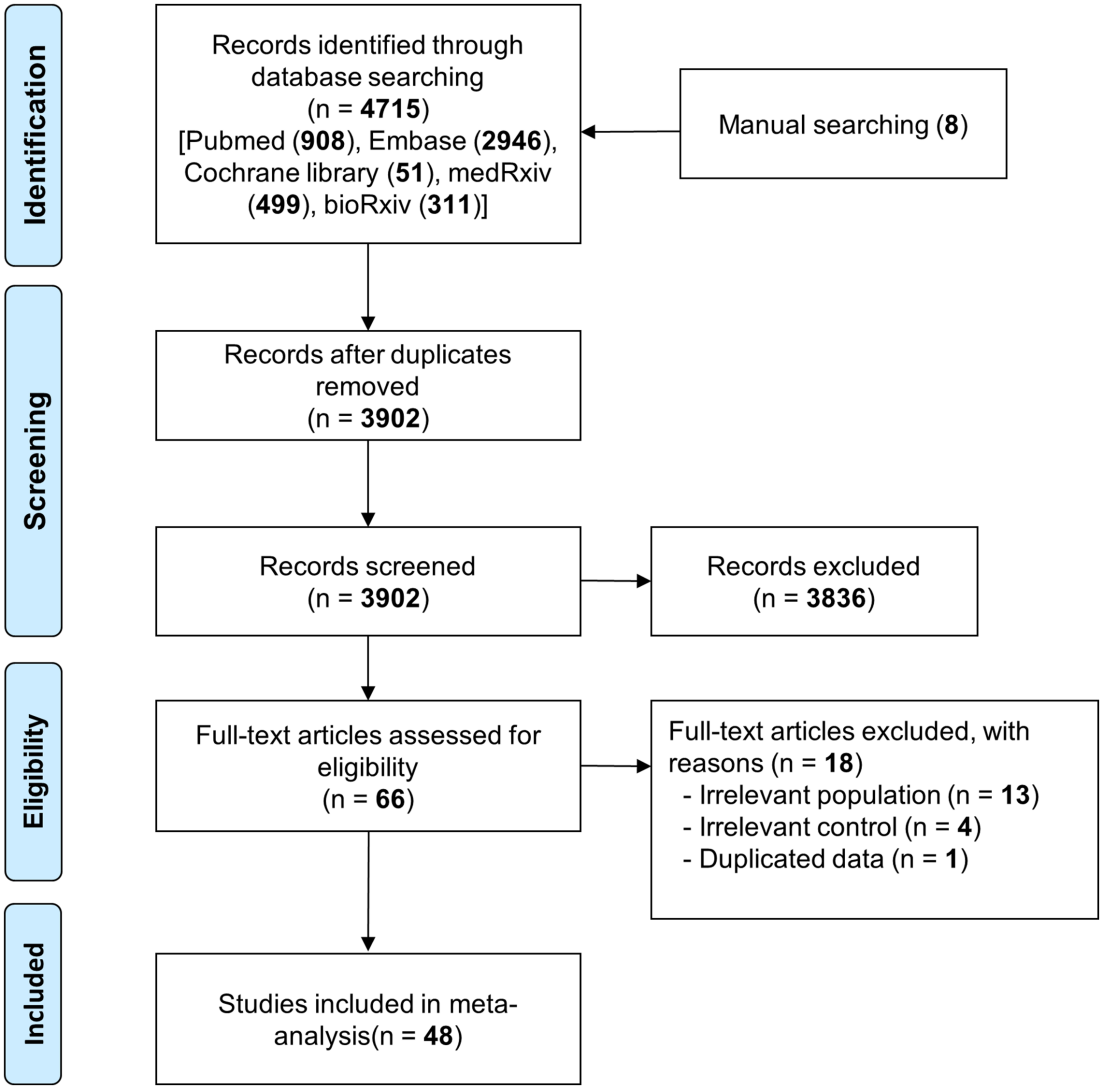
PRISMA flow diagram.

A total of 4,715 articles were searched from five databases, and eight were identified through manual searches. In total, 4,723 articles were found. After excluding duplicate searches by title, author, and year of publication, 3,902 articles were subjected to primary exclusion based on a review of the title and abstract. Sixty-six articles were identified to be potentially related to the research topic and underwent a full-text review for secondary exclusion. A total of 18 documents were excluded during the secondary exclusion review for the following reasons: the type of study met the exclusion criteria (9 cases: reviews), the study did not compare data from before the COVID-19 pandemic (4 studies), the results of the studies were inconsistent with the objectives of this study (3 studies compared myocardial infarction and in-hospital cardiac arrest), overlapping participants (one study whose authors used the same sample in subsequent studies with overlapping variables), and inadequate information about the comparison or control group (one study did not have relevant data on the comparison of the COVID-19 pandemic with the pre-pandemic period). Finally, 48 articles were included in this systematic review and meta-analysis (Figure 1) (13, 34–80).

### Study characteristics

The main characteristics and basic patient characteristics from the 48 studies included in this systematic review and meta-analysis are described in Table 1. The timing of COVID-19 in the included studies is presented in Supplementary Figure S1.

**Table 1:** Baseline characteristics of the study populations included in this review.

Study	Location	Study design	Before the COVID-19 pandemic				During the COVID-19 pandemic			
			Study period	Sample size	Male, *N* (%)	Age, years	Study period	Sample size	Male, *N* (%)	Age, years
Ahn 2021	Daegu, Republic of Korea	Registry based	February 18–April 17, 2019	145	91 (62.8%)	74.0 (61.5–82.0)	February 18–April 17, 2020	152	102 (67.1%)	76.0 (66.0–81.8)
Baert 2020	France	Registry based	March 1–April 31, 2019	1,620	1,071 (66.1%)	69.0 ± 17.0	March 1–April 31, 2020	1,005	676 (67.3%)	68.0 ± 17.0
Baldi 2020 (A)	Lombardy, Italy	Registry based	February 21–April 1, 2019	229	138 (60.3%)	79.0 (67.0–86.0)	February 21–March 31, 2020	362	237 (65.5%)	77.0 (67.0–84.0)
Baldi 2020 (B)	Lombardy, Italy	Registry based	February 21–April 20, 2019	321	188 (58.6%)	79.0 (67.0–86.0)	February 21–April 20, 2020	490	321 (65.5%)	78.0 (67.0–84.0)
Baldi 2020 (C)	Lombardy, Italy	Registry based	February 21–May 31, 2019	520	300 (57.7%)	79.0 (65.0–86.0)	February 21–May 30, 2020	694	430 (62.0%)	77.0 (67.0–85.0)
Baldi 2021 (A)	Switzerland	Registry based	February 25–April 30, 2019	933	636 (68.2%)	71.0 (58.0–82.0)	February 25–April 30, 2020	911	623 (68.4%)	70.0 (56.0–80.0)
Baldi 2021 (B)	Canton, Switzerland	Registry based	March 3–June 26, 2016–2019	398	256 (64.3%)	74.0 (60.0–83.0)	March 3–June 26, 2020	203	145 (71.4%)	74.0 (61.0–82.0) 74.0 (57.0–81.0)
Ball 2020	Victoria, Australia	Registry based	March 16–May 12, 2017–2019	1,218	845 (69.4%)	67.0 (52.0–78.0)	March 16–May 12, 2020	380	250 (65.8%)	69.0 (54.0–80.0)
Biskupski 2022	South Bronx, New York City, United States	Non-registry based	August 1, 2019–February 28, 2020	28	17 (60.7%)	65.0	March 1, 2020–June 30, 2021	86	52 (60.5%)	59.0
Burn 2022	Montgomery County, United States	Registry based	July 1, 2019–February 28, 2020	499	293 (58.7%)	67.6 ± 20.6	July 1, 2020–February 28, 2021	617	376 (60.9%)	67.2 ± 19.9
Chan 2021	27 States and multiple Counties, United States	Registry based	March 16–April 30, 2019	9,440	5,922 (62.7%)	62.2 ± 19.2	March 16–April 30, 2020	9,863	6,040 (61.2%)	62.6 ± 19.3
Chavez 2022	Texas, United States	Registry based	March 11–December 31, 2019	3,619	2,307 (63.7%)	63.0 (51.0–74.0)	March 11–December 31, 2020	4,418	2,781 (62.9%)	63.0 (51.0–74.0)
Cho 2020	Daegu, Republic of Korea	Registry based	February 17–March 31, 2018	158	103 (65.2%)	74.3 (61.8–82.2)	February 17–March 31, 2020	171	108 (63.2%)	74.0 (62.0–80.8)
Chung 2021	Republic of Korea	Non-registry based	January 1–December 31, 2019	129	79 (61.2%)	71.2 ± 14.6	March 1, 2020–February 28, 2021	101	65 (64.4%)	68.2 ± 17.8
Coute 2022	United States	Registry based	January 1–December 31, 2016–2019	316,309	196,589 (62.2%)	63.8 ± 16.9	January 1–December 31, 2020	124,129	77,211 (62.2%)	63.6 ± 17.0
Damjanovic 2022	Freiburg, Germany	Non-registry based	February 27–April 30, 2016–2019	102	68 (66.7%)	68.9 (57.4–79.7)	February 27–April 30, 2020	24	15 (62.5%)	67.9 (58.7–84.2)
de Koning 2021	Hollands-Midden, The Netherlands	Registry based	March 16–April 27, 2019	45	31 (68.9%)	70.0 ± 12.0	March 16–April 27, 2020	56	32 (57.1%)	70.0 ± 14.0
Elmer 2020	Pennsylvania, United States	Registry based	January–February 2016–2020	12,252	7,700 (62.8%)	63.0 ± 19.0	March 1–May 25, 2020	683	430 (63.0%)	64.0 ± 19.0
Fothergill 2021	London, UK	Registry based	March 1–April 30, 2019	1724	1,069 (62.0%)	68.0 ± 20.0	March 1–April 30, 2020	3,122	1839 (58.9%)	71.0 ± 19.0
Glober 2021	Indiana (Marion County), United States	Registry based	January 1–June 30, 2019	884	544 (61.5%)	62.4 (48.8–73.2)	January 1–June 30, 2020	1,034	622 (60.2%)	60.3 (46.9–71.8)
Grübl 2021	Marburg, Germany	Non-registry based	January 1–May 31, 2018–2019	173,149	207 (64.3%)	70.0 ± 15.0 69.0 ± 18.0	January 1–May 31, 2020	175	120 (68.6%)	70 ± 15
Hosomi 2022	Japan	Registry based	January 1–December 31, 2019	39,324	23,593 (60.0%)	79.0 (69.0–87.0)	January 1–December 31, 2020	39,170	23,685 (60.5%)	79.0 (69.0–87.0)
Kandori 2021	Kyoto, Japan	Non-registry based	January 1, 2019–March 31, 2020	267	164 (61.4%)	77.0 (65.0–85.0)	April 1–December 31, 2020	176	97 (55.1%)	76.0 (64.0–84.0)
Lai 2020	New York City, United States	Non-registry based	March 1–April 25, 2019	1,336	752 (56.3%)	68.0 ± 19.0	March 1–April 25, 2020	3,989	2,183 (54.7%)	72.0 ± 18.0
Lee 2021	Daejeon, Republic of Korea	Non-registry based	February 1–October 31, 2019	492	NR	NR	February 1–October 31, 2020	538	NR	NR
Lim 2021 (A)	Busan, Ulsan, Gyeongnam, and Changwon, Republic of Korea	Registry based	November 1, 2019–January 31, 2020	891	577 (64.8%)	70.1 ± 15.1	November 1, 2020–January 31, 2021	1,063	647 (60.9%)	71.1 ± 15.0
Lim 2021 (B)	Singapore	Registry based	January 1–May 31, 2018–2019	2,493	1,597 (64.1%)	71.0 ± 3.8	January 1–May 31, 2020	1,400	882 (63.0%)	72.5 ± 4.0
Lim 2022 (A)	Republic of Korea	Registry based	January 26–June 30, 2016–2019	628	490 (78.0%)	NR	January 26–June 30, 2020	160	135 (84.4%)	NR
Lim 2022 (B)-1	Singapore	Registry based	January 23–May 20, 2019	963	612 (63.6%)	72.0 (60.0–83.0)	January 23–May 20, 2020	1,012	654 (64.6%)	73.0 (61.0–84.0)
Lim 2022 (B)-2	Atlanta, United States	Registry based	March 2–June 28, 2019	937	549 (58.6%)	66.0 (54.0–77.0)	March 2–June 28, 2020	1,072	581 (54.2%)	66.0 (54.0–76.0)
Marijon 2020	Paris, France	Registry based	Weeks 12–17, 2012–2019	3,052	1826 (59.8%)	68.5 ± 18.0	March 16–April 26, 2020	521	334 (64.1%)	69.7 ± 17
Mathew 2021	Detroit, United States	Registry based	March 10–April 30, 2019	180	93 (51.7%)	58.5 ± 19.8	March 10–April 30, 2020	291	165 (56.7%)	64.5 ± 18.1
Navalpotro 2021 (A)	Marid, Spain	Registry based	March 15, 2019–March 14, 2020	1,781	1,178 (66.1%)	72.0 (59.0–82.0)	March 15, 2020–March 14, 2021	1,743	1,117 (64.1%)	71.0 (57.0–81.0)
Navalpotro 2021 (B)	Marid, Spain	Registry based	March 1–April 30, 2019	306	199 (65.0%)	72.0 (60.0–83.0)	March 1–April 30, 2020	313	189 (60.4%)	72.0 (62.0–81.0)
Ng 2021	Singapore	Non-registry based	April 1–May 31, 2018–2019	1,034	NR	73.2 ± 4.0	April 1–May 31, 2020	493	NR	72.4 ± 4.0
Nickles 2021	Detroit (Macomb, Oakland, and Wayne Counties), United States	Registry based	January 1–May 31, 2019	1,162	662 (57.0%)	NR	January 1–May 31, 2020	1,854	1,083 (58.4%)	NR
Nishiyama 2021	Osaka, Japan	Registry based	February 1–July 31, 2019	862	551 (63.9%)	75.0 (63.0–83.0)	February 1–July 31, 2020	825	529 (64.1%)	77.0 (66.0–85.0)
Nishiyama 2022	Osaka, Japan	Registry based	January 1–December 31, 2019	2,420	1,403 (58.0%)	78.0 (68.0–86.0)	January 1–December 31, 2020	2,371	1,384 (58.4%)	80.0 (70.0–87.0)
Ortiz 2020	Spain	Registry based	April 1–30, 2017 and February 1–March 31, 2018	1,723	1,210 (70.2%)	65.6 ± 16.9	February 1–April 30, 2020	1,446	1,028 (71.1%)	64.4 ± 16.5
Paoli 2020	Province of Padua, Italy	Non-registry based	March 1–April 30, 2019	206	98 (47.6%)	77.0 ± 14.0	March 1–April 30, 2020	200	89 (44.5%)	79.0 ± 17.0
Phattharapornjaroen 2022	Bangkok, Thailand	Registry based	March 1, 2018–December 31, 2019	76	46 (60.5%)	70.0 ± 17.5	March 1, 2020–December 31, 2021	60	33 (55.0%)	65.4 ± 19.4
Ristau 2022	Germany, Austria, Switzerland	Registry based	March 1, 2018–February 28, 2019	8,962	5,601 (62.5%)	69.7 ± 16.9	March 1, 2020–February 28, 2021	9,837	6,256 (63.6%)	69.7 ± 16.6
Riyapan 2022	Thailand	Registry based	January 1–September 30, 2019	341	210 (61.6%)	62.7 ± 18.5	January 1–September 30, 2020	350	208 (59.4%)	63.4 ± 19.4
Sayre 2020	Seattle and King County, Washington, United States	Registry based	January 1–February 25, 2019	530	NR	NR	February 26–April 15, 2020	537	NR	NR
Semeraro 2020	Bologna, Italy	Registry based	January 1–June 30, 2019	563	284 (50.4%)	84.0 (73.0–91.0)	January 1–June 30, 2020	624	318 (51.0%)	84.0 (73.0–91.0)
Sultanian 2021	Sweden	Registry based	January 1–March 16, 2020	930	604 (64.9%)	70.8 ± 16.6	March 16–July 20, 2020	1,016	697 (68.6%)	69.6 ± 17.8
Sun 2021	Boston, United States	Registry based	March 15–June 8, 2018–2019	440	269 (61.1%)	66.0	March 15–June 8, 2020	298	187 (62.8%)	65.0
Talikowska 2021	Western Australia, Australia	Registry based	March 16–May 17, 2017–2020	501	345 (68.9%)	60.0 (46.0–74.0)	March 16–May 17, 2020	145	101 (69.7%)	61.0 (46.0–74.0)
Uy-Evanado 2021	Oregon (Multnomah County) and California (Ventura County), United States	Registry based	March 1–May 31, 2019	231	137 (59.3%)	69.1 ± 17.4	March 1–May 31, 2020	278	174 (62.6%)	69.4 ± 18.3
Yu 2021	Taichung, Taiwan	Registry based	February 1–April 30, 2019	570	353 (61.9%)	70.3 ± 16.5	February 1–April 30, 2020	622	394 (63.3%)	70.4 ± 16.2

Values are shown as the mean ± standard deviation, median (interquartile range), or frequency (proportion). In cases where regional data were used, the name of the city is mentioned only if it was specified in the study; otherwise, only the country is listed. NR, not reported.

Thirty-four studies only included data from the first wave of COVID-19, and fourteen studies included data from the second and subsequent waves of COVID-19. Studies were grouped into four regional categories: fourteen studies conducted in the Republic of Korea, Japan, Thailand, Taiwan, and Singapore were grouped in the Asia group; nine studies were conducted in Europe; and the remain studies were classified into the United States and Australia group. The study by Lim et al. compared OHCAs during and before the COVID-19 pandemic in Singapore and Atlanta, United States, the data from these two locations were collected and analyzed separately (58).

### Quality assessment of the included studies

The risk of bias was assessed using ROBINS-I for the 49 studies included in this systematic review and meta-analysis (Supplementary Figures S2, S3). Bias occurred mainly before the intervention (domains 1 and 2) and during the intervention (domain 3). Domain 1 was assessed as low risk of bias if the patient’s baseline, disease, history, and predefined confounding factors were presented clearly and as moderate or high risk when this information was not provided. Only 7 of the 48 studies were rated as low risk. The risk of bias (domain 3) of intervention was assessed as low if the duration of the intervention was presented with clear criteria and moderate if this was not presented. In addition, when 2020 was included in the control period or if no clear classification criteria were proposed, the risk was rated as moderate or high. For the post-intervention (domains 4–7) items, all the studies were assessed as low risk.

### Total population: during versus before the COVID-19 pandemic

**Figure 2:**
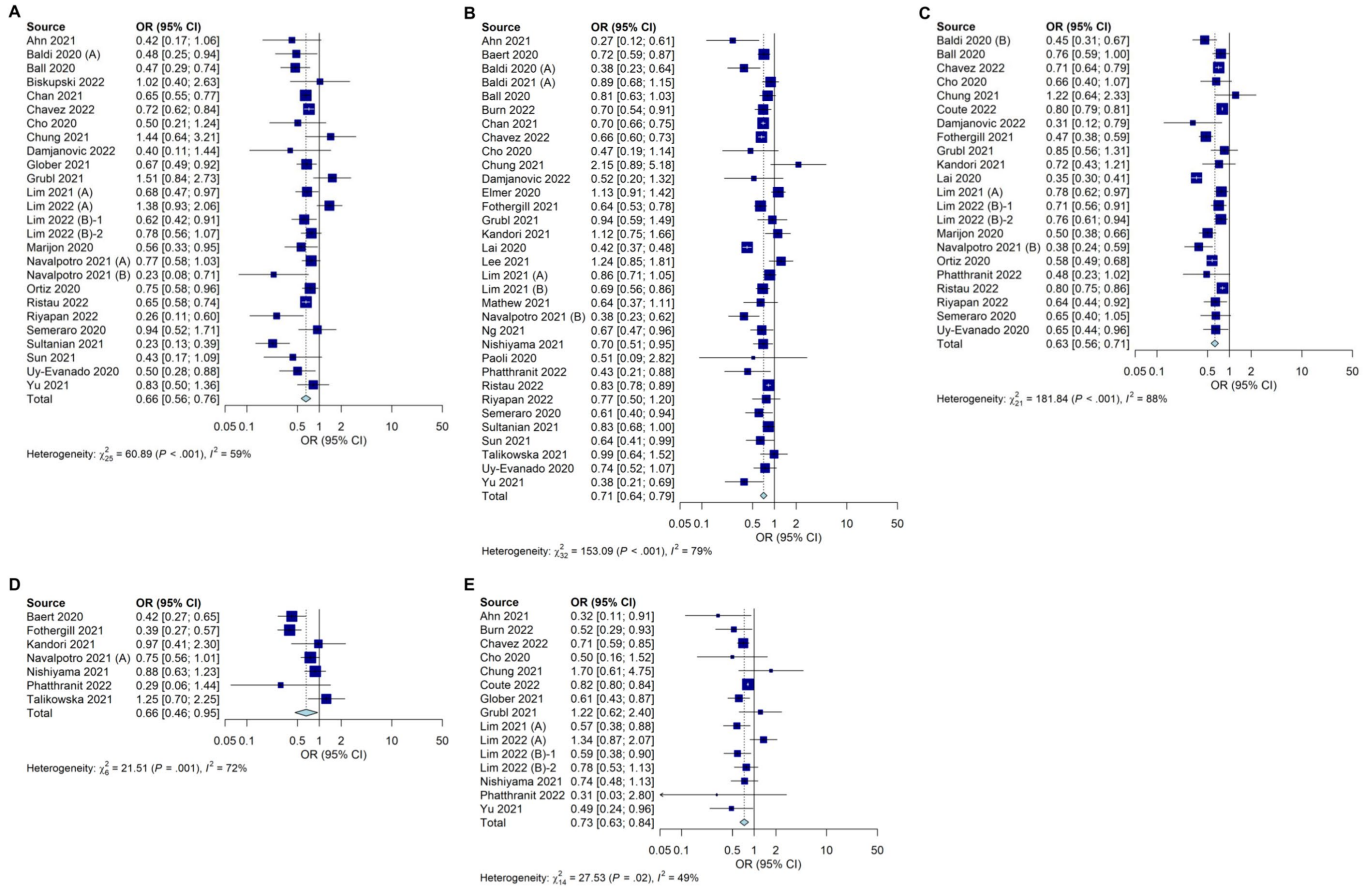
Forest plot depicting the outcomes of OHCA during the COVID-19 pandemic compared with those before the pandemic. **(A)** Live discharge rates **(B)** Spontaneous circulatory recovery rates **(C)** Survival to hospital admission rates **(D)** 30-day survival **(E)** Neurological prognoses.

A total of 26 studies showed a statistically significant reduction in live discharge rates during the COVID-19 pandemic (OR, 0.66; 95% CI, 0.56–0.76; *I*^2^ = 59%; Figure 2A). Spontaneous circulatory recovery rates decreased significantly during the COVID-19 pandemic in 33 studies (OR, 0.71; 95% CI, 0.64–0.79; *I*^2^ = 79%; Figure 2B). Survival to hospital admission rates decreased significantly during the COVID-19 pandemic in 22 studies (OR, 0.63; 95% CI, 0.56–0.71; *I*^2^ = 88%; Figure 2C). Seven studies showed a statistically significant reduction in 30-day survival during the COVID-19 pandemic (OR, 0.66; 95% CI, 0.46–0.95; *I*^2^ = 72%; Figure 2D). Neurological prognoses were significantly worse during the COVID-19 pandemic in 15 studies (OR, 0.73; 95% CI, 0.63–0.84; *I*^2^ = 49%; Figure 2E).

**Figure 3:**
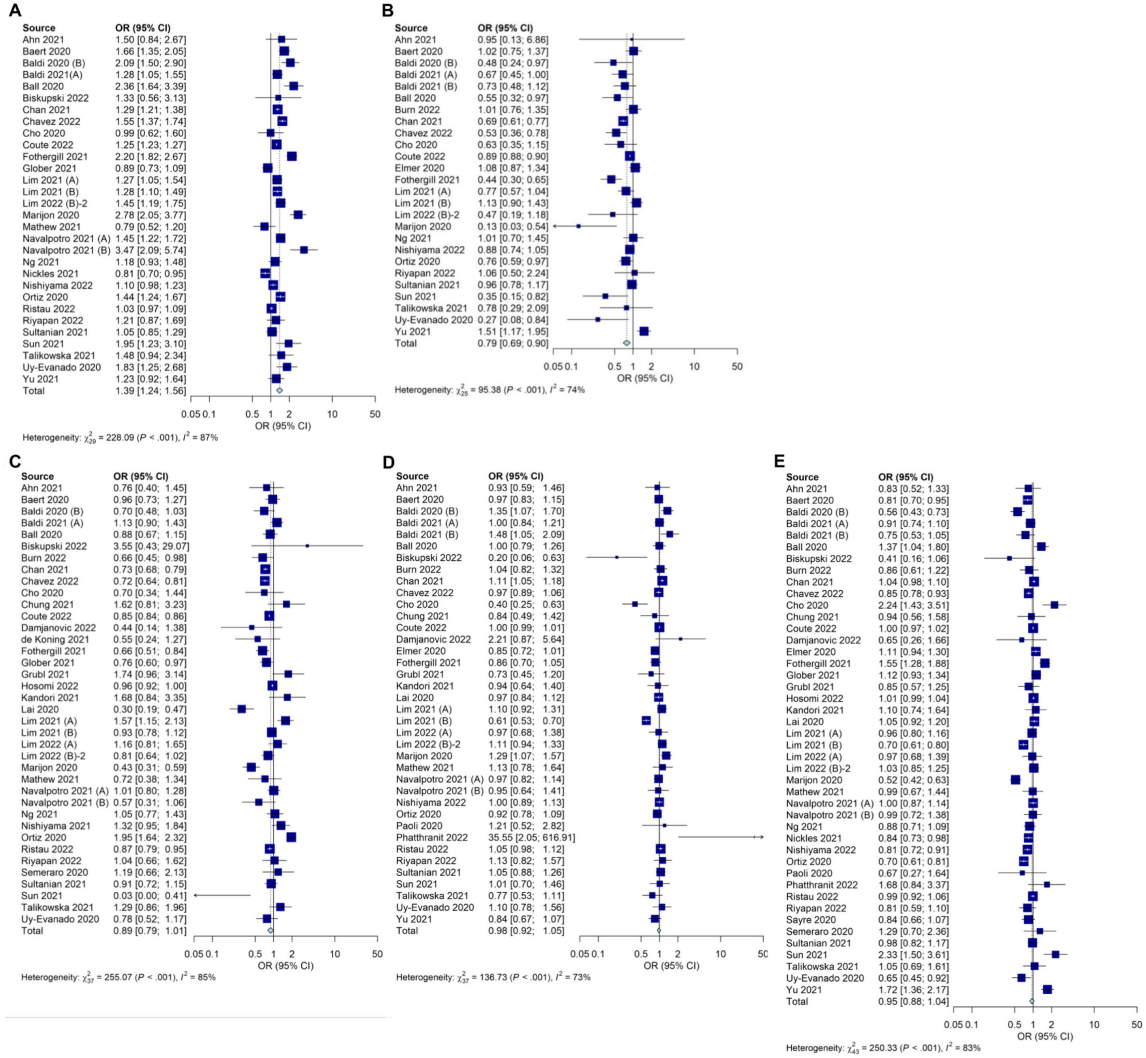
Forest plot depicting the epidemiologic factors of OHCA during the COVID-19 pandemic compared with those before the pandemic. **(A)** Incidence of cardiac arrest at home **(B)** Use of AEDs **(C)** Rhythm of shock needs **(D)** Number of unwitnessed cardiac arrests **(E)** Rate of bystander CPR.

The incidence of cardiac arrest at home was increased significantly during the COVID-19 pandemic in 30 studies (OR, 1.39; 95% CI, 1.24–1.56; *I*^2^ = 87%; Figure 3A). The use of AEDs was significantly decreased during the COVID-19 pandemic in 26 studies (Figure 3B; OR, 0.79; 95% CI, 0.69–0.90; *I*^2^ = 74%) and the rhythm of shock needs was not significantly different in 38 studies, which depicted a trend that differed from previous meta-analyses (Figure 3C; OR, 0.89; 95% CI, 0.79–1.01; *I*^2^ = 85%). The number of unwitnessed cardiac arrests did not differ significantly between during the COVID-19 pandemic and before the COVID-19 period in 38 studies (Figure 3D; OR, 0.98; 95% CI, 0.92–1.05; *I*^2^ = 73%). The rate of bystander CPR being performed did not differ significantly between during the COVID-19 pandemic and before the COVID-19 pandemic in 44 studies (Figure 3E; OR, 0.95; 95% CI, 0.88–1.04; *I*^2^ = 83%).

**Figure 4:**
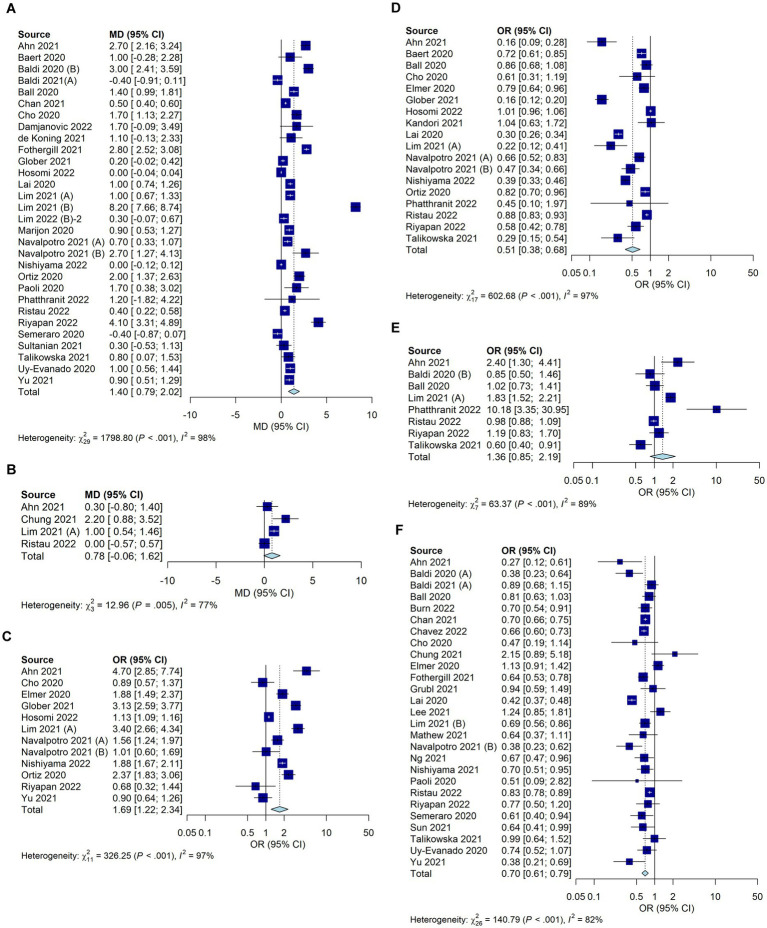
Forest plot depicting the prehospital factors of OHCA during the COVID-19 pandemic compared with those before the pandemic. **(A)** EMS response time **(B)** EMS transport time **(C)** Use of supraglottal airway devices **(D)** Endotracheal intubation **(E)** CPR with mechanical CPR devices **(F)** Prehospital spontaneous circulatory recovery rates.

The EMS response time was significantly longer during the COVID-19 pandemic in 30 studies (mean difference, 1.40; 95% CI, 0.79–2.02; *I*^2^ = 98%; Figure 4A). There was no significant difference in the EMS transport time in four studies (mean difference, 0.78; 95% CI, −0.06 to 1.62; *I*^2^ = 77%; Figure 4B). The use of supraglottal airway devices increased significantly during the pandemic in 12 studies (Figure 4C; OR, 1.69; 95% CI, 1.22–2.34; *I*^2^ = 97%), whereas endotracheal intubation, as analyzed in 18 studies, decreased significantly (Figure 4D; OR, 0.51; 95% CI, 0.38–0.68; *I*^2^ = 97%). Data on CPR with mechanical CPR devices were included in eight studies and did not differ significantly between during the COVID-19 pandemic and before the COVID-19 pandemic (Figure 4E; OR, 1.36; 95% CI, 0.85–2.19; *I*^2^ = 89%). Prehospital spontaneous circulatory recovery rates were significantly reduced during the COVID-19 pandemic in 27 studies (OR, 0.70; 95% CI, 0.61–0.79; *I*^2^ = 81%; Figure 4F).

There was no significant difference in the target body temperature treatment between during the COVID-19 pandemic and before the COVID-19 pandemic in seven studies (OR, 0.64; 95% CI, 0.37–1.10; *I*^2^ = 52%; Supplementary Figure S4).

### Subgroup analysis according to study period during the pandemic: during versus before the COVID-19 pandemic

The entire analysis was divided into studies that included studies with first wave data only (A) and studies with sequential wave periods with and without the first wave (B), both of which showed a significant reduction in the survival discharge rates compared with the pre-pandemic period (OR, 0.63; 95% CI, 0.51–0.77; *I*^2^ = 64% and OR, 0.69; 95% CI, 0.63–0.75; *I*^2^ = 44%, respectively; Table 2; Supplementary Figure S5). Spontaneous circulatory recovery rates decreased significantly in (A) and (B) compared with the pre-pandemic periods (OR, 0.66; 95% CI, 0.58–0.75; *I*^2^ = 78% and OR, 0.83; 95% CI, 0.71–0.98; *I*^2^ = 74%, respectively; Table 2; Supplementary Figure S5). Survival to hospital admission and favorable neurological prognosis rates decreased significantly in (A) and (B) compared with the pre-pandemic period [(A) OR, 0.56; 95% CI, 0.48–0.66; *I*^2^ = 79% and OR, 0.72; 95% CI, 0.56–0.93; *I*^2^ = 50%, respectively; (B) OR, 0.78; 95% CI, 0.74–0.82; *I*^2^ = 30% and OR, 0.74; 95% CI, 0.63–0.87; *I*^2^ = 49%, respectively; Table 2; Supplementary Figure S5].

**Table 2:** Subgroup analysis according to the inclusion period following the early outbreak period: comparison of outcomes during the COVID-19 pandemic versus before the pandemic.

Period	Number of studies	OR	95% CI	Heterogeneity	
				*I*^2^, %	*p*-value
**Outcomes**					
Survival to discharge					
Studies including 1st wave only^a^	19	0.63	0.51–0.77	64	<0.01
Studies including subsequent wave^b^	7	0.69	0.63–0.75	44	0.10
Return of spontaneous circulation					
Studies including 1st wave only^a^	24	0.66	0.58–0.75	78	<0.01
Studies including subsequent wave^b^	9	0.83	0.71–0.98	74	<0.01
Survival to admission					
Studies including 1st wave only^a^	14	0.56	0.48–0.66	79	<0.01
Studies including subsequent wave^b^	8	0.78	0.74–0.82	30	0.19
30-day survival					
Studies including 1st wave only^a^	19	0.63	0.51–0.77	64	<0.01
Studies including subsequent wave^b^	7	0.69	0.63–0.75	44	0.10
Favorable neurological outcome					
Studies including 1st wave only^a^	9	0.72	0.56–0.93	50	0.04
Studies including subsequent wave^b^	6	0.74	0.63–0.87	49	0.08
Pre ROSC					
Studies including 1st wave only^a^	21	0.65	0.56–0.75	80	<0.01
Studies including subsequent wave^b^	6	0.84	0.67–1.04	80	<0.01
**Epidemiologic factors**					
Arrest at home					
Studies including 1st wave only^a^	22	1.45	1.24–1.70	87	<0.01
Studies including subsequent wave^b^	8	1.25	1.11–1.40	88	<0.01
AED use					
Studies including 1st wave only^a^	20	0.75	0.62–0.90	77	<0.01
Studies including subsequent wave^b^	6	0.89	0.88–0.90	44	0.11
Shockable rhythm					
Studies including 1st wave only^a^	27	0.85	0.72–1.00	86	<0.01
Studies including subsequent wave^b^	11	0.97	0.82–1.14	85	<0.01
Unwitnessed arrest					
Studies including 1st wave only^a^	26	0.97	0.89–1.07	79	<0.01
Studies including subsequent wave^b^	12	1.00	0.99–1.01	39	0.08
Bystander CPR					
Studies including 1st wave only^a^	31	0.96	0.86–1.09	86	<0.01
Studies including subsequent wave^b^	13	0.95	0.89–1.01	66	<0.01
**Prehospital factors**					
EMS response time					
Studies including 1st wave only^a^	23	1.52	0.79–2.25	98	<0.01
Studies including subsequent wave^b^	8	0.93	−0.01 to 1.87	97	<0.01
EMS transport time					
Studies including 1st wave only^a^	1	–	–	–	–
Studies including subsequent wave^b^	3	0.95	−0.20 to 2.10	84	<0.01
Supraglottic airway use					
Studies including 1st wave only^a^	7	1.77	1.10–2.85	92	<0.01
Studies including subsequent wave^b^	5	1.58	0.98–2.55	97	<0.01
Endotracheal intubation					
Studies including 1st wave only^a^	10	0.44	0.29–0.67	96	<0.01
Studies including subsequent wave^b^	8	0.62	0.43–0.88	95	<0.01
Mechanical CPR					
Studies including 1st wave only^a^	4	1.03	0.60–1.77	78	<0.01
Studies including subsequent wave^b^	4	1.91	0.79–4.64	94	<0.01
**In-hospital factors**					
TTM					
Studies including 1st wave only^a^	3	0.48	0.21–1.09	62	0.07
Studies including subsequent wave^b^	4	0.84	0.36–1.95	49	0.12

AED, automated external defibrillator; CI, confidence interval; CPR, cardiopulmonary resuscitation; EMS, emergency medical services; OR, odds ratio; ROSC, return of spontaneous circulation; TTM, target temperature management.

a This group includes studies that reported data from only the 1st wave period.

b This group includes studies that reported data from the 1st as well as subsequent wave periods.

Furthermore, the incidence of cardiac arrest at home showed the same trend, with the same significant increase in (A) and (B), compared with the pre-pandemic period (OR, 1.45; 95% CI, 1.24–1.70; *I*^2^ = 87% and OR, 1.25; 95% CI, 1.11–1.40; *I*^2^ = 88%, respectively; Table 2; Supplementary Figure S6). The use of automatic external defibrillators decreased significantly in (A) and (B) compared with that of the pre-pandemic period (OR, 0.75; 95% CI, 0.62–0.90; *I*^2^ = 77% and OR, 0.89; 95% CI, 0.88–0.90; *I*^2^ = 44%, respectively; Table 2; Supplementary Figure S6).

The EMS response time increased significantly in period (A) compared with before the pandemic; however, no significant difference between period (B) and the pandemic period was observed (mean difference, 1.52; 95% CI, 0.79–2.25; *I*^2^ = 98% and OR, 0.93; 95% CI, −0.01 to −1.87; *I*^2^ = 97%, respectively; Table 2; Supplementary Figure S7). The use of supraglottal airway devices increased significantly in period (A) compared with before the pandemic, although this did not significantly differ from that in period (B) (OR, 1.77; 95% CI, 1.10–2.85; *I*^2^ = 92% and OR, 1.58; 95% CI, 0.98–2.55; *I*^2^ = 97%, respectively; Table 2; Supplementary Figure S7). Endotracheal intubation significantly decreased in (A) and (B) compared with before the pandemic (OR, 0.44; 95% CI, 0.29–0.67; *I*^2^ = 96% and OR, 0.62; 95% CI, 0.43–0.88; *I*^2^ = 95%, respectively; Table 2; Supplementary Figure S7). The spontaneous circulatory recovery rate at the prehospital stage decreased significantly in period (A) compared with the pre-pandemic period, whereas period (B) did not significantly differ compared with the pre-pandemic period (OR, 0.65; 95% CI, 0.56–0.75; *I*^2^ = 80% and OR, 0.84; 95% CI, 0.67–1.04; *I*^2^ = 80%, respectively; Table 2; Supplementary Figure S7).

Target temperature management in the hospital stage did not significantly differ in period (A) of period (B) compared with the pre-pandemic pandemic (OR, 0.48; 95% CI, 0.21–1.09; *I*^2^ = 62% and OR, 0.84; 95% CI, 0.36–1.95; *I*^2^ = 49%, respectively; Table 2; Supplementary Figure S8).

### Region-wise subgroup analysis: during versus before the COVID-19 pandemic

Survival discharge rates decreased significantly during the COVID-19 pandemic in Europe and the United States, whereas no significant difference was detected in Asia (OR 0.61, 95% CI 0.43–0.85, *I*^2^ = 71%, OR 0.69, 95% CI 0.62–0.76, *I*^2^ = 0%, and OR 0.71, 95% CI 0.49–1.02, *I*^2^ = 0%, respectively; Table 3; Supplementary Figure S9). In Asia, Europe, and North America, the total spontaneous recovery and survival hospitalization rates decreased significantly during the COVID-19 pandemic (ROSC: OR 0.73, 95% CI 0.57–0.93, *I*^2^ = 67%; OR 0.69, 95% CI 0.60–0.81, *I*^2^ = 61%; and OR 0.68, 95% CI 0.55–0.84, *I*^2^ = 90%, respectively. Survival to admission: OR 0.73, 95% CI 0.64–0.83, *I*^2^ = 0%; OR 0.56, 95% CI 0.46–0.68, *I*^2^ = 85%; and OR 0.63, 95% CI 0.46–1.86, *I*^2^ = 96%, respectively; Table 3; Supplementary Figure S9). The neurological prognosis in Asia and the United States significantly worsened during the COVID-19 pandemic (OR 0.69, 95% CI 0.50–0.95, *I*^2^ = 54%, and OR 0.74, 95% CI 0.65–0.85, *I*^2^ = 45%, respectively; Table 3; Supplementary Figure S9).

**Table 3:** Region-wise comparison of the data on survival analysis during the COVID-19 pandemic with before the pandemic.

Period	Number of studies	OR	95% CI	Heterogeneity	
				*I*^2^, %	*p*-value
**Outcomes**					
Survival to discharge					
Asia	8	0.71	0.49–1.02	67	<0.01
Europe	10	0.61	0.43–0.85	71	<0.01
US	7	0.69	0.62–0.76	0	0.65
Return of spontaneous circulation					
Asia	12	0.73	0.57–0.93	67	<0.01
Europe	12	0.69	0.60–0.81	61	<0.01
US	8	0.68	0.55–0.84	90	<0.01
Survival to admission					
Asia	7	0.73	0.64–0.83	0	0.60
Europe	9	0.56	0.46–0.68	85	<0.01
US	5	0.63	0.46–0.86	96	<0.01
30-day survival					
Asia	3	0.85	0.63–1.16	0	0.40
Europe	3	0.51	0.33–0.78	78	0.01
US	0	–	–	–	–
Favorable neurological outcome					
Asia	9	0.69	0.50–0.95	54	0.03
Europe	1	–	–	–	–
US	5	0.74	0.65–0.85	45	0.12
**Epidemiologic factors**					
Cardiac arrest at home					
Asia	8	1.18	1.10–1.27	0	0.71
Europe	11	1.57	1.23–2.00	93	<0.01
US	9	1.28	1.06–1.55	87	<0.01
AED use					
Asia	8	0.99	0.81–1.22	62	0.01
Europe	8	0.70	0.54–0.89	70	<0.01
US	8	0.72	0.55–0.94	83	<0.01
Shockable rhythm					
Asia	11	1.09	0.95–1.25	51	0.03
Europe	15	0.88	0.70–1.10	88	<0.01
US	11	0.72	0.63–0.82	81	<0.01
Unwitnessed arrest					
Asia	11	0.87	0.72–1.04	83	<0.01
Europe	14	1.05	0.96–1.14	49	0.02
US	11	1.01	0.96–1.07	64	<0.01
Bystander CPR					
Asia	13	1.02	0.86–1.22	85	<0.01
Europe	16	0.86	0.74–1.00	85	<0.01
US	14	0.98	0.89–1.07	73	<0.01
Prehospital factors					
EMS response time					
Asia	11	1.77	0.23–3.30	99	<0.01
Europe	16	1.50	0.84–2.16	96	<0.01
US	6	1.15	0.02–2.28	95	<0.01
EMS transport time					
Asia	3	1.09	0.22–1.96	58	<0.09
Europe	1	–	–	–	–
US	0	–	–	–	–
Supraglottic airway use					
Asia	7	1.54	0.91–2.61	97	<0.01
Europe	3	1.61	1.03–2.54	81	<0.01
US	2	2.44	1.48–4.01	91	<0.01
Endotracheal intubation					
Asia	8	0.48	0.29–0.78	96	<0.01
Europe	5	0.72	0.60–0.87	81	<0.01
US	3	0.33	0.13–0.83	98	<0.01
Mechanical CPR					
Asia	4	2.37	1.11–5.09	80	<0.01
Europe	2	0.98	0.88–1.09	0	0.61
US	0	–	–	–	–
Pre ROSC					
Asia	9	0.70	0.51–0.95	68	<0.01
Europe	9	0.66	0.53–0.82	70	<0.01
US	8	0.68	0.55–0.84	90	<0.01
**In-hospital factors**					
TTM					
Asia	4	0.81	0.27–2.44	50	<0.11
Europe	2	0.53	0.21–1.34	78	0.03
US	1	–	–	–	–

AED, automated external defibrillator; CI, confidence interval; CPR, cardiopulmonary resuscitation; EMS, emergency medical services; OR, odds ratio; ROSC, return of spontaneous circulation; TTM, target temperature management; US, United States.

In Asia, Europe, and North America, the number of cardiac arrests at home significantly increased during the COVID-19 pandemic (OR 1.18, 95% CI 1.10–1.27, *I*^2^ = 0%; OR 1.57, 95% CI 1.23–2.00, *I*^2^ = 93%; and OR 1.28, 95% CI 1.06–1.55, *I*^2^ = 0%, respectively; Table 3; Supplementary Figure S10). In all three regions, the EMS response times were significantly longer during the COVID-19 pandemic (mean difference 1.77, 95% CI 0.23–3.30, *I*^2^ = 99%; mean difference 1.50, 95% CI 0.84–2.16, *I*^2^ = 96%; and mean difference 1.15, 95% CI 0.02–2.28, *I*^2^ = 95%, respectively; Table 3; Supplementary Figure S10).

In Europe and the United States, the use of supraglottal airway devices significantly increased during the pandemic, whereas in Asia, the use did not significantly differ (OR 1.61, 95% CI 1.03–2.54, *I*^2^ = 81%; OR 2.44, 95% CI 1.48–4.01, *I*^2^ = 91%; and OR 1.54, 95% CI 0.91–2.61, *I*^2^ = 97%, respectively; Table 3; Supplementary Figure S11). In Asia, Europe, and the United States, endotracheal intubation rates significantly decreased during the COVID-19 pandemic (OR 0.48, 95% CI 0.29–0.78, *I*^2^ = 0%; OR 0.72, 95% CI 0.60–0.87, *I*^2^ = 0%; and OR 0.33, 95% CI 0.13–1.83, *I*^2^ = 98%, respectively; Table 3; Supplementary Figure S11). Prehospital spontaneous circulatory recovery rates significantly decreased during the COVID-19 pandemic (OR 0.70, 95% CI 0.51–0.95, *I*^2^ = 68%; OR 0.66, 95% CI 0.53–0.82, *I*^2^ = 70%; and OR 0.68, 95% CI 0.55–0.84, *I*^2^ = 90%, respectively; Table 3; Supplementary Figure S11).

In Europe and Asia, target temperature management in the hospital stage did not significantly differ compared with the pre-pandemic pandemic (OR, 0.53; 95% CI, 0.21–1.34; *I*^2^ = 78% and OR, 0.81; 95% CI, 0.27–2.44; *I*^2^ = 50%, respectively; Table 3; Supplementary Figure S12).

### Publication bias

The ROSC and prehospital ROSC showed asymmetric funnel plots for the variables that were assessed in this study from more than ten studies (Supplementary Figure S13).

## Discussion

The COVID-19 pandemic greatly impacted the survival chain of OHCA patients at each stage of the survival chain. This review analyzed the latest studies comparing OHCA during the pandemic with pre-pandemic periods and included subgroup analyses based on region and time. A meta-analysis of 48 studies showed significant reductions in survival discharge and 30-day survival rates during the pandemic. Previous studies after the first wave also showed reduced survival to discharge rates (20, 22, 23). A regional analysis showed significant reductions in the United States and Europe but not in Asia, where there was no significant difference in survival rates during the pandemic. This review is clinically significant as it provides a detailed analysis of the pandemic’s impact on OHCA patients, considering the latest studies and regional and time-specific characteristics. However, previous studies only analyzed data from the early pandemic period, so ongoing research is needed to fully understand the pandemic’s impact on OHCA patients. The findings of this review underscore the importance of a coordinated response to maintain the survival chain of OHCA patients during pandemics.

Previous research has shown that the impact of the COVID-19 pandemic on cardiac arrest can be categorized into direct and indirect effects (81). Direct factors include hypoxia induced by the respiratory disease itself, inflammatory reactions (cytokine storms, thrombosis, myocarditis, and arrhythmias), pulmonary embolism caused by thrombosis, acute coronary syndrome, and drug-induced arrhythmias (81–84). Suggested indirect factors include social lockdown and distancing measures, home quarantine, the reopening of the health care system, reduction of emergency testing and skills, overloading of the emergency and hospital systems, wearing of personal protective equipment, reduction in hospital staffing, delays in care, and more frequent situations of being at risk when alone (20, 23, 81). These factors are closely related to the number of initial COVID-19 cases, and it can be assumed that the impact on the health system and OHCA was lower because the number of COVID-19 patients in Asia and the Republic of Korea was relatively low compared with Europe and North America during the initial pandemic period. In the Republic of Korea, the initial increase in cases showed a different trend than the United States and Europe, indicating that the OHCA survival rate during the pandemic and pre-pandemic period was not significantly different (19).

Depending on the region and country, policies related to the COVID-19 pandemic have shown different approaches (85–87). Several countries sought to prevent the spread of the virus by implementing strict lockdowns to block the inflow and transmission from the beginning of the COVID-19 pandemic and surveillance and tracing strategies of the infected and suspected patients by utilizing IT technology (85, 86, 88). In particular, Asian countries, such as the Republic of Korea and Japan, tried to contain the entry and spread of COVID-19 through stronger controls in the early stages of the outbreak (88). These approaches led to more available medical resources that could afford to operate relatively efficiently, resulting in a lower impact of the COVID-19 pandemic on OHCA (19, 89).

As in a previous meta-analysis, there was a significant increase in cardiac arrests at home, which can be explained by the fact that more people were in quarantine during the COVID-19 pandemic and time spent at home increased due to strong social distancing (90). The increase in cardiac arrest at home is related to other variables. In the subgroup analysis of this study, except in Asia, the use of AEDs decreased significantly during the COVID-19 pandemic. Since AEDs are mostly installed in public places, a significant increase in cardiac arrest at home would also reduce the likelihood of their application. Leung et al. noted that the use of AEDs in public places might have been reduced as governmental social distancing and activity restrictions limited access to public places (91). Nishiyama et al. showed that the number of AED applications decreased during the first wave of the COVID-19 pandemic but increased in the subsequent waves (68). Even those who were able to apply an AED and perform CPR did not know how to respond to cardiac arrest patients in a pandemic-like situation, reducing the rate of CPR performance. The implementation of AEDs increased with the publication of guidelines recommending active treatment by the public. Therefore, the dissemination of clear guidelines from authorized institutions to general citizens may improve the active treatment of OHCA patients (68).

Willingness to perform CPR varied in studies on witnessed OHCA. While some studies reported decreased willingness due to COVID-19, others found no change (92, 93). Fear of infection may make witnesses hesitant, but the increase in OHCA at home may have increased opportunities for family members to provide CPR with less resistance to infection. In addition, unlike previous meta-analyses that showed a decrease in shockable rhythms during the pandemic (20–23), this study found no significant difference compared to the pre-pandemic period. The reduced fatality rate of COVID-19 patients may have led to a decrease in OHCA patients, which is not significantly different from the pre-pandemic period. The fatality rate peaked in April 2020 at 7.71% but gradually declined to 5.21% by July 1, 2020, and is continuing to decrease since then (94). The decline in the fatality rate may have led to a decrease in OHCA patients and shockable rhythms. These findings suggest that the impact of the pandemic on OHCA patients is multifaceted and related to both direct and indirect factors, as well as the timing and fatality rate of COVID-19.

The use of endotracheal intubation decreased significantly during the COVID-19 pandemic across all subgroups, while the use of supraglottic airway devices increased significantly in most subgroups in this study. Meanwhile, CPR with mechanical CPR devices increased significantly in the subgroup analysis of Asia. Temporary resuscitation guidelines were issued by the ILCOR, the American Heart Association, and the European Resuscitation Committee for cardiac arrest patients with suspected or confirmed COVID-19, aiming to reduce the risk of infection for rescuers and the number of rescuers involved in resuscitation (95–97). The guidelines recommend the use of personal protective equipment, the most skilled rescuer attempting tracheal intubation, the use of a video laryngoscope to be considered whenever possible, and supraglottal airway devices use if tracheal intubation is not possible. This study showed a decrease in endotracheal intubation during the COVID-19 pandemic, with an increase in the use of supraglottal airway devices. While guidelines do not suggest avoiding intubation, some regions had protocols that prioritized the use of supraglottal airway devices (34, 51, 67). Rapid transport without intubation has also been recommended in some studies (73). The risk of infection during intubation may not be significantly higher when wearing personal protective equipment, but endotracheal intubation may have been avoided at front-line rescue sites due to a lack of studies on the risk of infection during intubation in the early stages of the pandemic.

The regional subgroup analyses, excluding Asia, showed no significant difference in CPR using mechanical CPR devices during the COVID-19 pandemic. A total of ten studies were included in the analysis of CPR using mechanical CPR devices, with six studies showing significant results. Among them, a significant increase in CPR with mechanical CPR devices during the COVID-19 pandemic was reported in three studies (34, 57, 71), and a significant decrease was reported in the other studies (13, 78, 98). Talikowska et al. noted that the increase in the number of patients who stopped CPR in the field could be explained by the desire to follow local protocols, and a decrease in the number of people who required CPR using mechanical CPR devices (78). The two studies by Baldi et al. did not provide a specific explanation; however, there was no significant difference in the interruption of rescue by paramedics, and therefore, an explanation due to other factors is needed (13, 98). Even before the COVID-19 pandemic, the region had a relatively low rate of CPR applications using mechanical CPR devices compared with other regions. However, the number of samples was small, and it is possible that these results were due to problems with the distribution of automatic CPR devices or the adaptation of field crews. The American Heart Association and the European Resuscitation Committee recommend CPR using mechanical CPR devices to minimize the risk of infection during CPR (95–97). Furthermore, the Korean Society of Emergency Medicine recommends CPR using a mechanical CPR device if possible (99). In Asia, including the Republic of Korea, these guidelines may have been well reflected, suggesting an increase in CPR using mechanical CPR devices. Over the duration of the study, Ahn et al. suggested that CPR was performed before these guidelines were issued, but regional protocols reflected these aspects in advance (34). In the Republic of Korea, the continuous supply of mechanical CPR devices since 2014 and the ability of paramedics to use mechanical CPR devices without medical guidance may have led to an increase in CPR using mechanical CPR devices due to the ease of reflecting the above guidelines (57).

During the COVID-19 pandemic, an increase in the number of OHCA and the influx of OHCA in people with COVID-19 could be expected to have an impact on the rate of targeted body temperature treatment; however, the results of this analysis did not show a significant difference. There are no guidelines for targeted body temperature treatment for patients with confirmed or suspected COVID-19, but since droplets are generally not produced during the process of targeted body temperature treatment, it appears that front-line medical staff actively implemented targeted body temperature treatment in consideration of the benefits of the treatment. The frequency of percutaneous coronary intervention reported by Ahn et al., Riyapan et al., and Phattharapornjaroen et al. and the frequency of emergency coronary angiography reported by Sultanian et al. did not show significant differences before and during the COVID-19 pandemic. Therefore, it can be assumed that the impact of the COVID-19 pandemic on the hospital-stage treatments of OHCA patients may be low; however, interpretation is limited because meta-analyses were not performed due to the small number of studies (34, 71, 73, 76).

The following limitations of this review need to be noted. First, there was a lack of information about underlying conditions, such as patients’ medical history or cerebral performance category (CPC) scores before cardiac arrest, resulting in confounding bias. Second, this study did not differentiate between confirmed and non-confirmed COVID-19 cases. Since COVID-19 cases can be a factor in cardiac arrest, the failure to analyze these studies without differentiating them may cause biases when comparing the COVID-19 pandemic period to the previous period. Third, this review was based on data from 16 countries and did not include studies from Africa and South America. In addition, the European studies focused on Western European countries, and the North American and other studies only included studies conducted in the United States and Australia. These limitations make it difficult to generalize the results of this study to races and countries worldwide. Fourth, this study conducted a meta-analysis considering the timing of the epidemic and the epidemic region as factors that influenced the pattern of the COVID-19 pandemic. In addition, the introduction of COVID-19 vaccines and major virus subvariants may have significantly changed the pandemic pattern, but none of the included studies conducted a meta-analysis on this topic. Finally, this is a systematic review and meta-analysis based on previously published non-randomized controlled studies and has a limited ability to reflect overall trends. Additionally, insignificant or negative research results are not always published, which can lead to bias.

## Conclusion

The COVID-19 pandemic has had a significant impact on the epidemiology and outcomes of OHCA patients. This review identified changes, such as an increase in cardiac arrests at home, a decrease in the use of AED, a decrease in endotracheal intubation, and an increase in the use of supraglottal airway devices. Unlike previous reviews, we noted a decrease in shockable rhythms. The impact of the pandemic on OHCA patients varied regionally, likely due to differences in health systems and resources. Some countries showed no significant differences in OHCA survival and neurological prognosis compared to before the pandemic, possibly due to a less explosive increase in COVID-19 cases and the absence of a complete collapse of the emergency medical care system. In the event of future infectious disease pandemics, the experiences and lessons learned globally from the pandemic will enable early recognition of factors that can negatively impact OHCA survival, improving the prognosis for OHCA patients and the effectiveness of the response.

## Author contributions

JK and CA developed the concept and drafted the manuscript. JK, CA, and YP performed the data analysis. JK and MW performed data acquisition. CA and YP performed the statistical analysis. All authors contributed to the article and approved the submitted version.

## Funding

Corredpondence (CA) have been supported by National Research Foundation of Korea (NRF) grant that was funded by the Korean Government (MSIT; 2021R1G1A1091336).

## Conflict of interest

The authors declare that the research was conducted in the absence of any commercial or financial relationships that could be construed as a potential conflict of interest.

## Publisher’s note

All claims expressed in this article are solely those of the authors and do not necessarily represent those of their affiliated organizations, or those of the publisher, the editors and the reviewers. Any product that may be evaluated in this article, or claim that may be made by its manufacturer, is not guaranteed or endorsed by the publisher.

## Abbreviations

AED: automated external defibrillator
CI: confidence interval
CPR: cardiopulmonary resuscitation
EMS: emergency medical services
OHCA: out-of-hospital cardiac arrest
OR: odds ratio
ROSC: return of spontaneous circulation
TTM: target temperature management
