# Supplementary material for: Comparison of out-of-hospital cardiac arrests during the COVID-19 pandemic with those before the pandemic: an updated systematic review and meta-analysis
Source: Front Public Health. 2023 May 4;11:1180511. doi: 10.3389/fpubh.2023.1180511 (PMC10208072; doi:10.3389/fpubh.2023.1180511)
Supplement: Supplementary file 1 [file table_1.docx]

**Supplementary file**

**Supplementary Table 1**. Variables according to each stage on the chain of survival

**Supplementary Table 2.** Search strategy

**Supplementary Table 3.** List of 48 included studies in meta-analysis

**Table S4.** GRADE profile for assessing quality of evidence for the included studies for outcomes

**Supplementary Figure 1.** Study period during COVID-19 pandemic of each study

**Supplementary Figure 2.** Plot of the distribution for each item from included studies.

**Supplementary Figure 3.** A summary table for assessment of each study.

**Supplementary Figure 4.** Forest plot for target temperature management during the pandemic comparing to before the pandemic.

**Supplementary Figure 5.** Forest plot depicting outcome during the COVID-19 pandemic compared with that before the pandemic, subgroup analysis according to study period of pandemic.

**Supplementary Figure 6.** Forest plot of epidemiologic factors during the COVID-19 pandemic compared with that before the pandemic, and subgroup analysis according to the study period during the pandemic.

**Supplementary Figure 7.** Forest plot for prehospital factors during the COVID-19 pandemic compared with that before the pandemic, and subgroup analysis according to study period during the pandemic.

**Supplementary Figure 8.** Forest plot for target temperature management during the COVID-19 pandemic compared with that before the pandemic, and subgroup analysis according to the study period of the pandemic.

**Supplementary Figure 9.** Forest plot for outcomes during the COVID-19 pandemic compared with those before the pandemic, and region-wise subgroup analysis during the pandemic.

**Supplementary Figure 10.** Forest plot for epidemiologic factors during the COVID-19 pandemic compared with that before the pandemic, and subgroup analysis according to the study region during pandemic.

**Supplementary Figure 11.** Forest plot depicting prehospital factors during the COVID-19 pandemic compared with that before the pandemic, and region-wise subgroup analysis of the pandemic period.

**Supplementary Figure 12.** Forest plot for target temperature management during the COVID-19 pandemic compared with that before the pandemic, and region-wise subgroup analysis of the pandemic period.

**Supplementary Figure 13.** Publication bias was calculated according to each outcome and factor.

**Supplementary Table 1**. Variables according to each stage on the chain of survival

| Chain of survival | Variables |
| --- | --- |
| Activation of emergency response | Unwitnessed arrest  Arrest at home  EMS response time^a^  EMS transport time^b^ |
| High-Quality CPR | Bystander CPR |
| Defibrillation | AED use  Shockable rhythm |
| Advanced resuscitation | Endotracheal intubation  Supraglottic airway use  Mechanical CPR  ROSC |
| Post-cardiac arrest care | Survival to admission  TTM |
| Recovery | Survival to discharge  30-day survival  Favorable neurological outcome |

Abbreviation: EMS, Emergency medical services; CPR, Cardiopulmonary resuscitation; AED, Automated external defibrillator; ROSC, Return of spontaneous circulation; TTM, Targeted temperature management

^a^EMS response time: time from emergency call to EMS arrival at the scene, ^b^EMS transport time: time from EMS departure to arrival at emergency department

**Supplementary Table 2.** Search strategy

(Search on October 23, 2022)

| Database | Search term |
| --- | --- |
| Pubmed | ((sudden cardiac arrest[Title/Abstract]) OR (sudden cardiac death[Title/Abstract]) OR (cardiac arrest[Title/Abstract]) OR (out-of-hospital cardiac arrest[Title/Abstract]) OR (out of hospital cardiac arrest[Title/Abstract]) OR (OHCA[Title/Abstract]) OR (OOHCA[Title/Abstract]) OR (CPR[Title/Abstract]) OR (cardiopulmonary resuscitation[Title/Abstract]) OR ("Cardiopulmonary Resuscitation"[Mesh]) OR ("Death, Sudden, Cardiac"[Mesh]) OR ("Out-of-Hospital Cardiac Arrest"[Mesh]) OR ("heart arrest"[Mesh]))  AND  ((covid-19[Title/Abstract]) OR ("coronavirus disease-19"[Title/Abstract]) OR (coronavirus[Title/Abstract]) OR ("COVID-19"[Mesh]) OR ("SARS-CoV-2"[Mesh]) OR (SARS-CoV-2[Title/Abstract])) |
| Embase | (‘cardiac arrest’:ti,ab,kw OR ‘sudden cardiac arrest’:ti,ab,kw OR ‘sudden cardiac death’:ti,ab,kw OR ‘out of hospital cardiac arrest’:ti,ab,kw OR ‘out-of-hospital cardiac arrest’:ti,ab,kw  OR OOHCA:ti,ab,kw OR OHCA:ti,ab,kw OR CPR:ti,ab,kw OR 'cardiopulmonary resuscitation':ti,ab,kw OR ‘sudden cardiac death’/exp OR ‘out of hospital cardiac arrest’/exp OR 'resuscitation'/exp OR ‘heart arrest’/exp)  AND  (covid-19:ti,ab,kw OR coronavirus disease-19':ti,ab,kw OR coronavirus:ti,ab,kw OR ORSARS-CoV-2:ti,ab,kw OR ‘SARS coronavirus’/exp OR ‘coronavirus disease 2019’/exp) |
| Cochrane library | ((cardiac arrest):ti,ab,kw OR (sudden cardiac arrest):ti,ab,kw OR (sudden cardiac death):ti,ab,kw OR (out of hospital cardiac arrest):ti,ab,kw OR (out-of-hospital cardiac arrest):ti,ab,kw OR (OOHCA):ti,ab,kw OR (OHCA):ti,ab,kw OR (CPR):ti,ab,kw OR (cardiopulmonary resuscitation):ti,ab,kw OR MeSH descriptor: [Death, Sudden, Cardiac] explode all trees OR MeSH descriptor: [Out-of-Hospital Cardiac Arrest] explode all trees OR MeSH descriptor: [Cardiopulmonary Resuscitation] explode all trees OR MeSH descriptor: [Heart arrest] explode all trees)  AND  ((covid-19):ti,ab,kw OR (coronavirus disease-19):ti,ab,kw OR (coronavirus):ti,ab,kw OR (SARS-CoV-2):ti,ab,kw OR MeSH descriptor: [SARS-CoV-2] explode all trees OR MeSH descriptor: [COVID-19] explode all trees) |
| medRxiv | Out of hospital cardiac arrest AND COVID-19 |
| bioRxiv | Out of hospital cardiac arrest AND COVID-19 |

**Supplementary Table 3.** List of 48 included studies in meta-analysis

| No | Study |
| --- | --- |
| 1 | Ahn JY, Ryoo HW, Cho JW, Kim JH, Lee SH, Jang TC. Impact of the covid-19 outbreak on adult out-of-hospital cardiac arrest outcomes in daegu, south korea: An observational study. Clinical and Experimental Emergency Medicine. 2021;8(2):137-44. |
| 2 | Baert V, Jaeger D, Hubert H, Lascarrou JB, Debaty G, Chouihed T, et al. Assessment of changes in cardiopulmonary resuscitation practices and outcomes on 1005 victims of out-of-hospital cardiac arrest during the COVID-19 outbreak: registry-based study. Scandinavian journal of trauma, resuscitation and emergency medicine. 2020;28(1):119. |
| 3 | Baldi E, Auricchio A, Klersy C, Burkart R, Benvenuti C, Vanetta C, et al. Out-of-hospital cardiac arrests and mortality in Swiss Cantons with high and low COVID-19 incidence: A nationwide analysis. Resusc Plus. 2021;6:100105. |
| 4 | Baldi E, Caputo ML, Auricchio A, Vanetta C, Cresta R, Benvenuti C. A quantitative assessment of the contribution of “citizen First Responder” in the adult out-of-hospital chain of survival during COVID-19 pandemic. Resuscitation. 2021;166:41-2. |
| 5 | Baldi E, Sechi GM, Mare C, Canevari F, Brancaglione A, Primi R, et al. COVID-19 kills at home: The close relationship between the epidemic and the increase of out-of-hospital cardiac arrests. European Heart Journal. 2020;41(32):3045-54. |
| 6 | Baldi E, Sechi GM, Mare C, Canevari F, Brancaglione A, Primi R, et al. Treatment of out-of-hospital cardiac arrest in the COVID-19 era: A 100 days experience from the Lombardy region. PLoS ONE. 2020;15(10). |
| 7 | Ball J, Nehme Z, Bernard S, Stub D, Stephenson M, Smith K. Collateral damage: Hidden impact of the COVID-19 pandemic on the out-of-hospital cardiac arrest system-of-care. Resuscitation. 2020;156:157-63. |
| 8 | Biskupski P, Heraud SO, Khalil M, Horoub A, Maqsood M, Ong K, et al. Out-of-hospital Cardiac Arrest Before and During the COVID-19 Pandemic in the South Bronx. medRxiv. 2022. |
| 9 | Burns TA, Touzeau C, Kaufman BT, Butsch AL, Vesselinov R, Stone RM. Decreases in out of hospital cardiac arrest (OHCA) outcome metrics persist when known COVID patients are excluded from analysis. American Journal of Emergency Medicine. 2022;51:64-8. |
| 10 | Chan PS, Girotra S, Tang Y, Al-Araji R, Nallamothu BK, McNally B. Outcomes for Out-of-Hospital Cardiac Arrest in the United States during the Coronavirus Disease 2019 Pandemic. JAMA Cardiology. 2021;6(3):296-303. |
| 11 | Chavez S, Huebinger R, Chan HK, Gill J, White L, Mendez D, et al. The impact of COVID-19 on incidence and outcomes from out-of-hospital cardiac arrest (OHCA) in Texas. The American Journal of Emergency Medicine. 2022;57:1-5. |
| 12 | Cho JW, Jung H, Lee MJ, Lee SH, Lee SH, Mun YH, et al. Preparedness of personal protective equipment and implementation of new CPR strategies for patients with out-of-hospital cardiac arrest in the COVID-19 era. Resusc Plus. 2020;3:100015. |
| 13 | Chung H, Namgung M, Lee DH, Choi YH, Bae SJ. Effect of delayed transport on clinical outcomes among patients with cardiac arrest during the coronavirus disease 2019 pandemic. Australasian emergency care. 2021. |
| 14 | Coute RA, Nathanson BH, Kurz MC, Mader TJ, Group CS. Estimating the impact of the COVID‐19 pandemic on out‐of‐hospital cardiac arrest burden of disease in the United States. Journal of the American College of Emergency Physicians Open. 2022;3(5):e12811. |
| 15 | Damjanovic D, Pooth J-S, Steger R, Boeker M, Steger M, Ganter J, et al. Observational study on implications of the COVID-19-pandemic for cardiopulmonary resuscitation in out-of-hospital cardiac arrest: qualitative and quantitative insights from a model region in Germany. BMC Emergency Medicine. 2022;22(1):1-11. |
| 16 | de Koning ER, Boogers MJ, Bosch J, de Visser M, Schalij MJ, Beeres SLMA. Emergency medical services evaluations for chest pain during first COVID-19 lockdown in Hollands-Midden, the Netherlands. Netherlands Heart Journal. 2021;29(4):224-9. |
| 17 | Elmer J, Okubo M, Guyette FX, Martin-Gill C. Indirect effects of COVID-19 on OHCA in a low prevalence region. Resuscitation. 2020;156:282-3. |
| 18 | Fothergill RT, Smith AL, Wrigley F, Perkins GD. Out-of-Hospital Cardiac Arrest in London during the COVID-19 pandemic. Resusc Plus. 2021;5:100066. |
| 19 | Glober NK, Supples M, Faris G, Arkins T, Christopher S, Fulks T, et al. Out-of-hospital cardiac arrest volumes and characteristics during the COVID-19 pandemic. American Journal of Emergency Medicine. 2021;48:191-7. |
| 20 | Grübl T, Plöger B, Sassen MC, Jerrentrup A, Schieffer B, Betz S. Out-of-hospital cardiac arrest during lockdown: Effects of the infection prevention measures during the first wave of SARS-CoV-2 pandemic. Notfall und Rettungsmedizin. 2021. |
| 21 | Hosomi S, Zha L, Kiyohara K, Kitamura T, Irisawa T, Ogura H, et al. Survival following an out‐of‐hospital cardiac arrest in Japan in 2020 versus 2019 according to the cause. Acute Medicine & Surgery. 2022;9(1):e777. |
| 22 | Kandori K, Okada Y, Ishii W, Narumiya H, Iizuka R. Evaluation of a revised resuscitation protocol for out-of-hospital cardiac arrest patients due to COVID-19 safety protocols: a single-center retrospective study in Japan. Scientific reports. 2021;11(1):12985. |
| 23 | Lai PH, Prezant DJ, Lancet EA, Weiden MD, Webber MP, Zeig-Owens R, et al. Characteristics Associated with Out-of-Hospital Cardiac Arrests and Resuscitations during the Novel Coronavirus Disease 2019 Pandemic in New York City. JAMA Cardiology. 2020;5(10):1154-63. |
| 24 | Lee N-J, Yang J-C, Moon J-d. Collateral damage of emergency medical services due to COVID-19. The Korean Journal of Emergency Medical Services. 2021;25(3):189-200. |
| 25 | Lim D, Park SY, Choi B, Kim SH, Ryu JH, Kim YH, et al. The Comparison of Emergency Medical Service Responses to and Outcomes of Out-of-hospital Cardiac Arrest before and during the COVID-19 Pandemic in an Area of Korea. Journal of Korean medical science. 2021;36(36):e255. |
| 26 | Lim KT, Ahn KO, Park JH, Park CH, Lim J, Lee K. Bystander cardiopulmonary resuscitation in public locations before and after the coronavirus disease 2019 pandemic in the Republic of Korea. The American Journal of Emergency Medicine. 2022;56:271. |
| 27 | Lim SL, Kumar L, Saffari SE, Shahidah N, Al-Araji R, Ng QX, et al. Management of Out-of-Hospital Cardiac Arrest during COVID-19: A Tale of Two Cities. Journal of clinical medicine. 2022;11(17):5177. |
| 28 | Lim SL, Shahidah N, Saffari SE, Ng QX, Ho AFW, Leong BSH, et al. Impact of covid‐19 on out‐of‐hospital cardiac arrest in Singapore. International Journal of Environmental Research and Public Health. 2021;18(7). |
| 29 | Marijon E, Karam N, Jost D, Perrot D, Frattini B, Derkenne C, et al. Out-of-hospital cardiac arrest during the COVID-19 pandemic in Paris, France: a population-based, observational study. The Lancet Public Health. 2020;5(8):e437-e43. |
| 30 | Mathew S, Harrison N, Chalek AD, Gorelick D, Brennan E, Wise S, et al. Effects of the COVID-19 pandemic on out-of-hospital cardiac arrest care in Detroit. American Journal of Emergency Medicine. 2021;46:90-6. |
| 31 | Navalpotro-Pascual J-M, Monge-Martín D, González-León M-J, Neria F, Peinado-Vallejo F, Alonso-Blas C, et al. Impact of Different Waves of COVID-19 on Emergency Medical Services and Out-of-hospital Cardiopulmonary Arrest in Madrid, Spain. Research Square. 2021. |
| 32 | Navalpotro-Pascual JM, Pérez CF, Vallejo FAP, Moya AC, Cuesta YM, Isabel BM, et al. Caseload and cardiopulmonary arrest management by an out-of-hospital emergency service during the covid-19 pandemic. Emergencias. 2021;33(2):100-6. |
| 33 | Ng QX, Lee EZH, Tay JAM, Arulanandam S. Impact of COVID-19 ‘circuit-breaker’ measures on emergency medical services utilisation and out-of-hospital cardiac arrest outcomes in Singapore. EMA - Emergency Medicine Australasia. 2021;33(1):149-51. |
| 34 | Nickles AV, Oostema A, Allen J, O'Brien SL, Demel SL, Reeves MJ. Comparison of Out-of-Hospital Cardiac Arrests and Fatalities in the Metro Detroit Area during the COVID-19 Pandemic with Previous-Year Events. JAMA Network Open. 2021;4(1). |
| 35 | Nishiyama C, Kiyohara K, Iwami T, Hayashida S, Kiguchi T, Matsuyama T, et al. Influence of COVID-19 pandemic on bystander interventions, emergency medical service activities, and patient outcomes in out-of-hospital cardiac arrest in Osaka City, Japan. Resusc Plus. 2021;5:100088. |
| 36 | Nishiyama C, Kiyohara K, Kitamura T, Hayashida S, Maeda T, Kiguchi T, et al. Impact of the COVID-19 Pandemic on Prehospital Intervention and Survival of Patients With Out-of-Hospital Cardiac Arrest in Osaka City, Japan. Circulation Journal. 2022:CJ-22-0040. |
| 37 | Ortiz F, Fernández del Valle P, Knox EC, Jiménez Fábrega X, Navalpotro Pascual JM, Mateo Rodríguez I, et al. Influence of the Covid-19 pandemic on out-of-hospital cardiac arrest. A Spanish nationwide prospective cohort study. Resuscitation. 2020;157:230-40. |
| 38 | Paoli A, Brischigliaro L, Scquizzato T, Favaretto A, Spagna A. Out-of-hospital cardiac arrest during the COVID-19 pandemic in the Province of Padua, Northeast Italy. Resuscitation. 2020;154:47-9. |
| 39 | Phattharapornjaroen P, Nimnuan W, Sanguanwit P, Atiksawedparit P, Phontabtim M, Mankong Y. Characteristics and outcomes of out-of-hospital cardiac arrest patients before and during the COVID-19 pandemic in Thailand. International journal of emergency medicine. 2022;15(1):1-11. |
| 40 | Ristau P, Wnent J, Gräsner J-T, Fischer M, Bohn A, Bein B, et al. Impact of COVID-19 on out-of-hospital cardiac arrest: A registry-based cohort-study from the German Resuscitation Registry. Plos one. 2022;17(9):e0274314. |
| 41 | Riyapan S, Chantanakomes J, Roongsaenthong P, Tianwibool P, Wittayachamnankul B, Supasaovapak J, et al. Impact of the COVID-19 outbreak on out-of-hospital cardiac arrest management and outcomes in a low-resource emergency medical service system: a perspective from Thailand. Int J Emerg Med. 2022;15(1):26. |
| 42 | Sayre MR, Barnard LM, Counts CR, Drucker CJ, Kudenchuk PJ, Rea TD, et al. Prevalence of COVID-19 in Out-of-Hospital Cardiac Arrest: Implications for Bystander Cardiopulmonary Resuscitation. Circulation. 2020;142(5):507-9. |
| 43 | Semeraro F, Gamberini L, Tartaglione M, Iarussi B, Descovich C, Picoco C, et al. Out-of-hospital cardiac arrest during the COVID-19 era in Bologna: System response to preserve performances. Resuscitation. 2020;157:1-2. |
| 44 | Sultanian P, Lundgren P, Strömsöe A, Aune S, Bergström G, Hagberg E, et al. Cardiac arrest in COVID-19: Characteristics and outcomes of in- And out-of-hospital cardiac arrest. A report from the Swedish Registry for Cardiopulmonary Resuscitation. European Heart Journal. 2021;42(11):1094-106. |
| 45 | Sun C, Dyer S, Salvia J, Segal L, Levi R. Worse cardiac arrest outcomes during the covid-19 pandemic in boston can be attributed to patient reluctance to seek care. Health Affairs. 2021;40(6):886-95. |
| 46 | Talikowska M, Ball S, Tohira H, Bailey P, Rose D, Brink D, et al. No apparent effect of the COVID-19 pandemic on out-of-hospital cardiac arrest incidence and outcome in Western Australia. Resusc Plus. 2021;8:100183. |
| 47 | Uy-Evanado A, Chugh HS, Sargsyan A, Nakamura K, Mariani R, Hadduck K, et al. Out-of-Hospital Cardiac Arrest Response and Outcomes During the COVID-19 Pandemic. JACC: Clinical Electrophysiology. 2021;7(1):6-11. |
| 48 | Yu JH, Liu CY, Chen WK, Yu SH, Huang FW, Yang MT, et al. Impact of the COVID-19 pandemic on emergency medical service response to out-of-hospital cardiac arrests in Taiwan: A retrospective observational study. Emergency Medicine Journal. 2021;38(9):679-84. |

**Supplementary Figure 1.** Study period during COVID-19 pandemic of each study


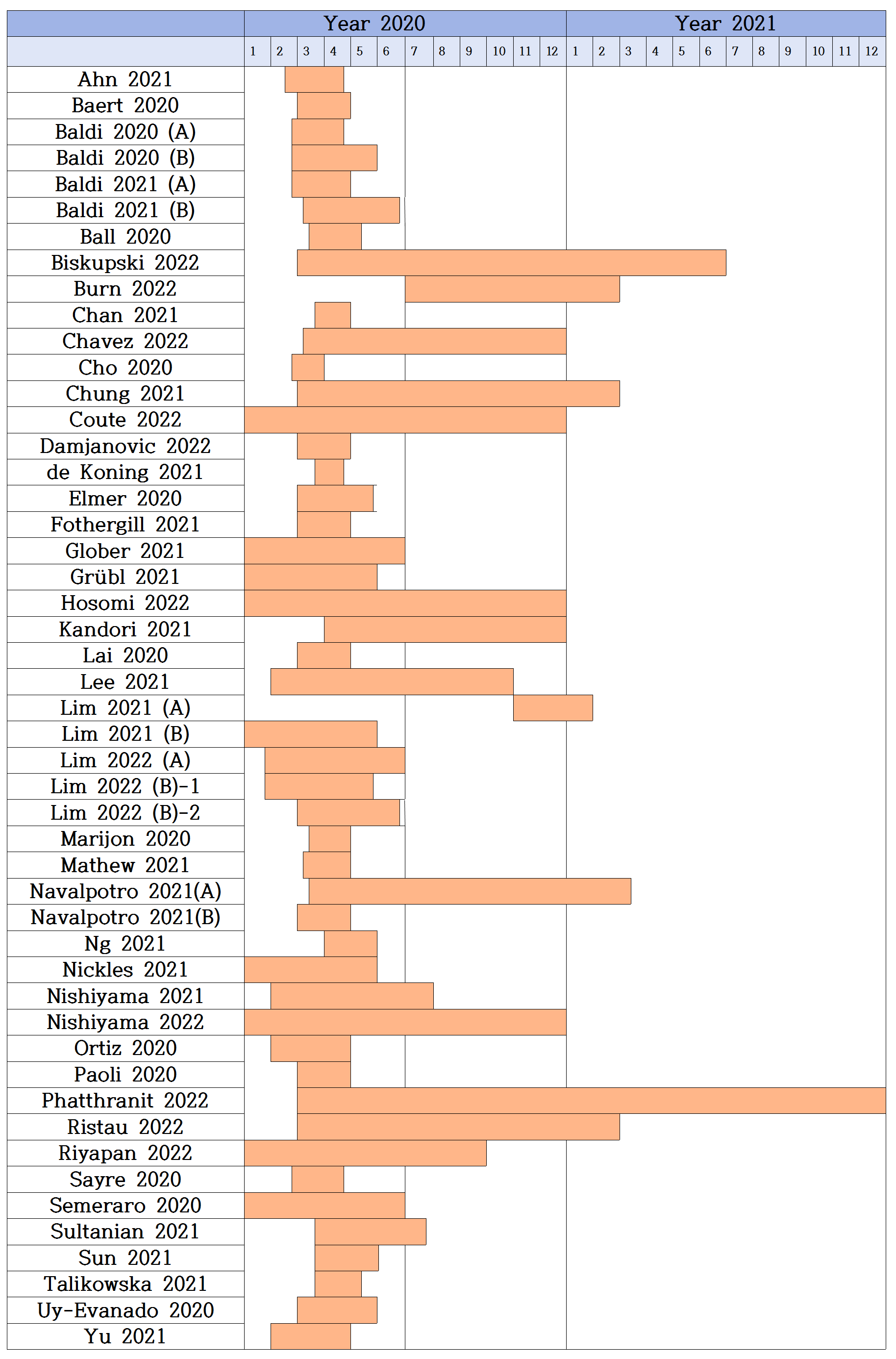


**Supplementary Figure** **2.** Plot of the distribution for each item from included studies.


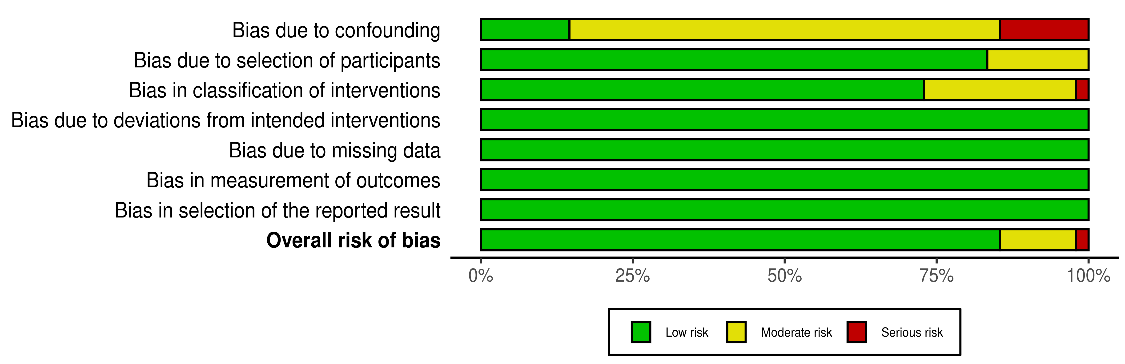


**Supplementary Figure 3.** A summary table for assessment of each study.


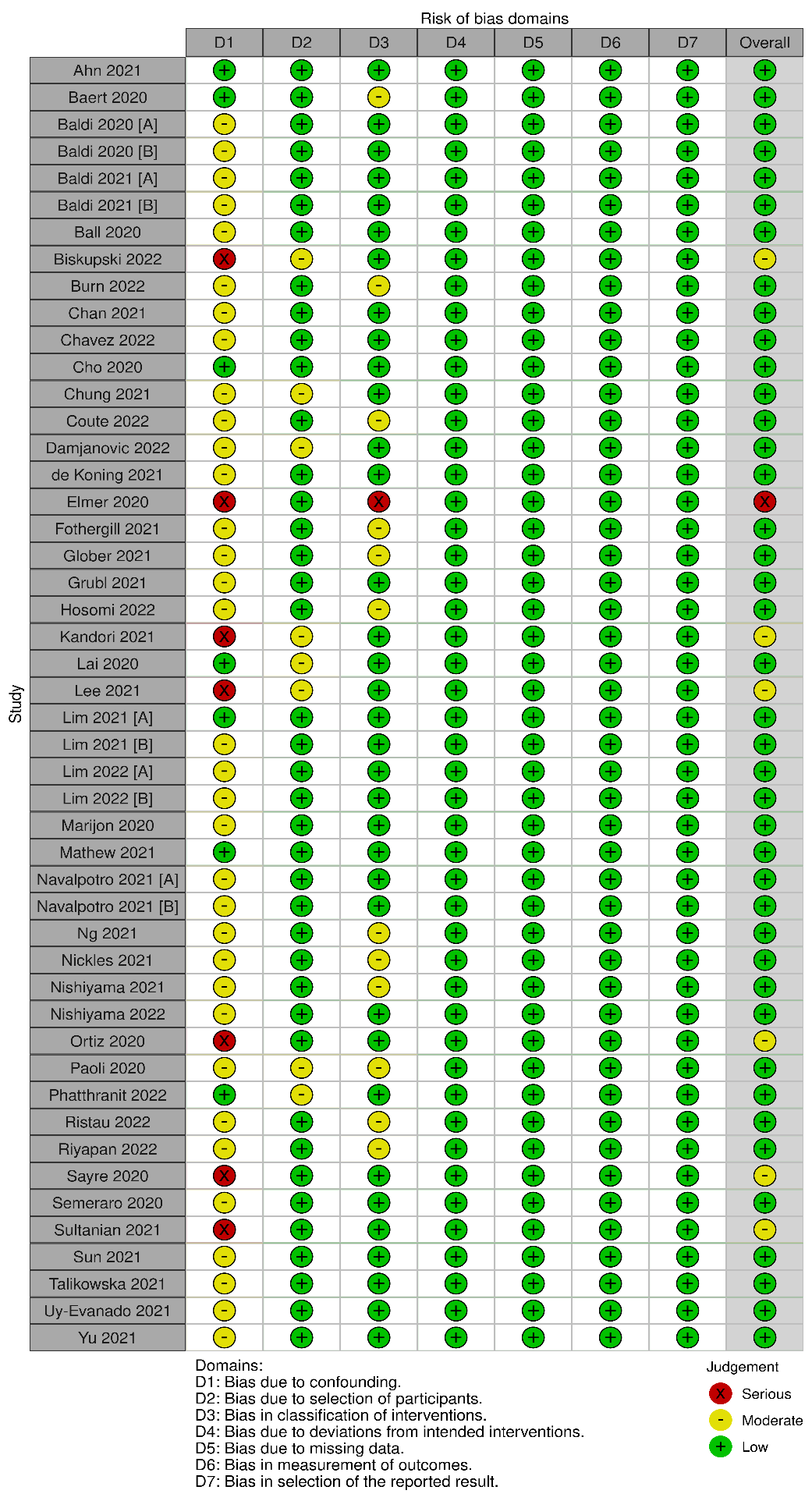


**Supplementary Figure 4.** Forest plot for target temperature management during the pandemic comparing to before the pandemic.

**
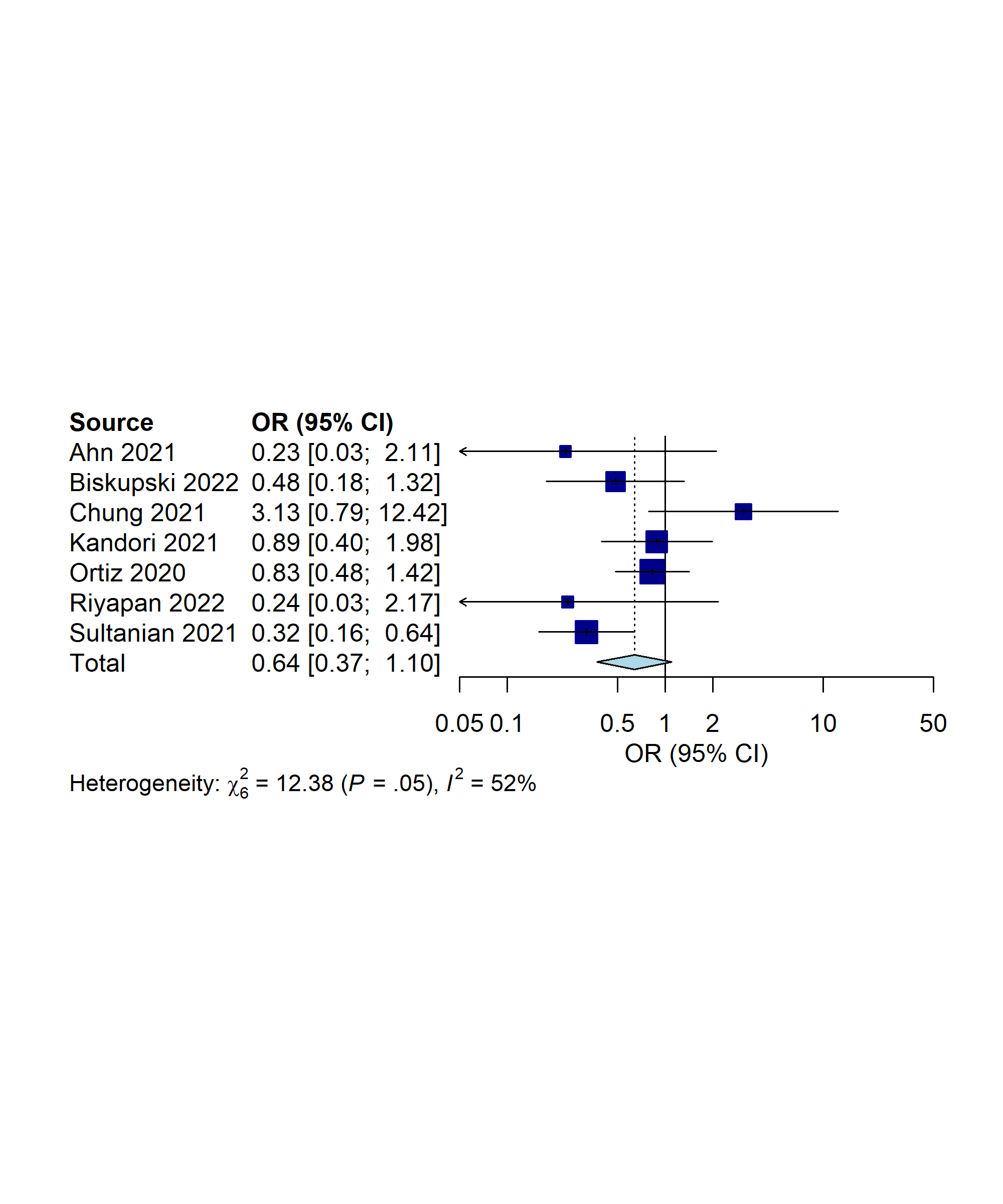
**

**Supplementary Figure 5.** Forest plot depicting outcome during the COVID-19 pandemic compared with that before the pandemic, subgroup analysis according to study period of pandemic. (a) Survival to hospital discharge, (b) Return of spontaneous circulation, (c) Survival to hospital admission, (d) 30-day survival, and (e) Favorable neurological outcome.


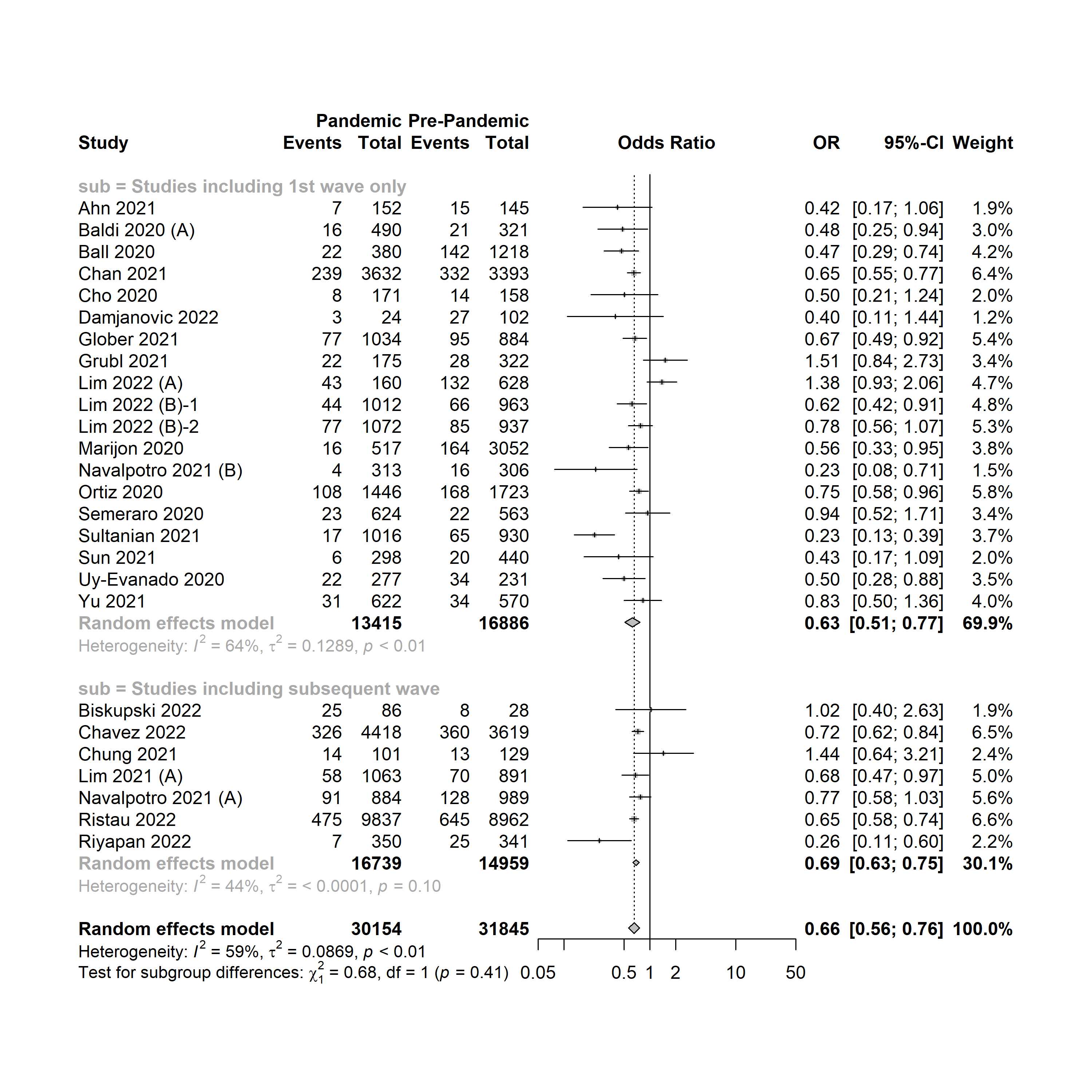


(a) Survival to hospital discharge


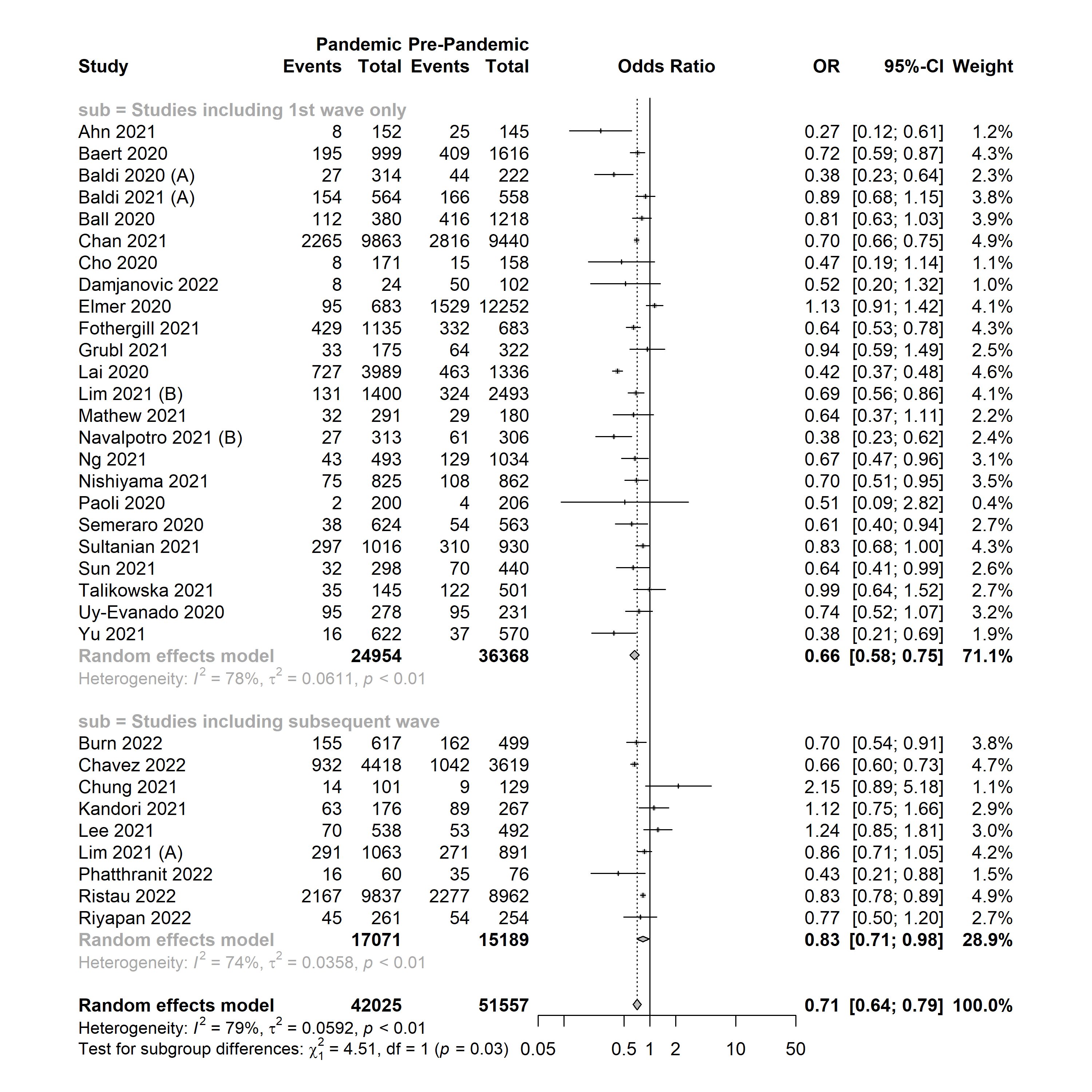


(b) Return of spontaneous circulation


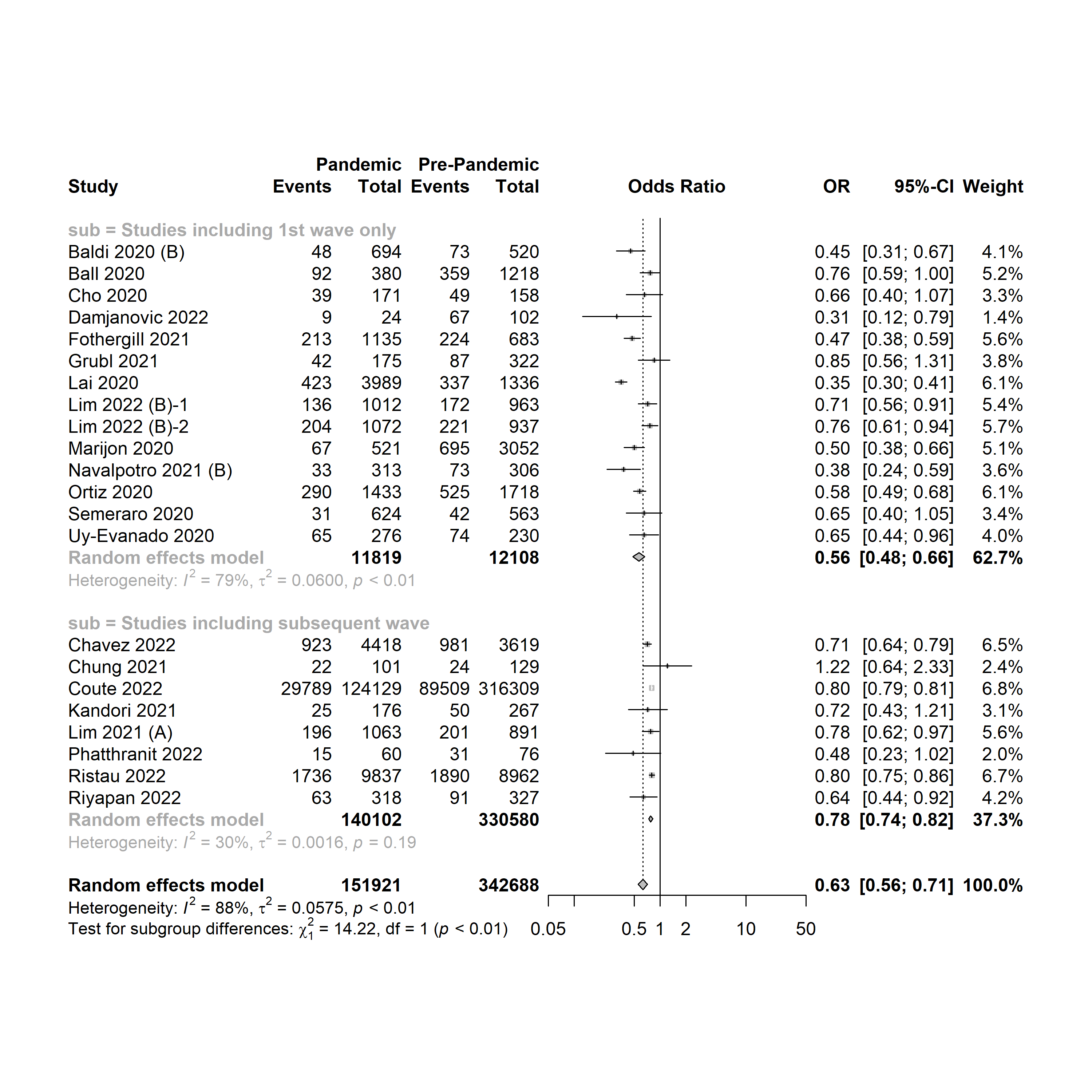


(c) Survival to hospital admission


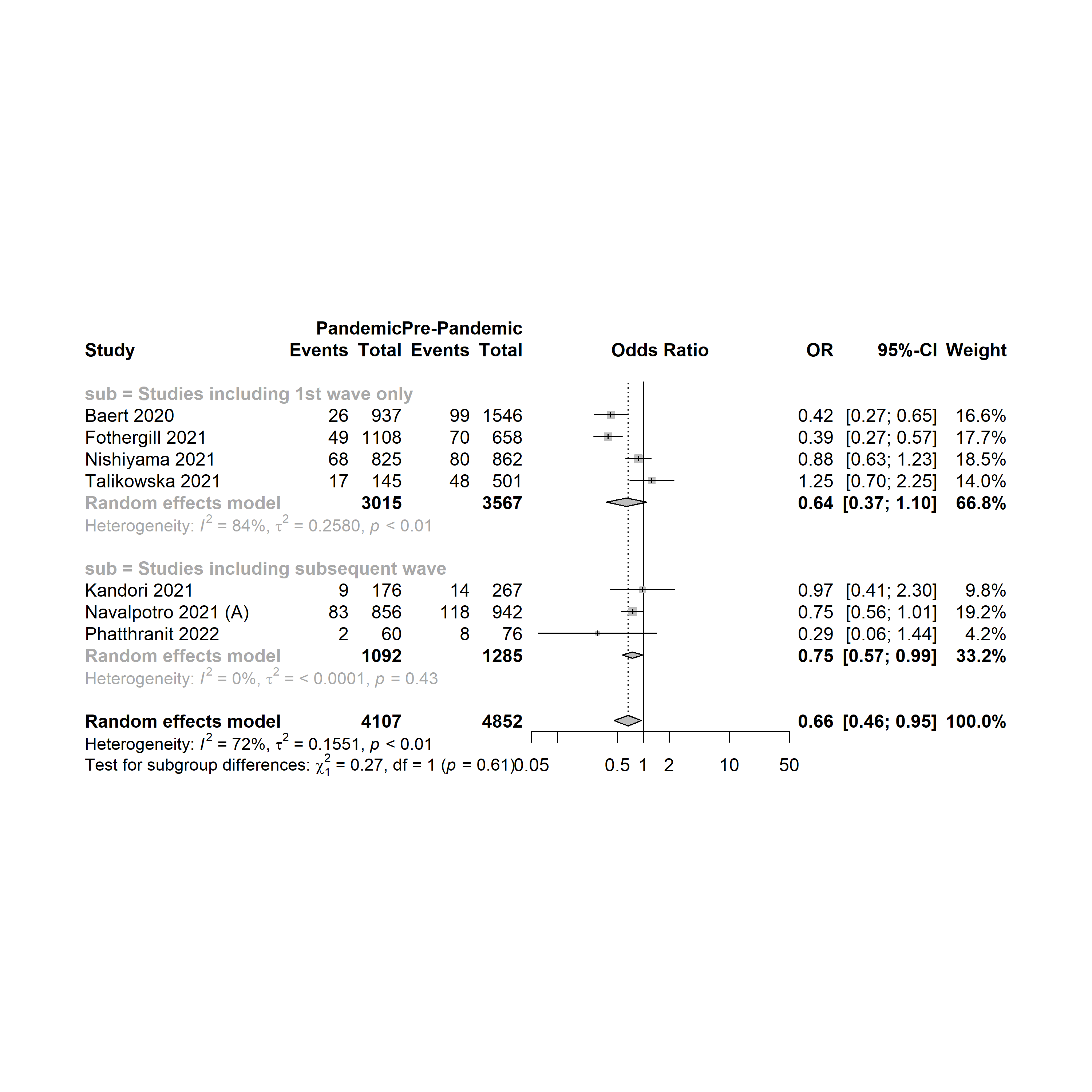


(d) 30-day survival


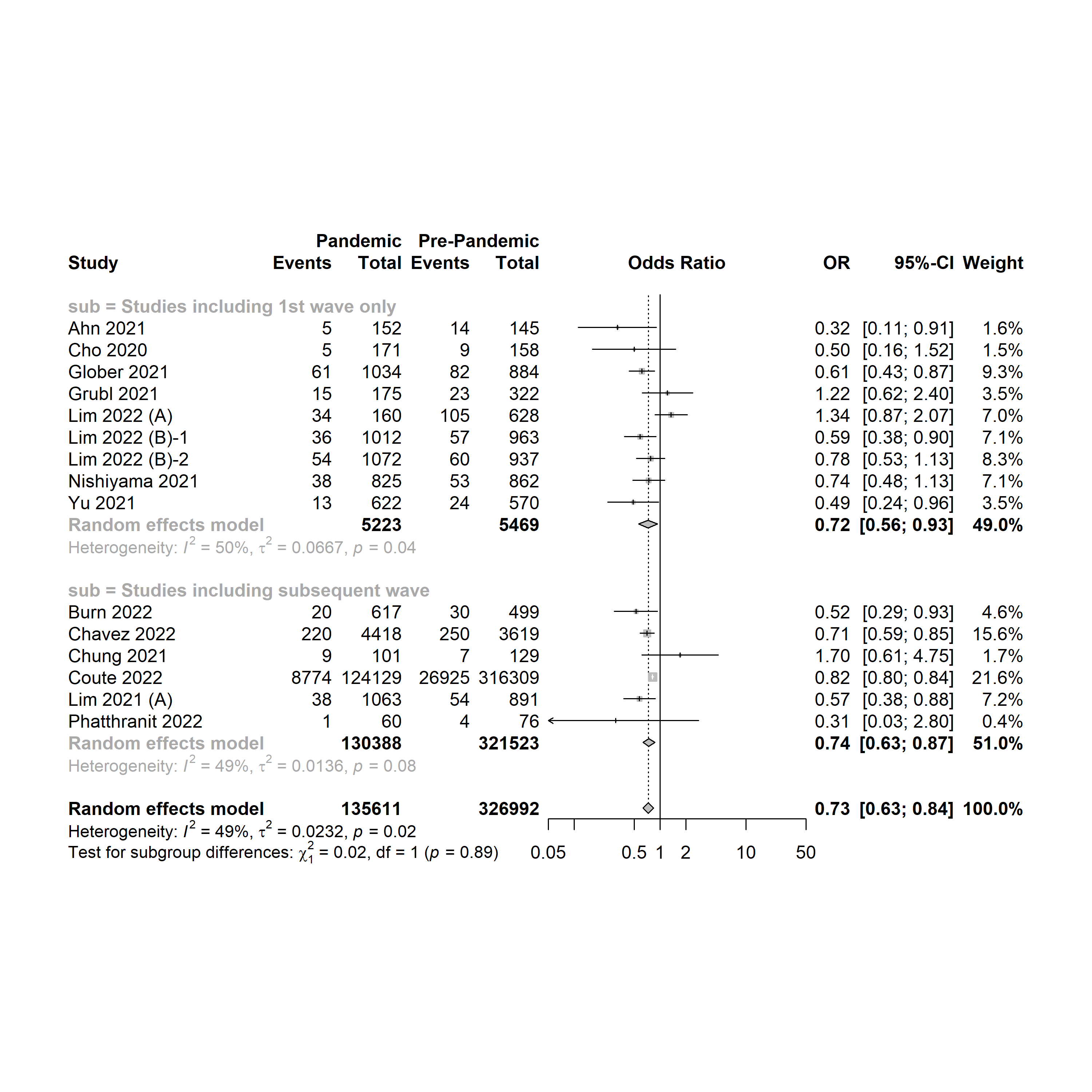


(e) Favorable neurological outcome

**Supplementary Figure 6.** Forest plot of epidemiologic factors during the COVID-19 pandemic compared with that before the pandemic, and subgroup analysis according to the study period during the pandemic. (a) Cardiac arrest at home, (b) Use of automated external defibrillators, (c) Shockable rhythm, (d) Unwitnessed cardiac arrest, and (e) Bystander CPR.


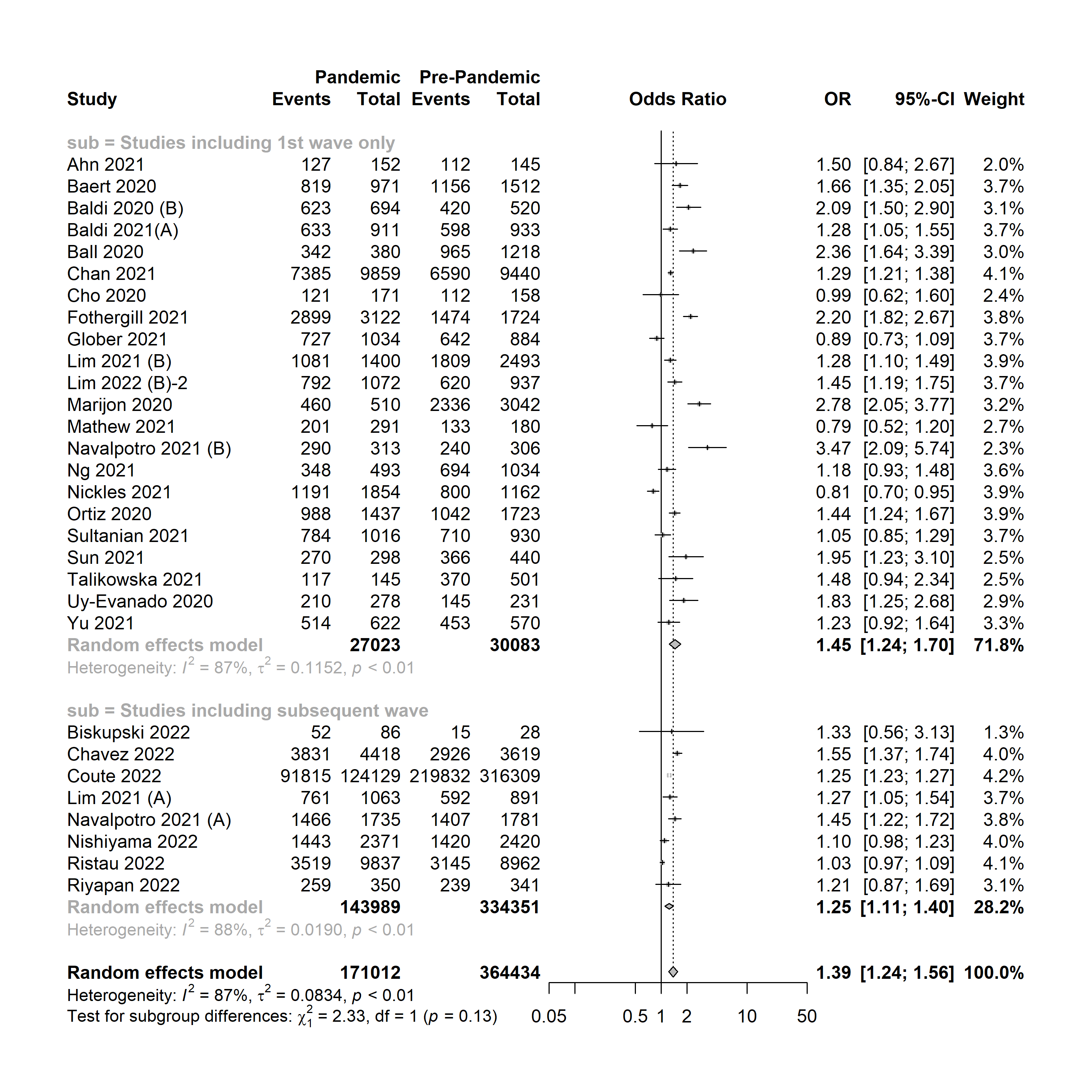


(a) Arrest at home


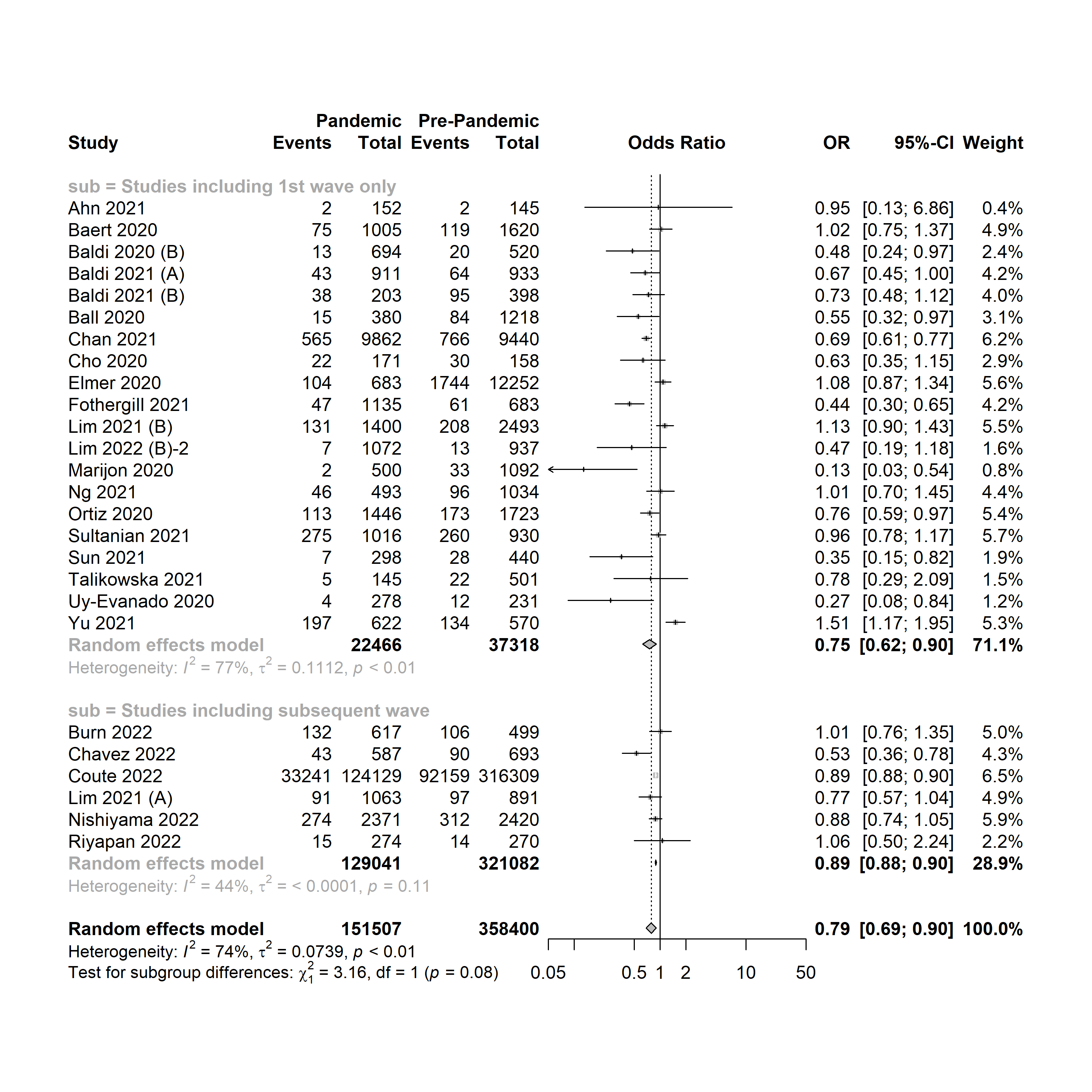


(b) Use of automated external defibrillators


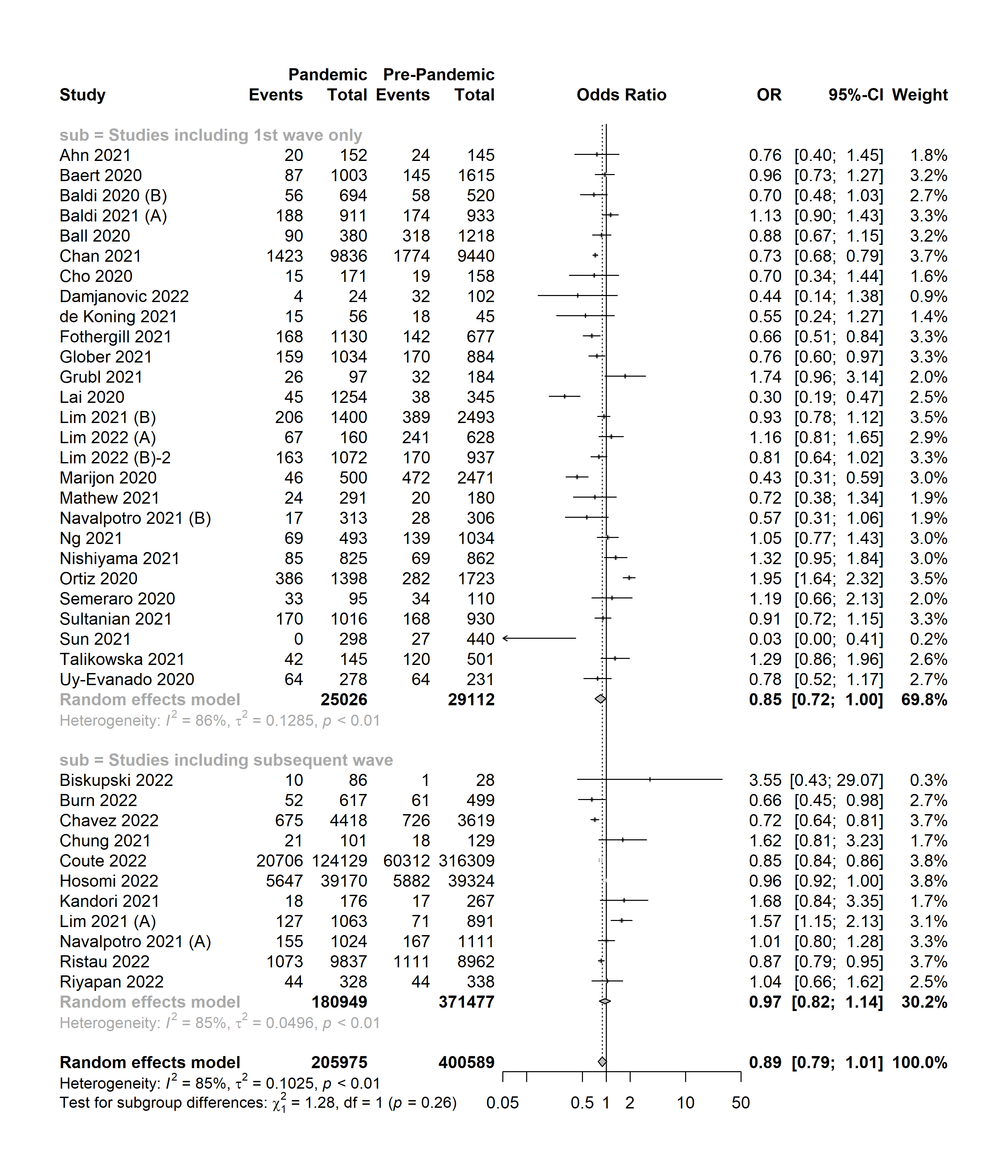


(c) Shockable rhythm


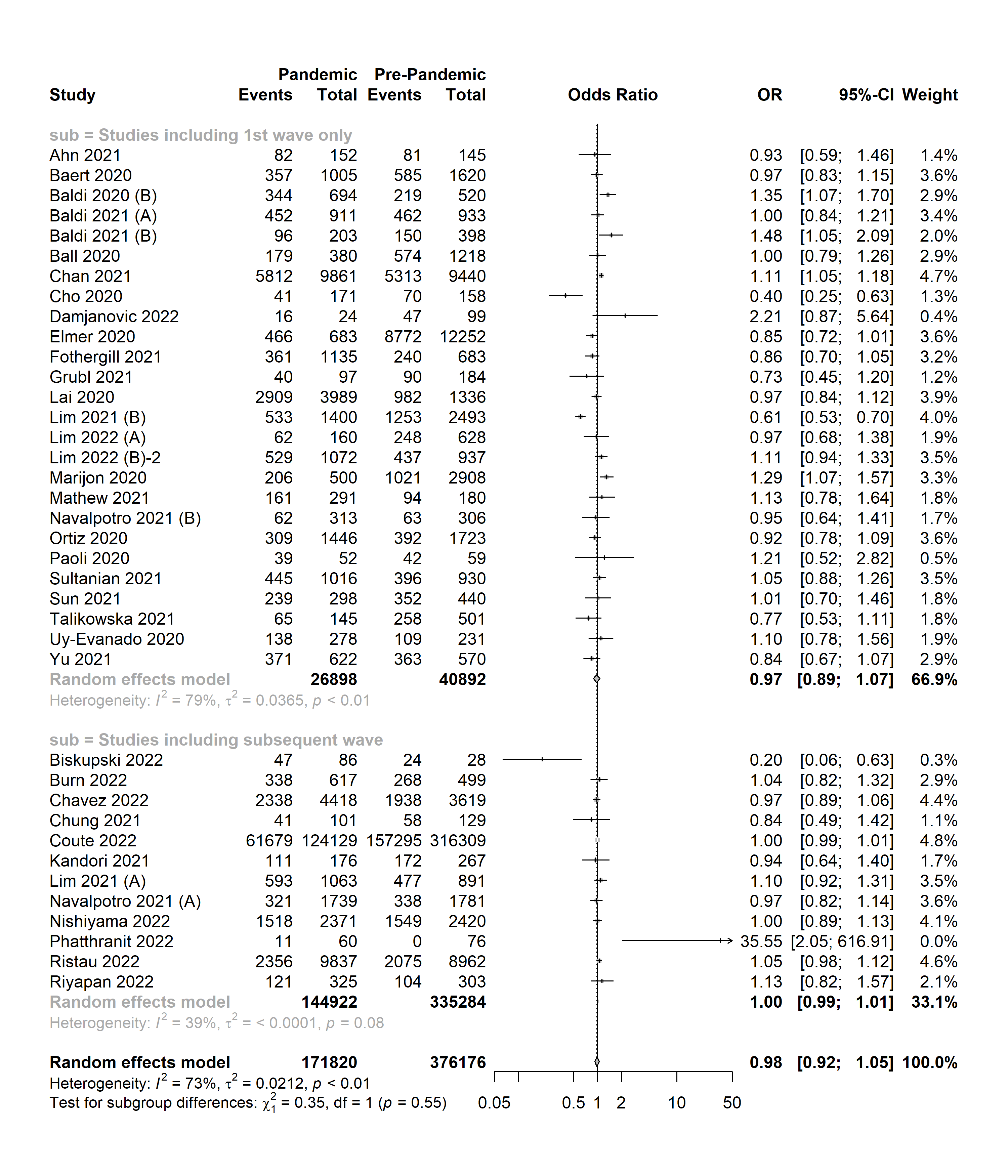


(d) Unwitnessed cardiac arrest


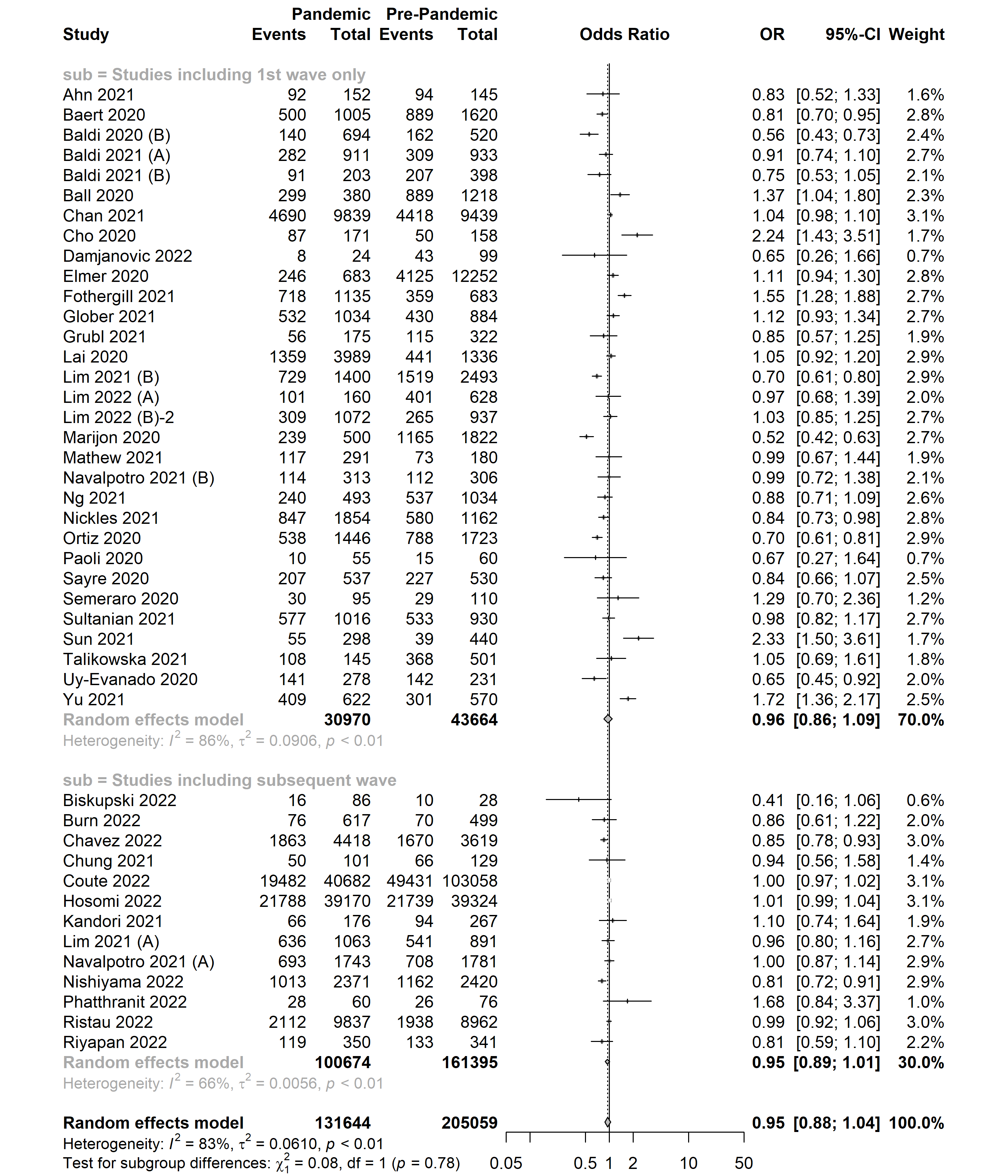


(e) Bystander CPR

**Supplementary Figure 7.** Forest plot for prehospital factors during the COVID-19 pandemic compared with that before the pandemic, and subgroup analysis according to study period during the pandemic. (a) EMS response time, (b) EMS transport time, (c) Supraglottic airway device, (d) Endotracheal intubation, (e) Mechanical CPR, and (f) in S3 Text. Prehospital return of spontaneous circulation.


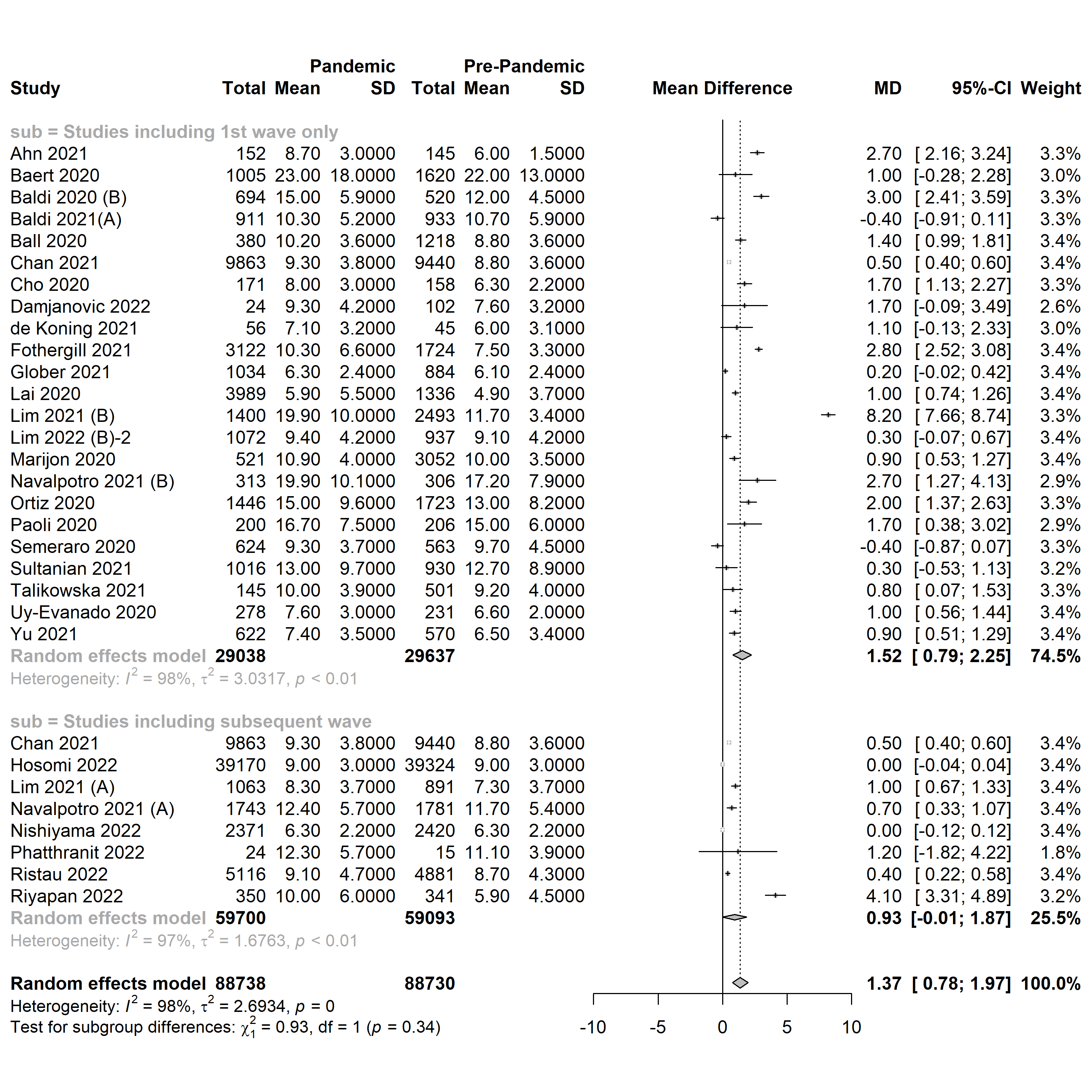


(a) EMS response time


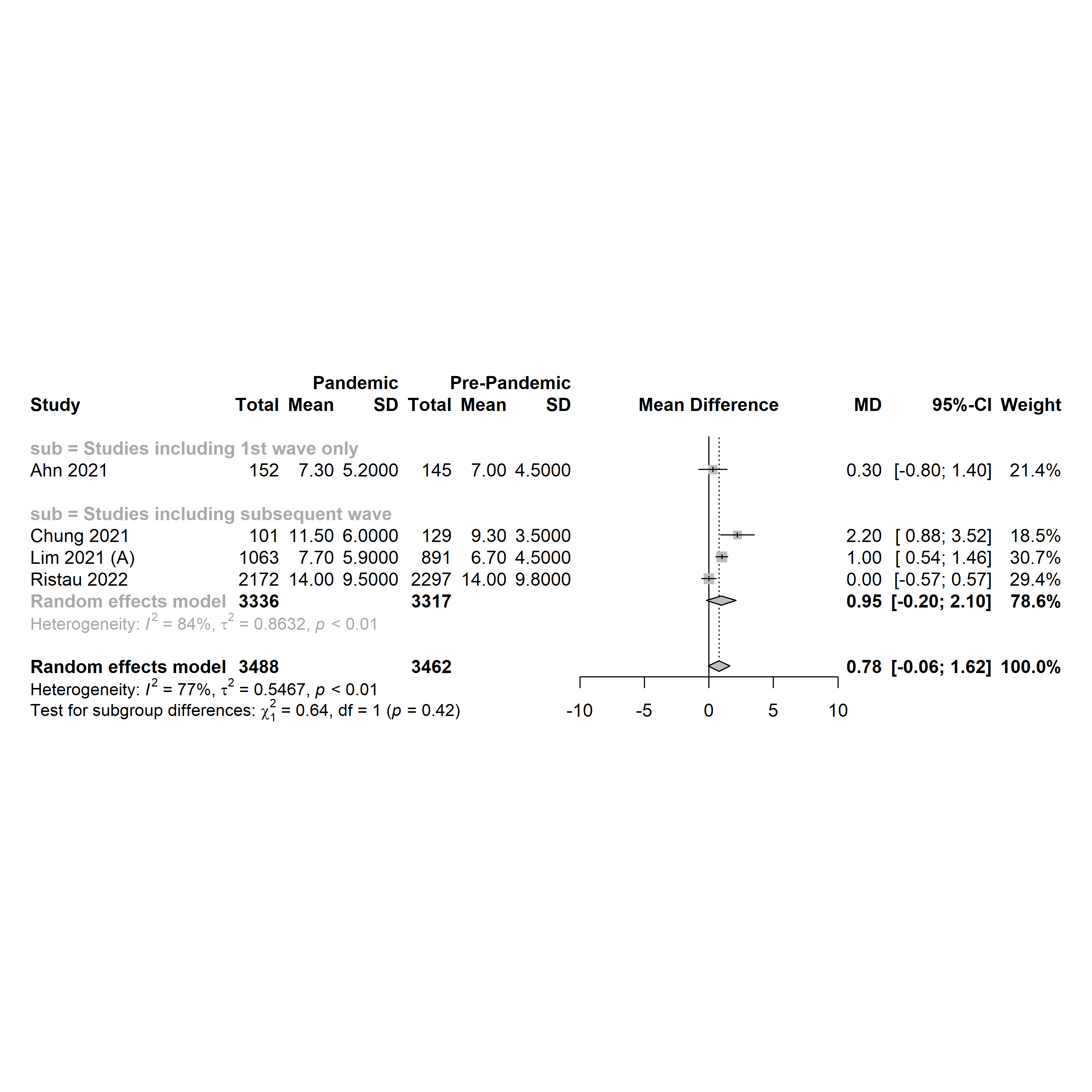


(b) EMS transport time


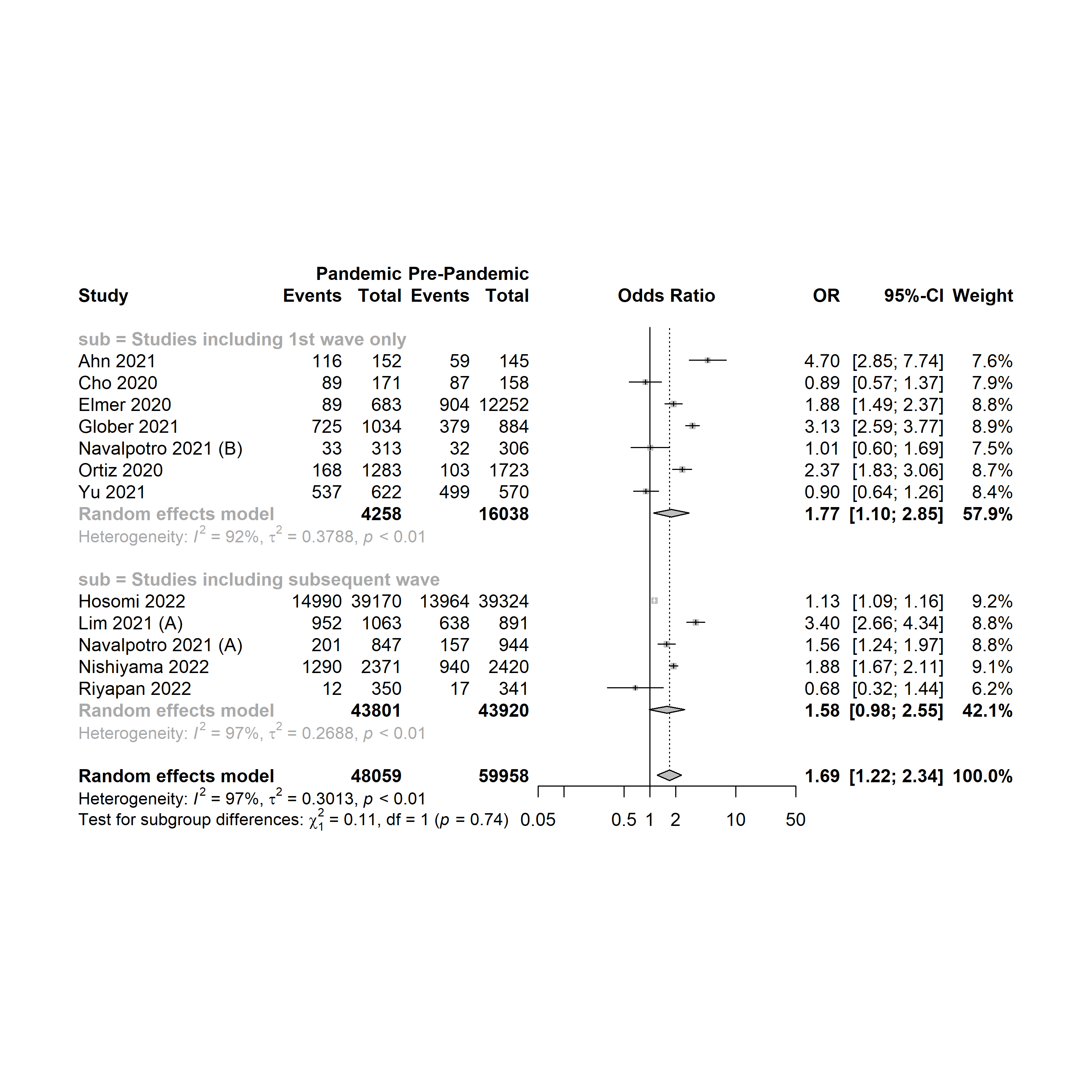


(c) Supraglottic airway device


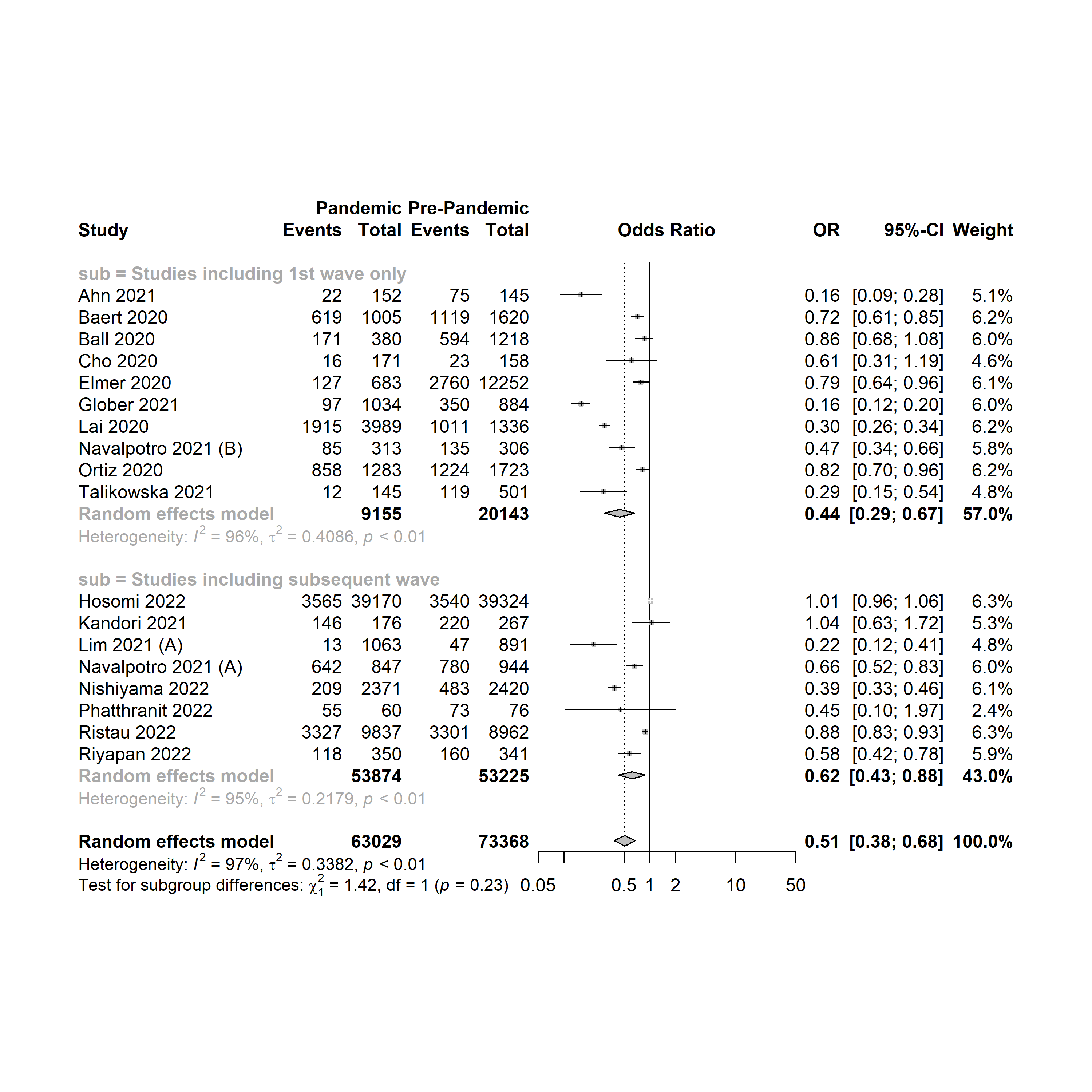


(d) Endotracheal intubation


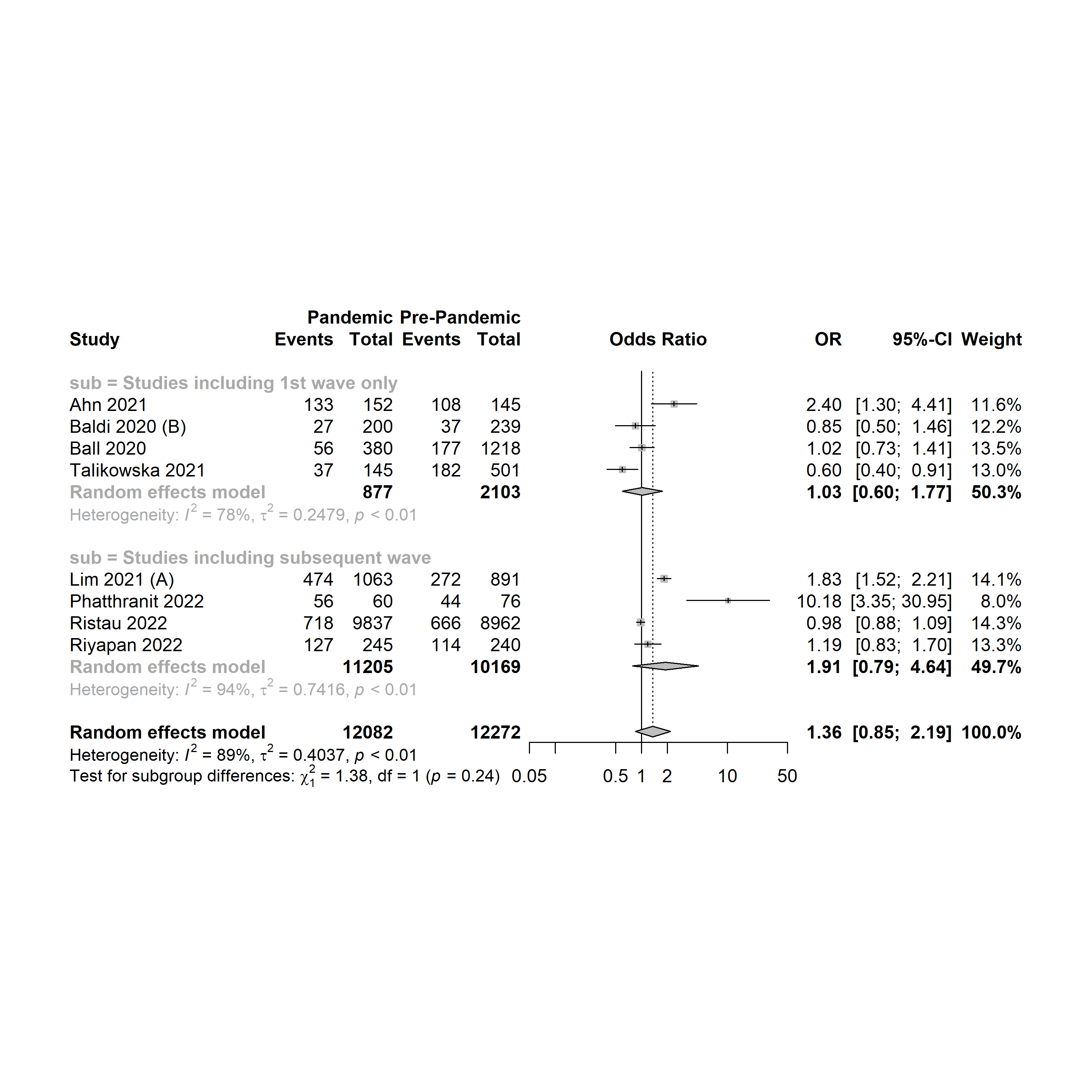


(e) Mechanical CPR


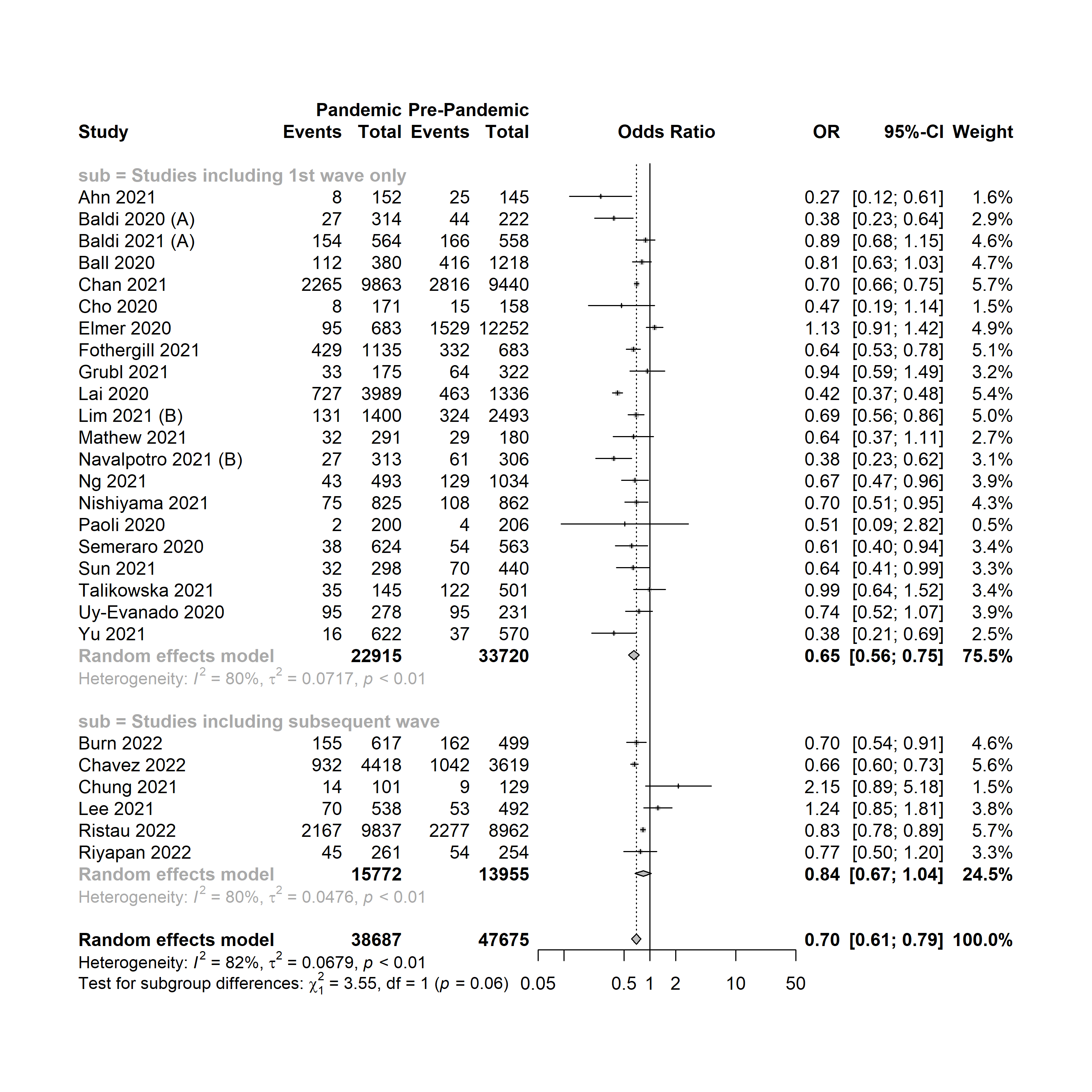


(f) Prehospital return of spontaneous circulation

**Supplementary Figure 8.** Forest plot for target temperature management during the COVID-19 pandemic compared with that before the pandemic, and subgroup analysis according to the study period of the pandemic.


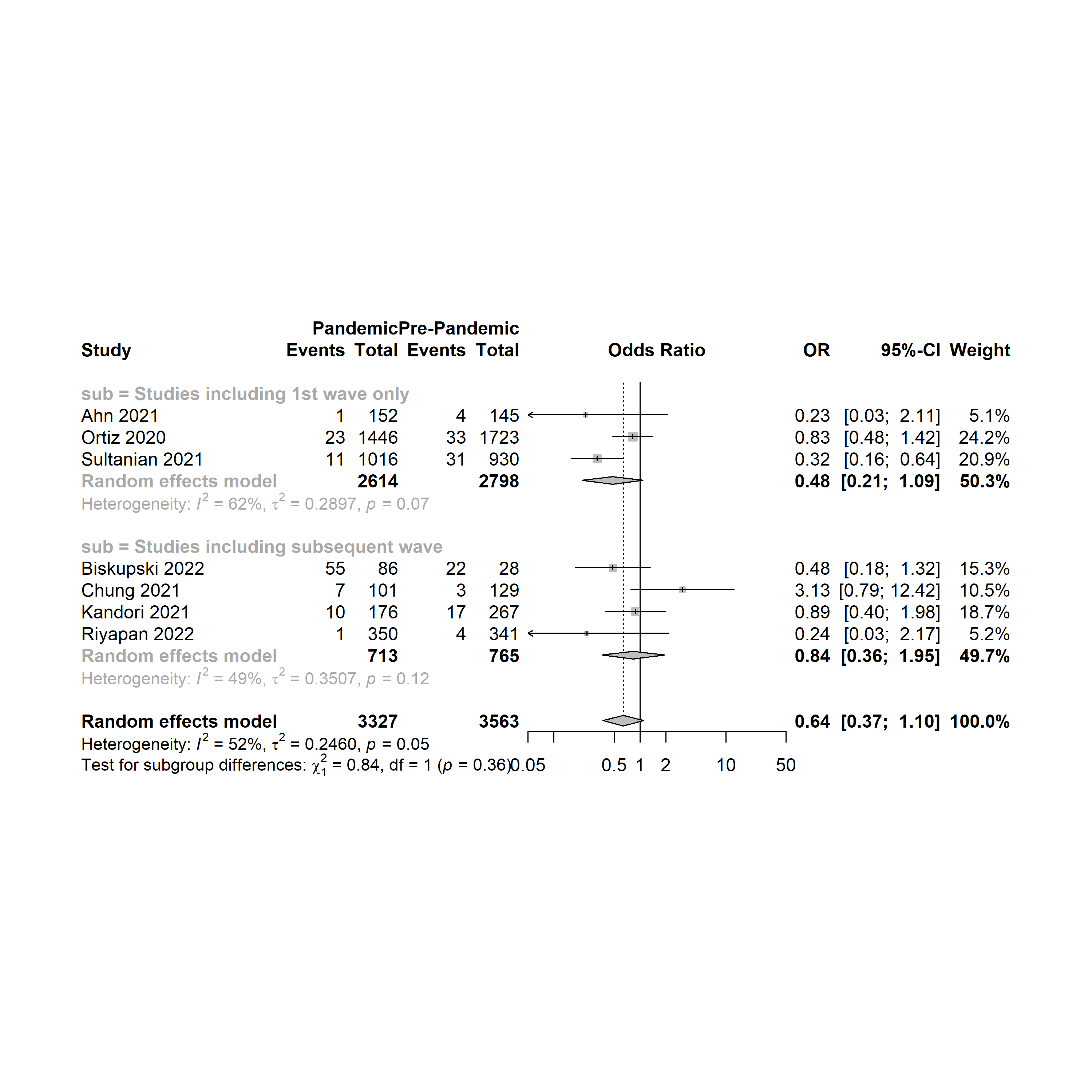


**Supplementary Figure 9.** Forest plot for outcomes during the COVID-19 pandemic compared with those before the pandemic, and region-wise subgroup analysis during the pandemic. (a) Survival to hospital discharge, (b) Return of spontaneous circulation, (c) Survival to hospitalization, (d) 30-day survival, and (e) Favorable neurological outcome.


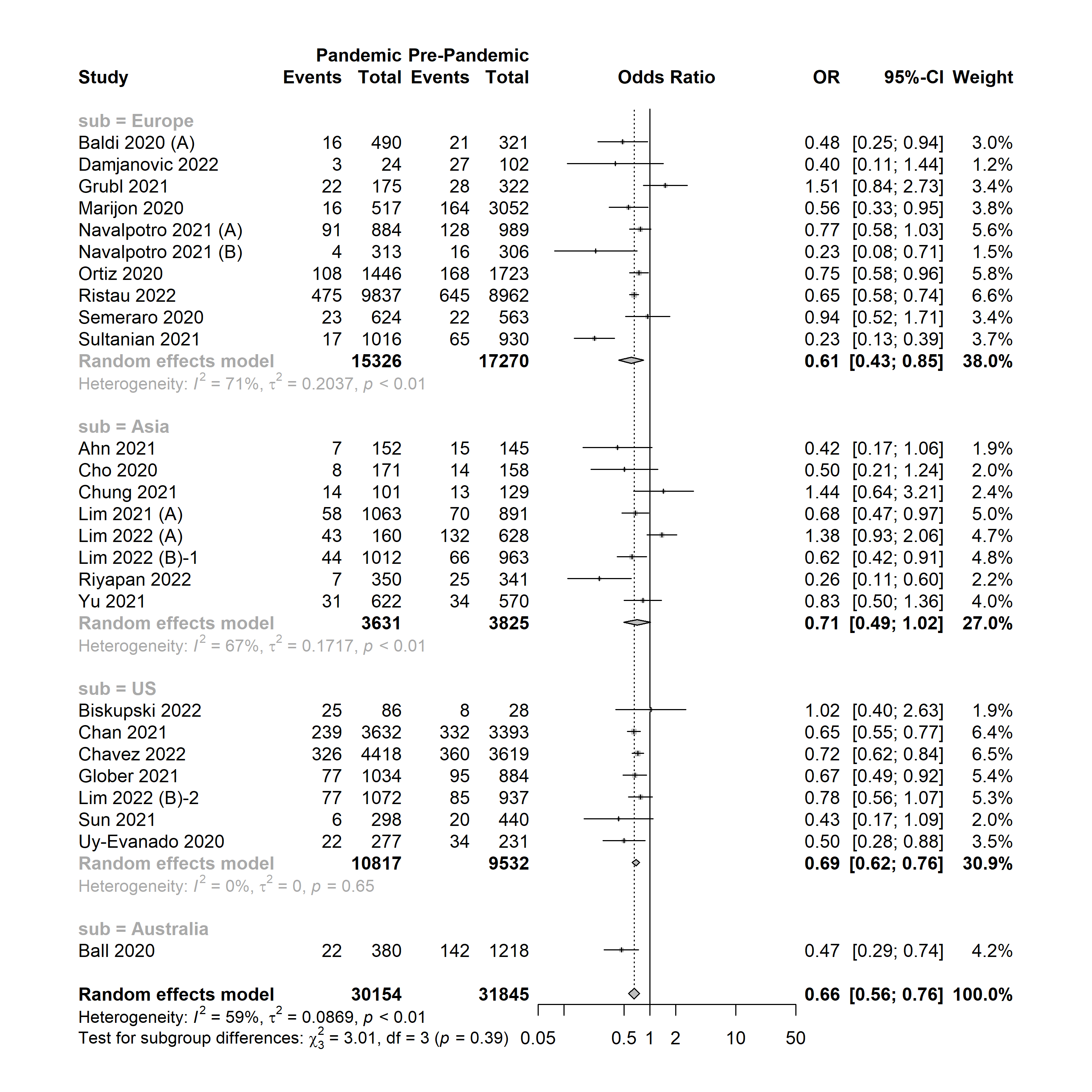


(a) Survival to hospital discharge


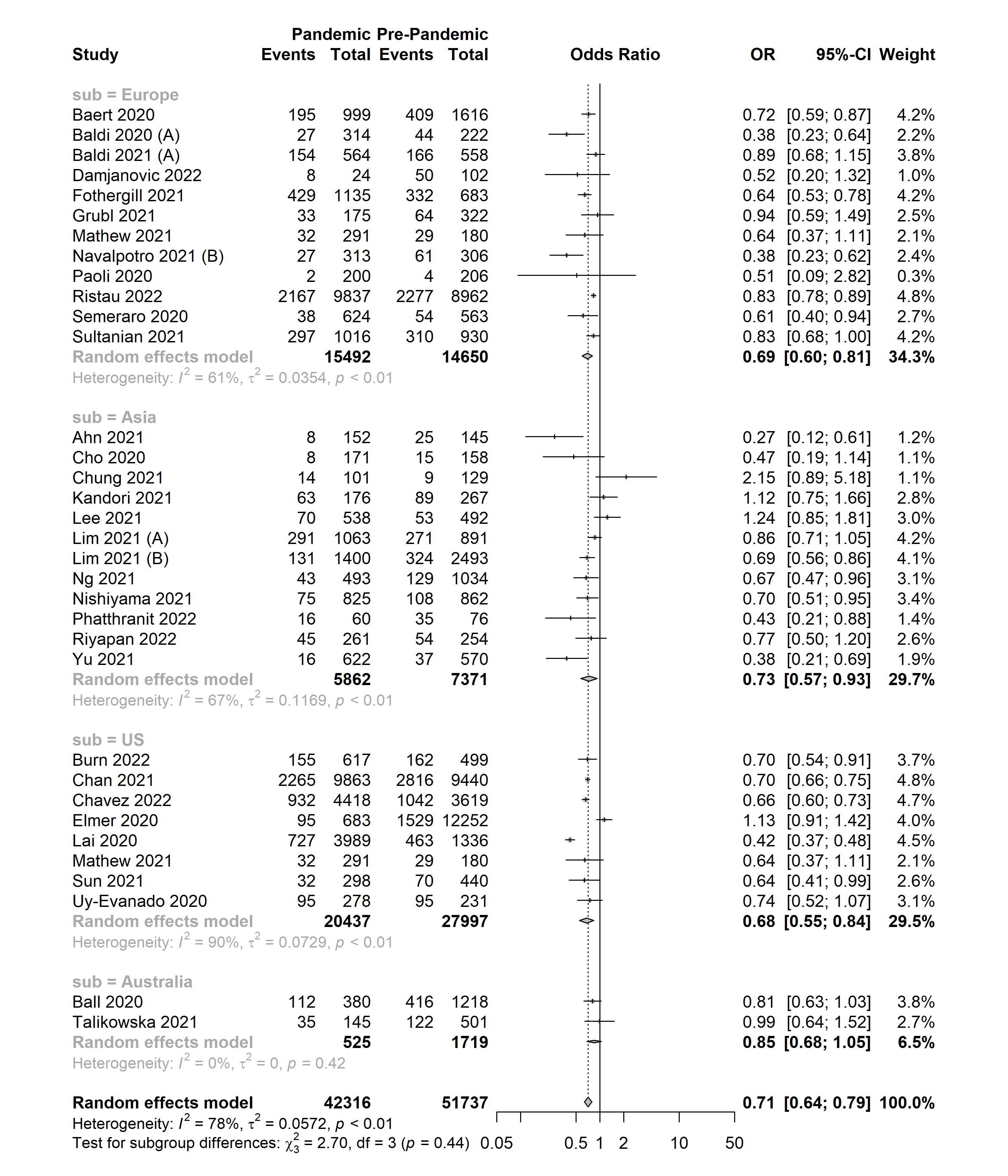


(b) Return of spontaneous circulation


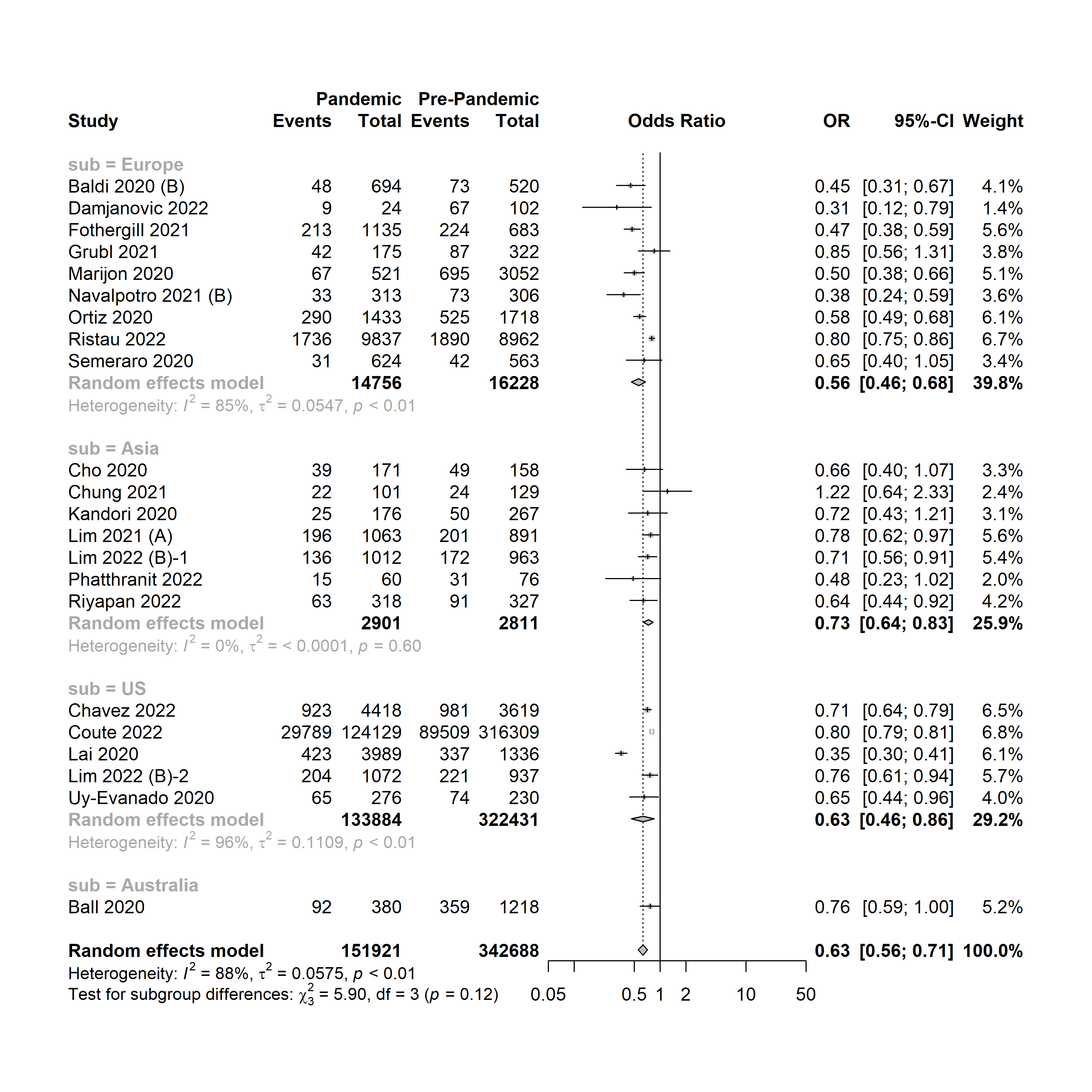


(c) Survival to hospital admission.


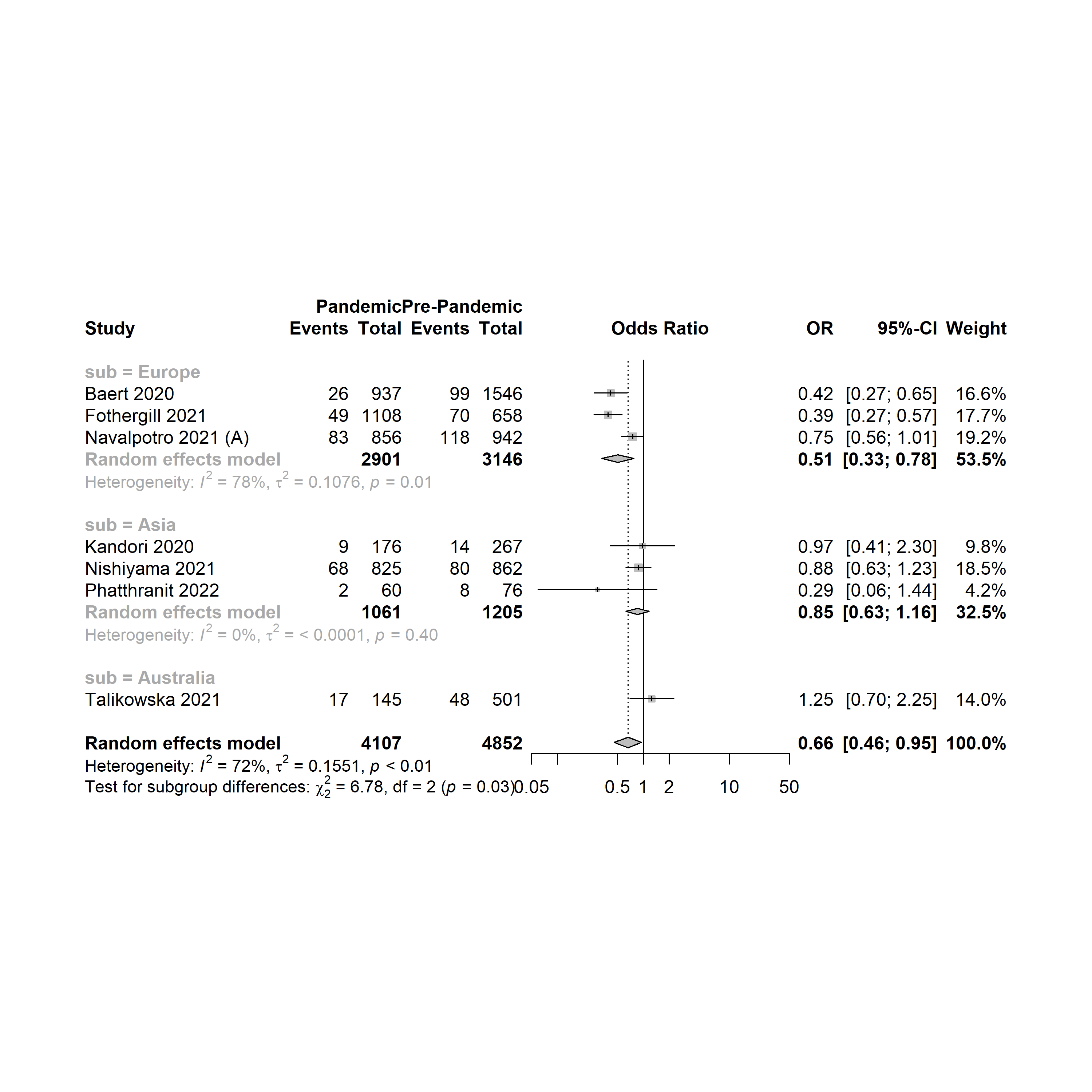


(d) 30-day survival


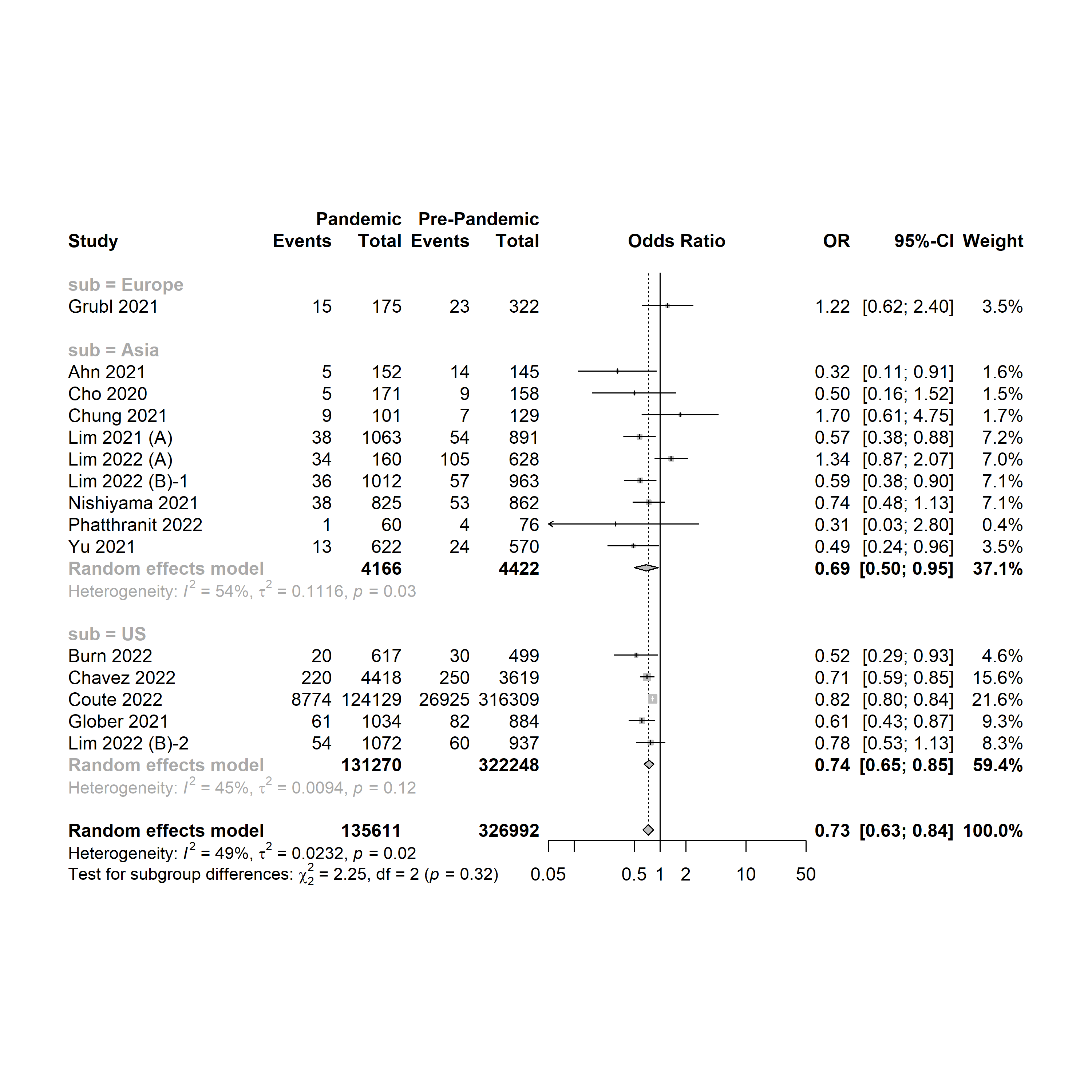


(e) Favorable neurological outcome

**Supplementary Figure 10.** Forest plot for epidemiologic factors during the COVID-19 pandemic compared with that before the pandemic, and subgroup analysis according to the study region during pandemic. (a) Cardiac arrest at home, (b) Use of automated external defibrillators, (c) Shockable rhythm, (d) Unwitnessed cardiac arrest, and (e) Bystander CPR.


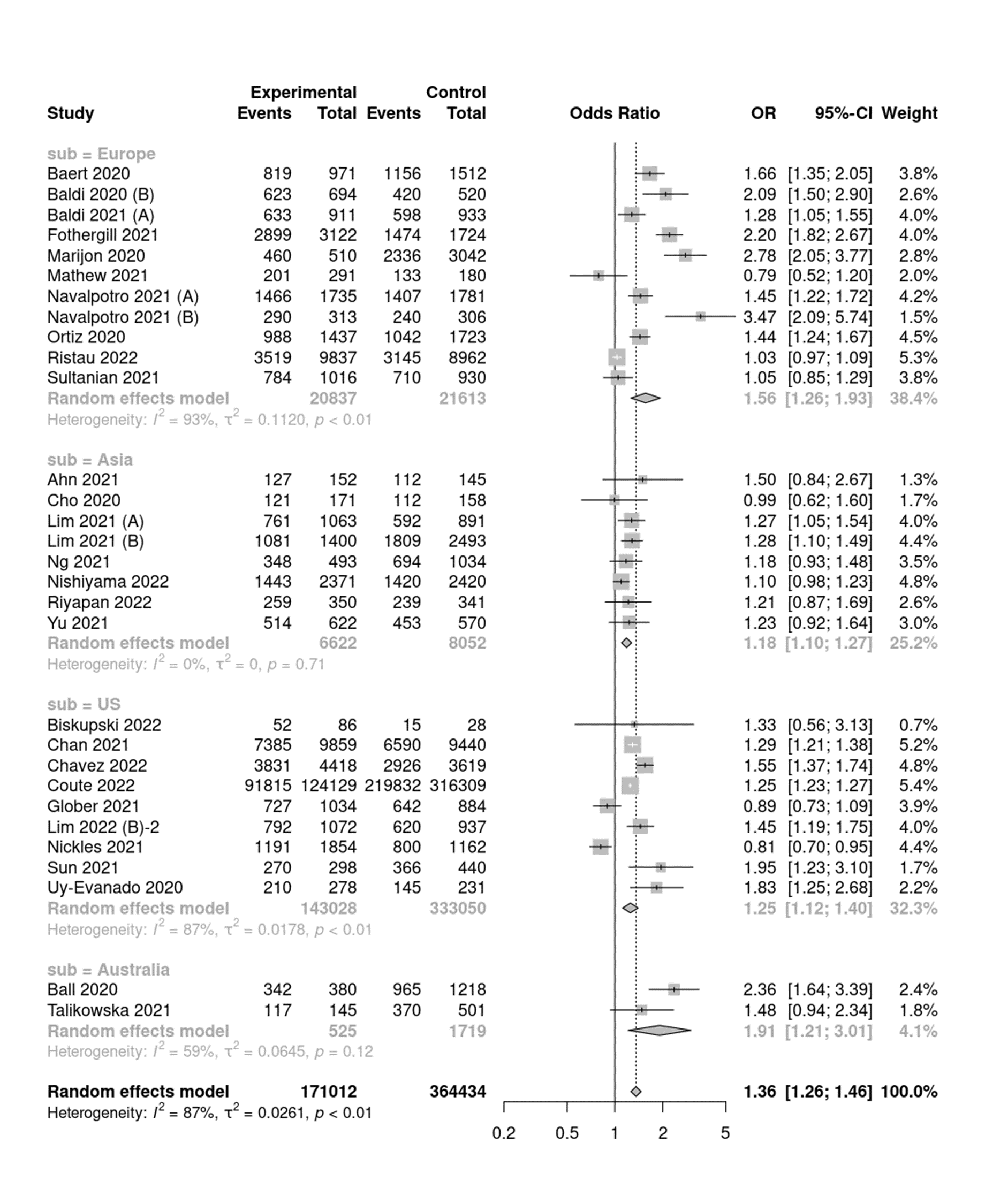


(a) Arrest at home


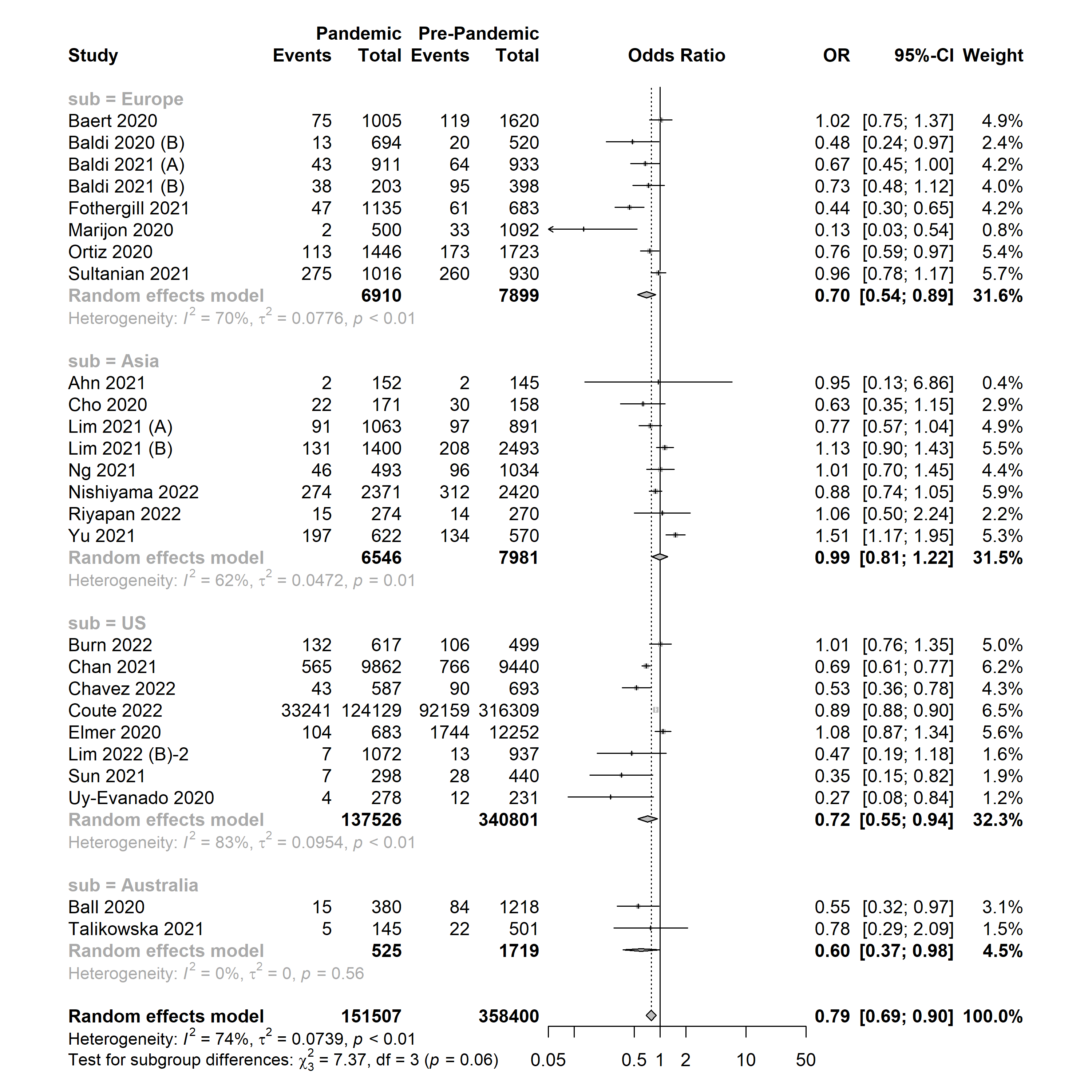


(b) Use of automated external defibrillators


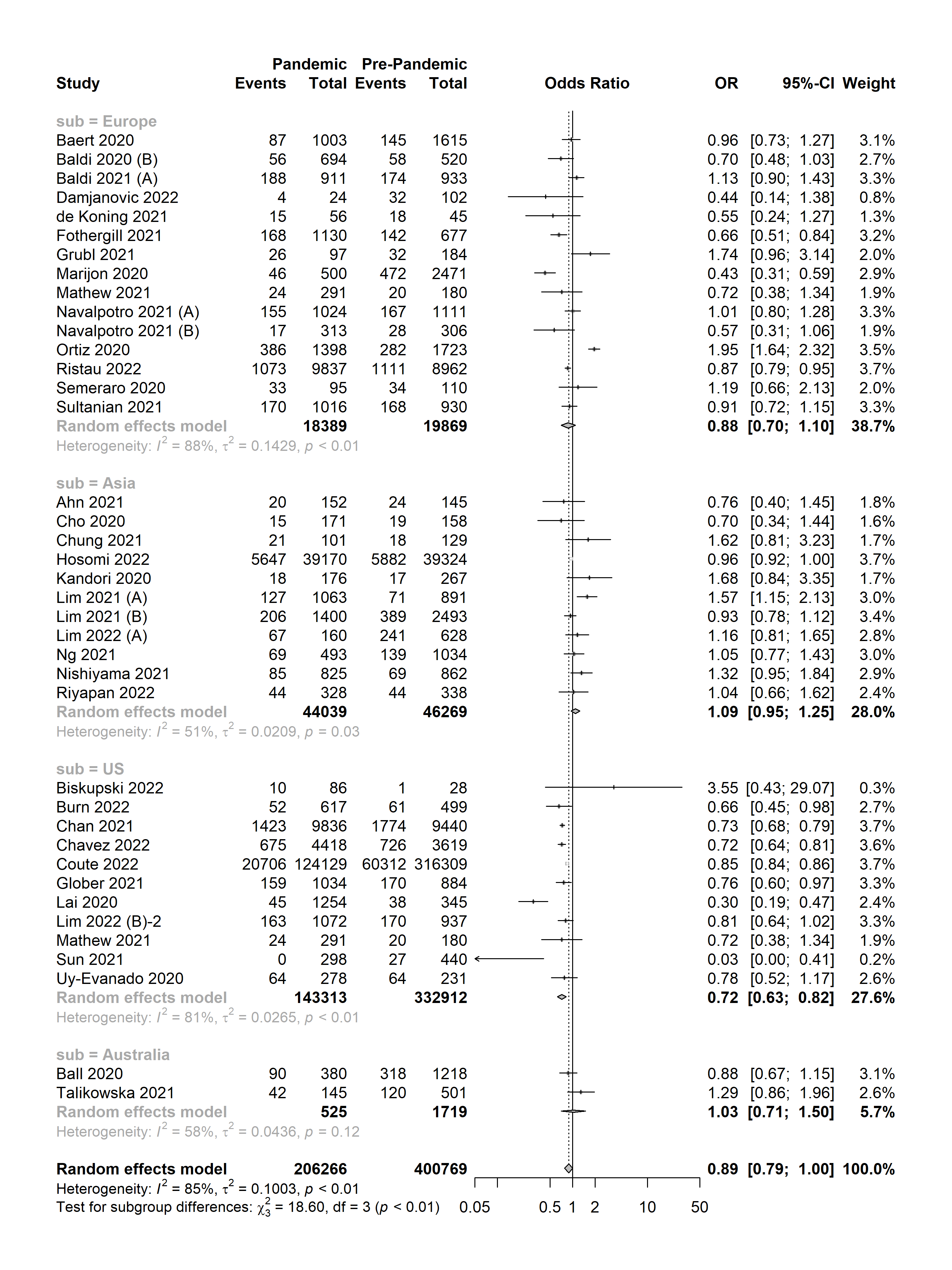


(c) Shockable rhythm


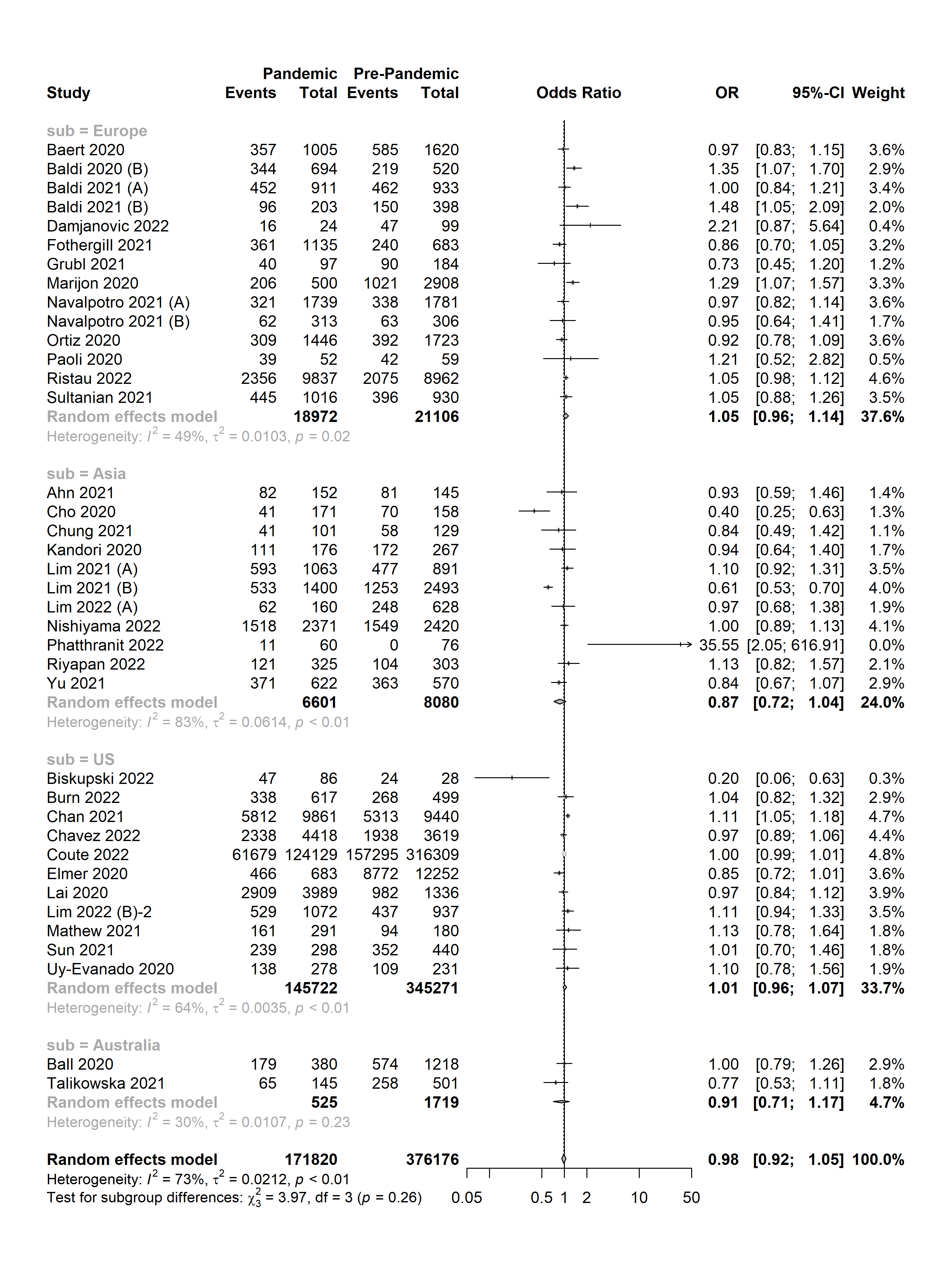


(d) Unwitnessed cardiac arrest


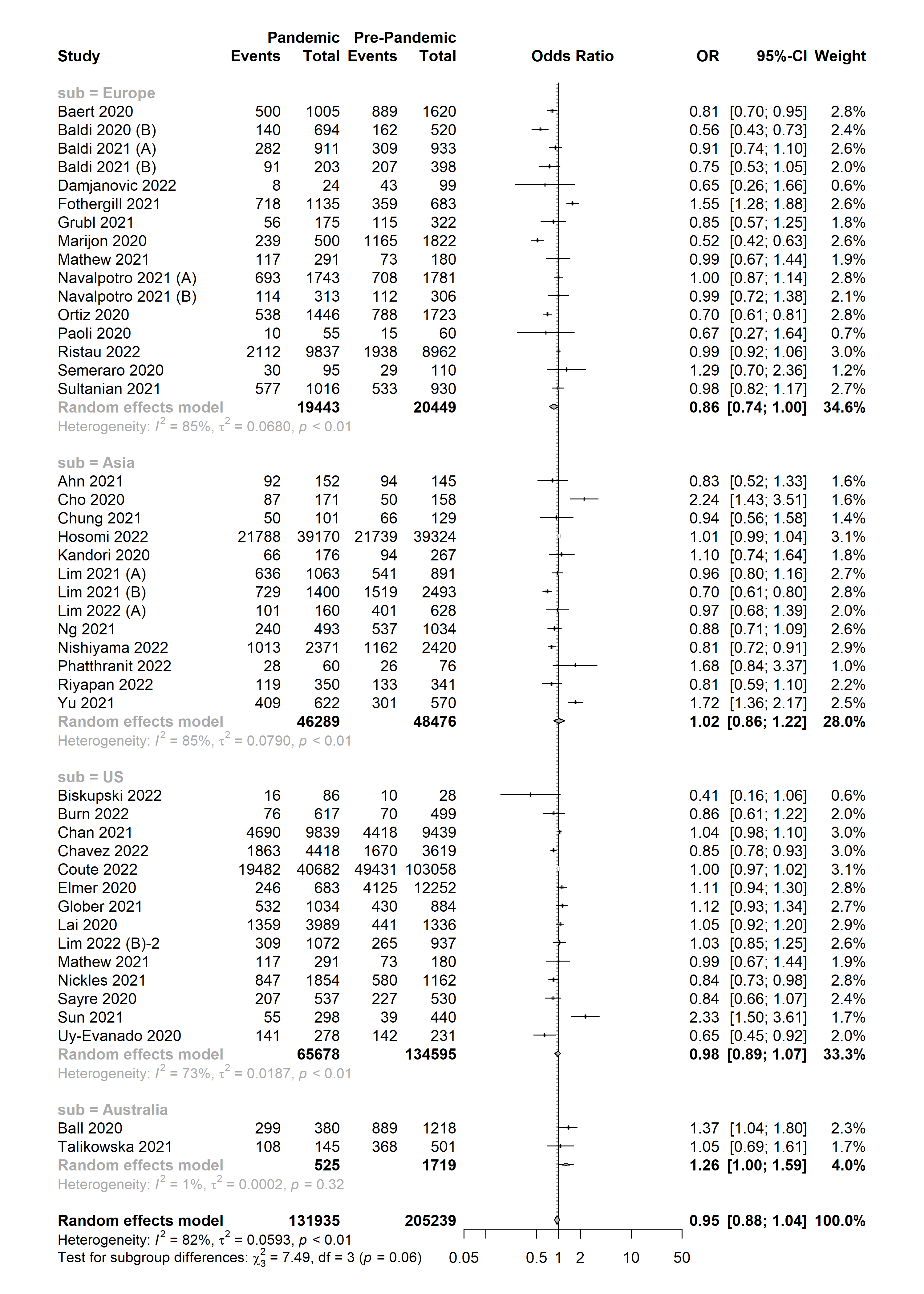


(e) Bystander CPR

**Supplementary Figure 11.** Forest plot depicting prehospital factors during the COVID-19 pandemic compared with that before the pandemic, and region-wise subgroup analysis of the pandemic period. (a) EMS response time, (b) EMS transportation time, (c) Supraglottic airway device, (d) Endotracheal intubation, (e) Mechanical CPR, and (f) Prehospital return of spontaneous circulation.


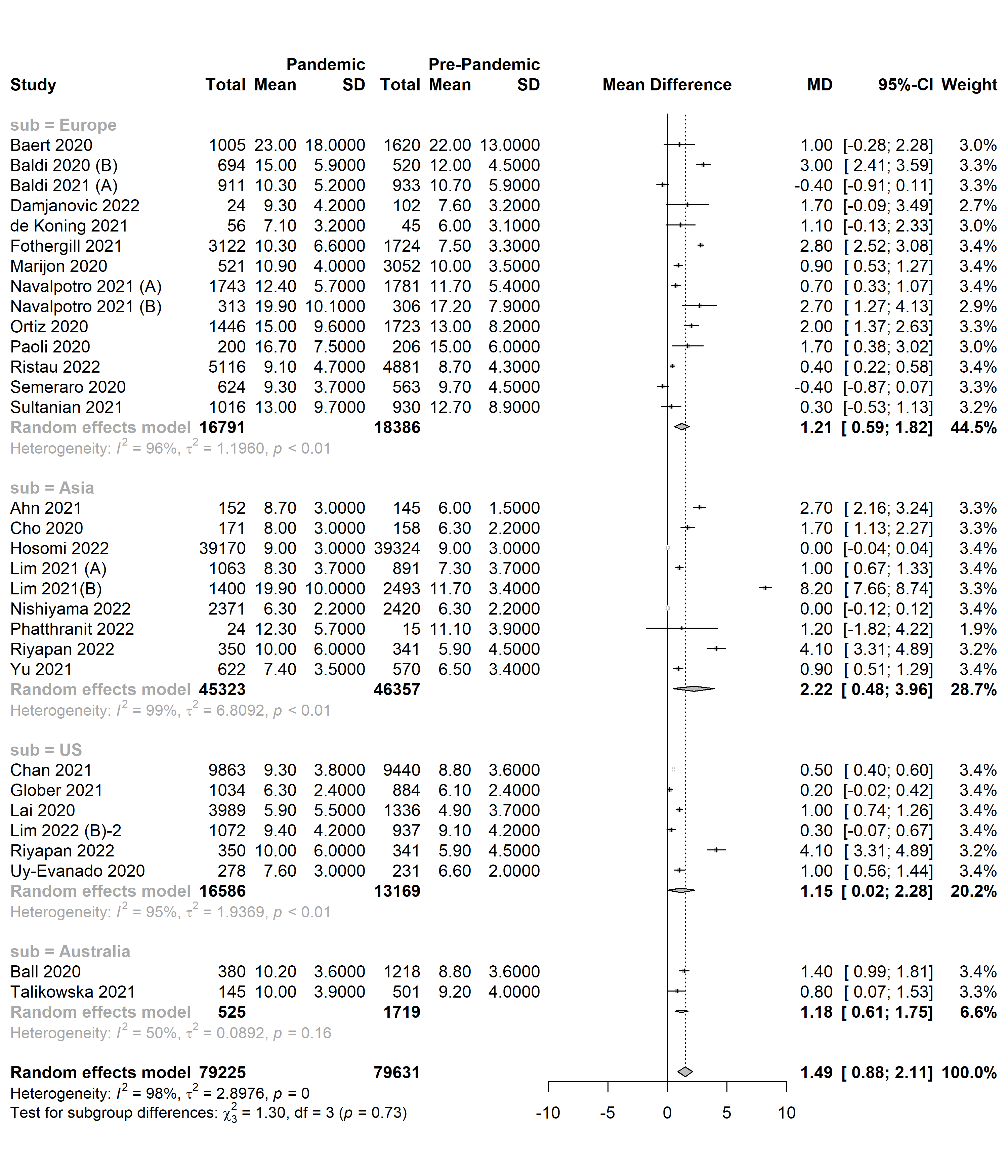


(a) EMS response time


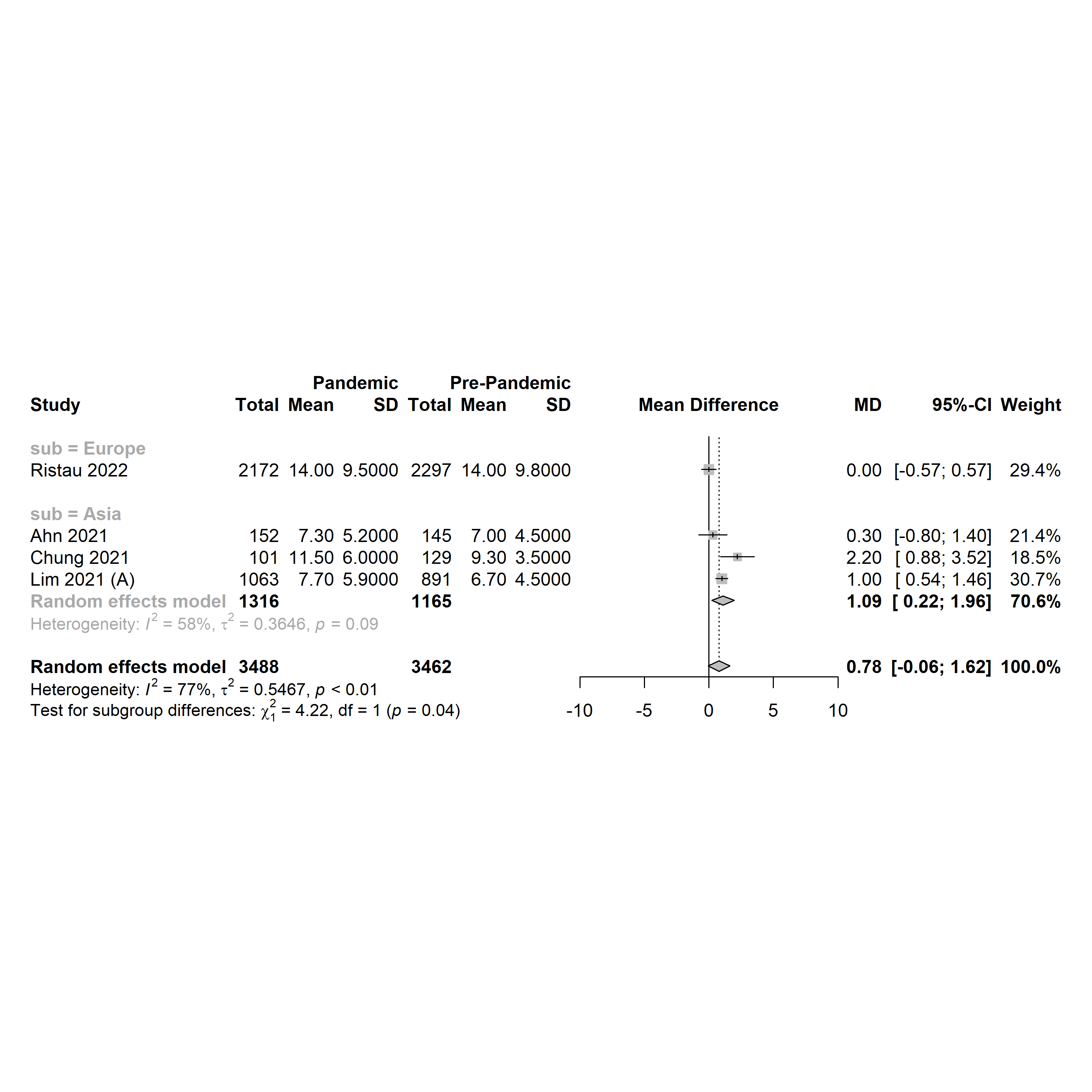


(b) EMS transport time


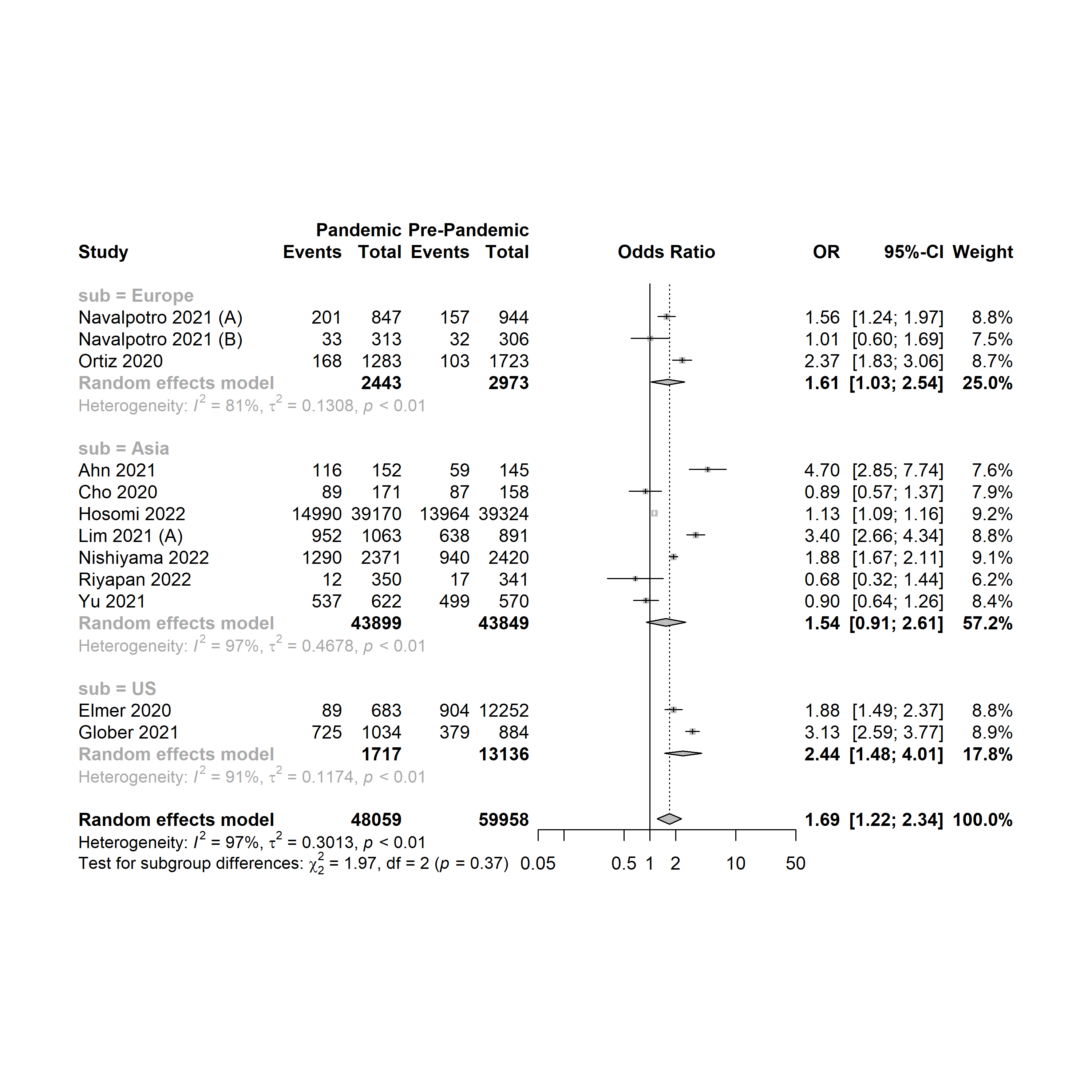


(c) Supraglottic airway device


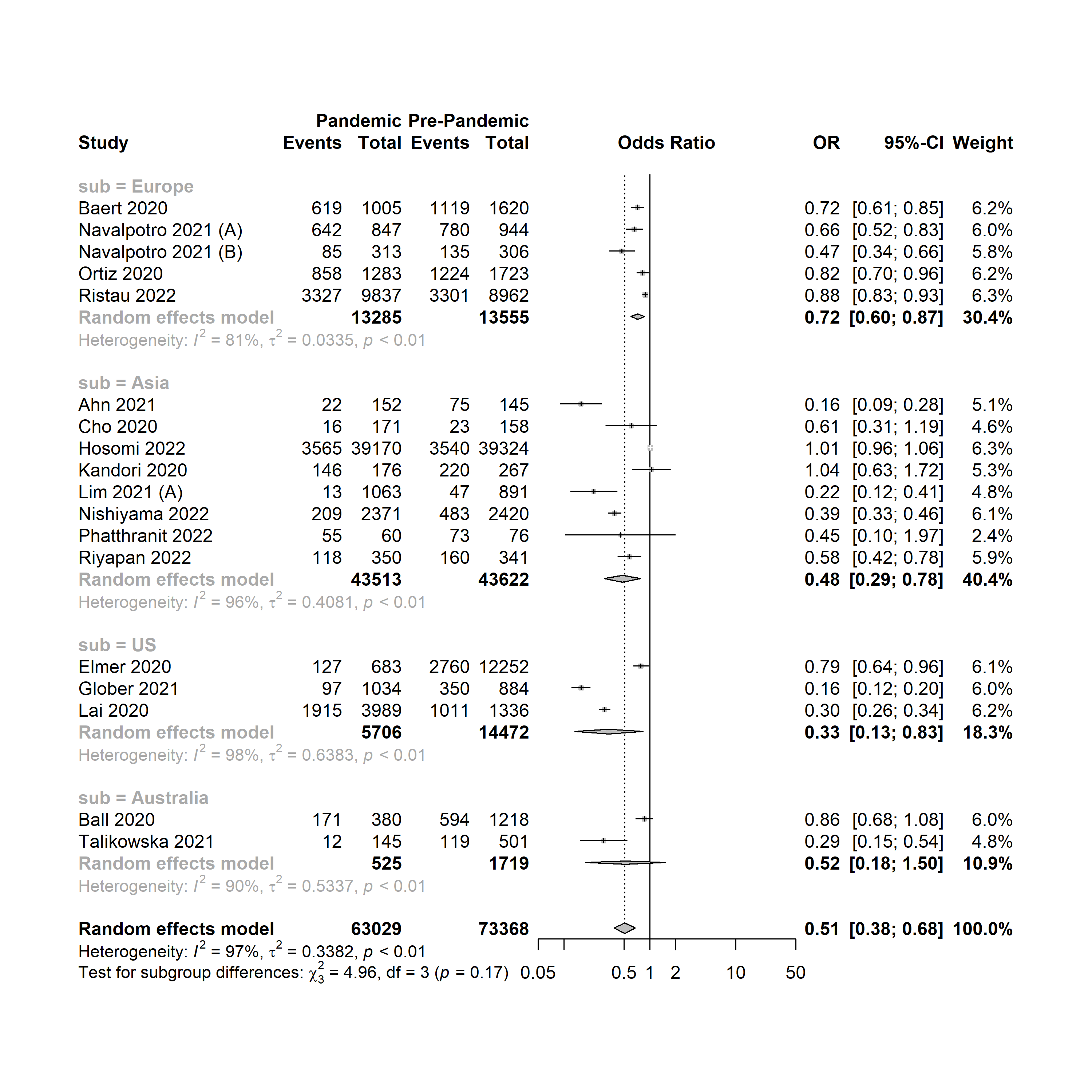


(d) Endotracheal intubation


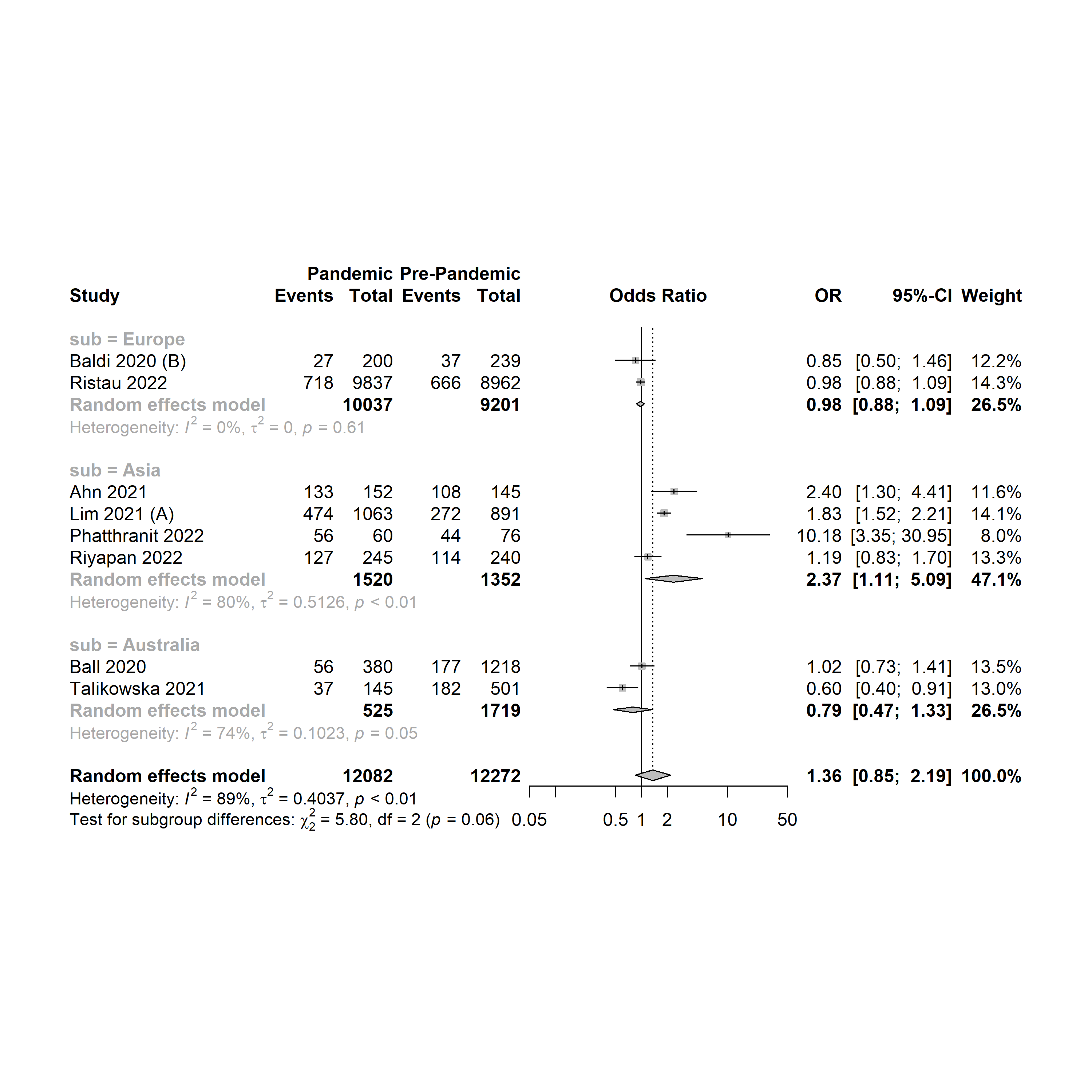


(e) Mechanical CPR


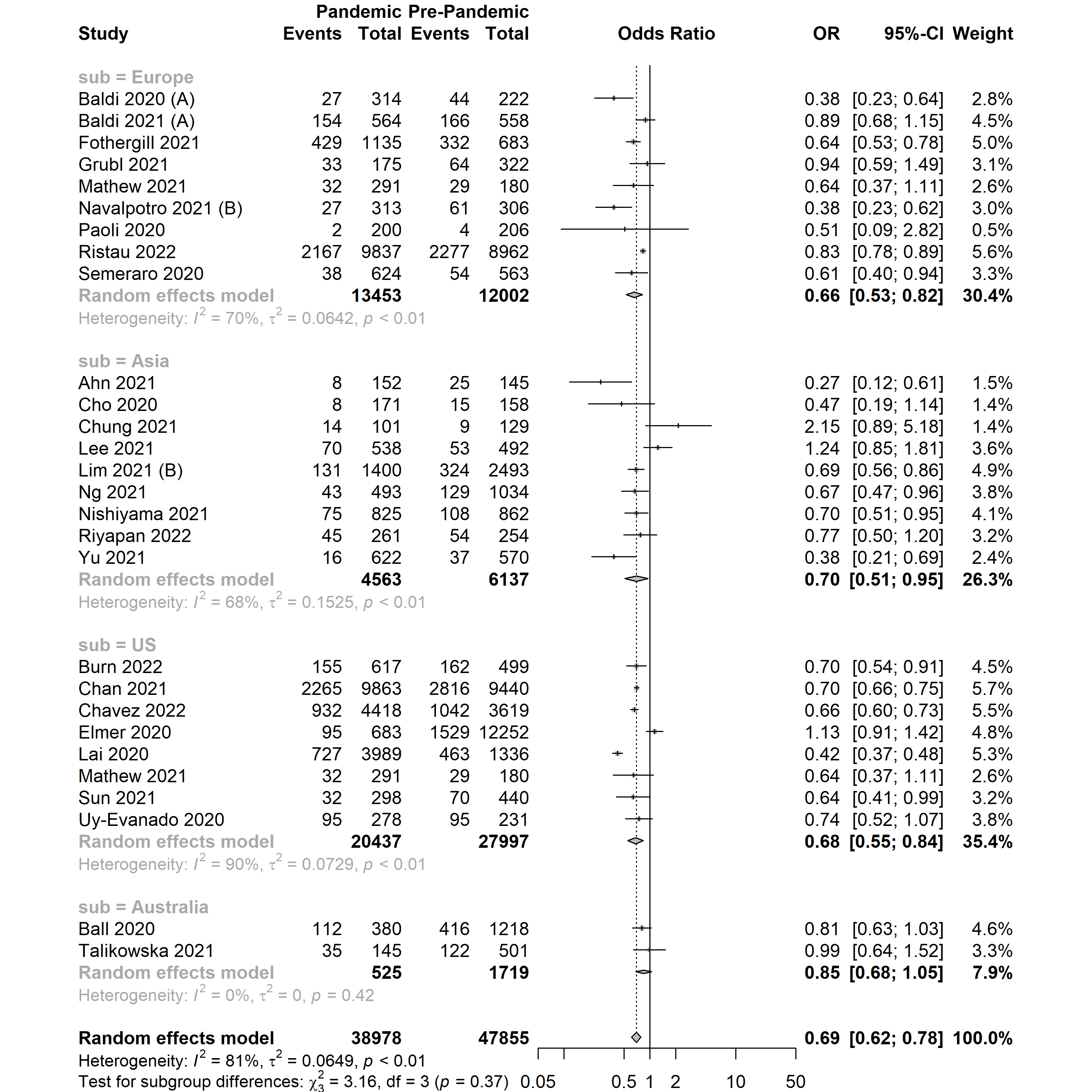


(f) Prehospital return of spontaneous circulation

**Supplementary Figure 12.** Forest plot for target temperature management during the COVID-19 pandemic compared with that before the pandemic, and region-wise subgroup analysis of the pandemic period.


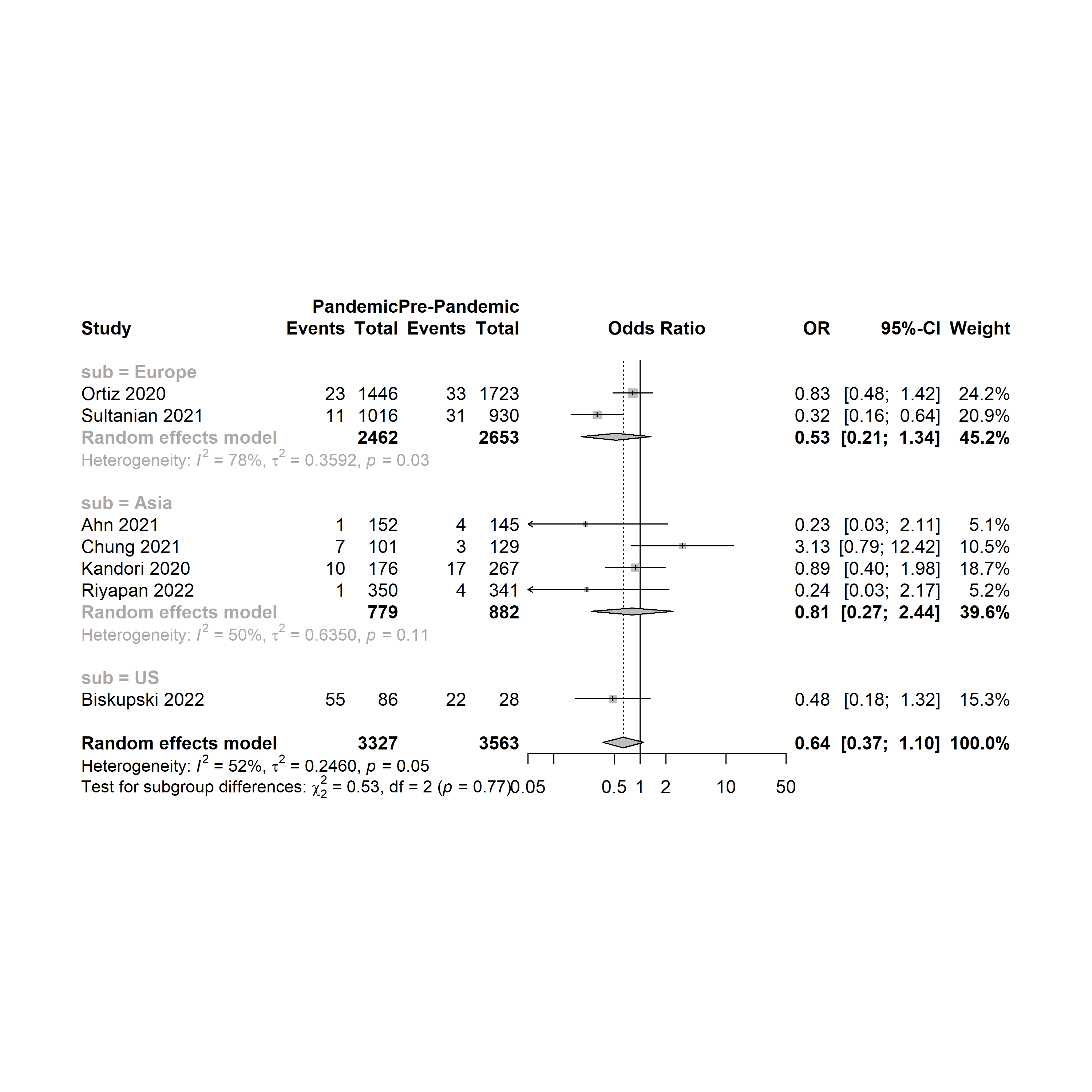


**Supplementary Figure 13.** Publication bias was calculated according to each outcome and factor. (a) Survival to hospital discharge, (b) Return of spontaneous circulation, (c) Survival to hospital admission, (d) 30-day survival, (e) Favorable neurological outcome, (f) Arrest at home, (g) Use of automated external defibrillators, (h)Shockable rhythm, (i) Unwitnessed cardiac arrest, (j) Bystander CPR, (k) EMS response time, (l) EMS transport time, (m) Supraglottic airway device, (n) Endotracheal intubation, (o) Mechanical CPR, (p) Prehospital return of spontaneous circulation, and (q) Target temperature management.


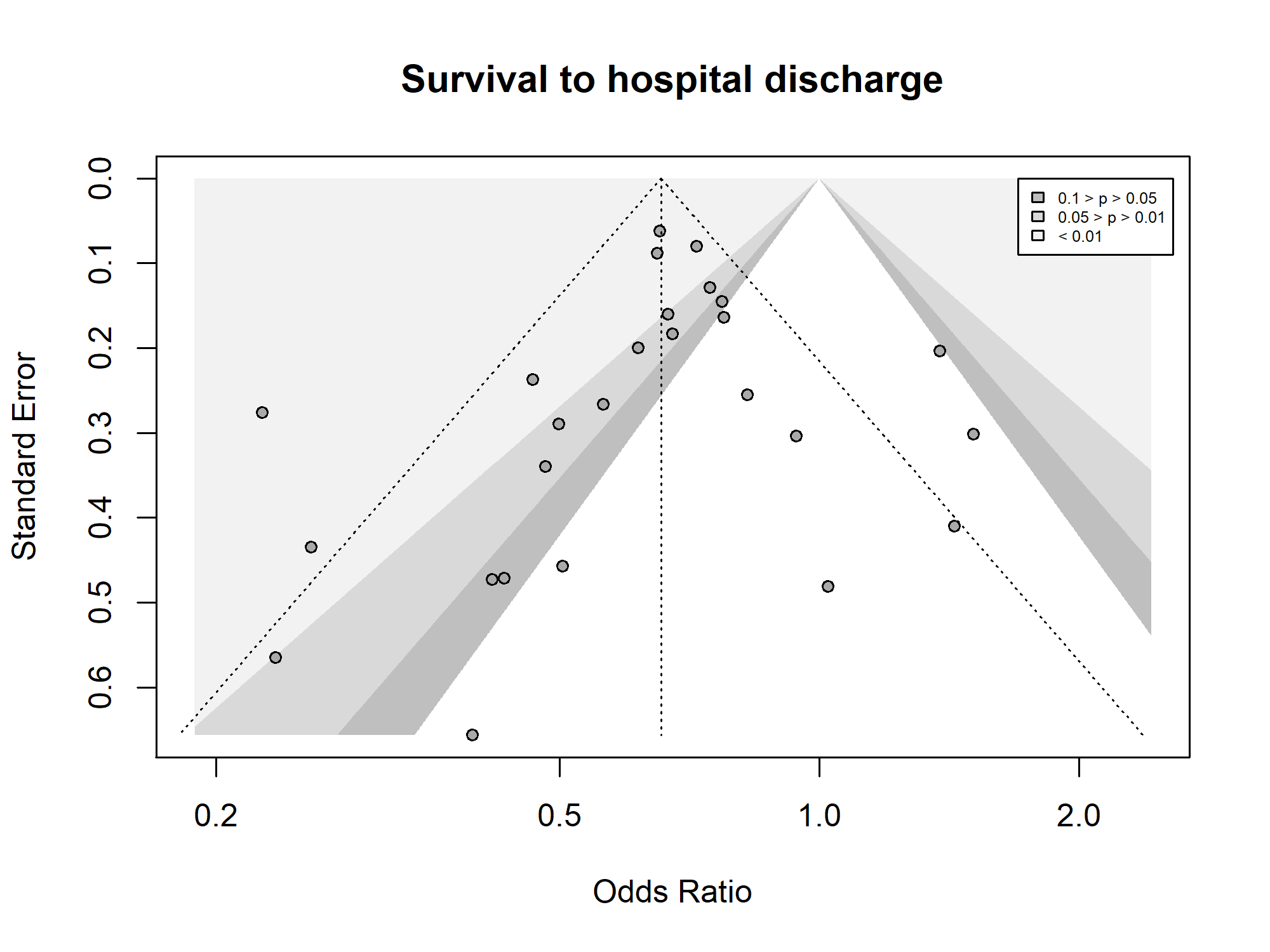


(a) Survival to hospital discharge


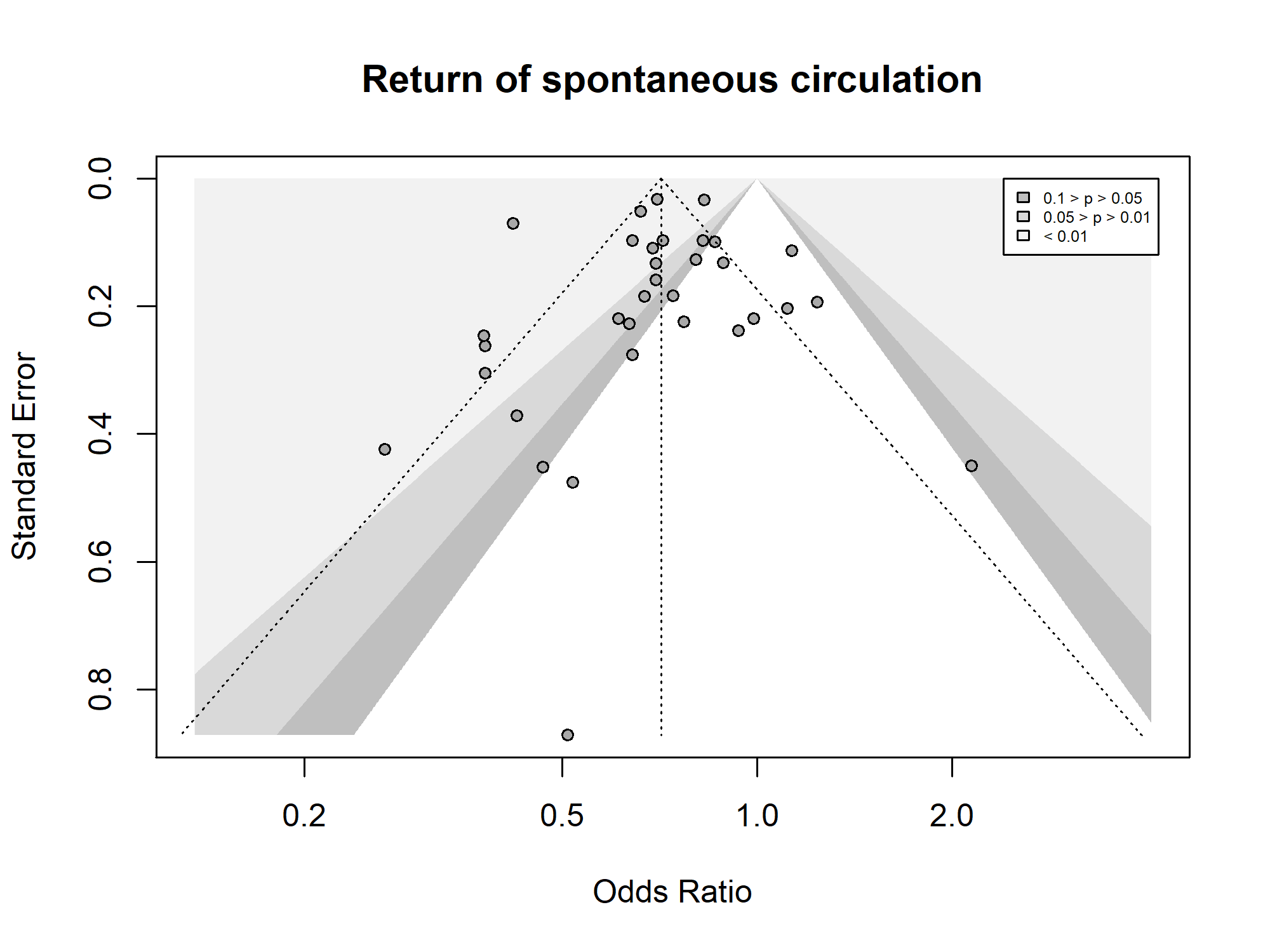


(b) Return of spontaneous circulation


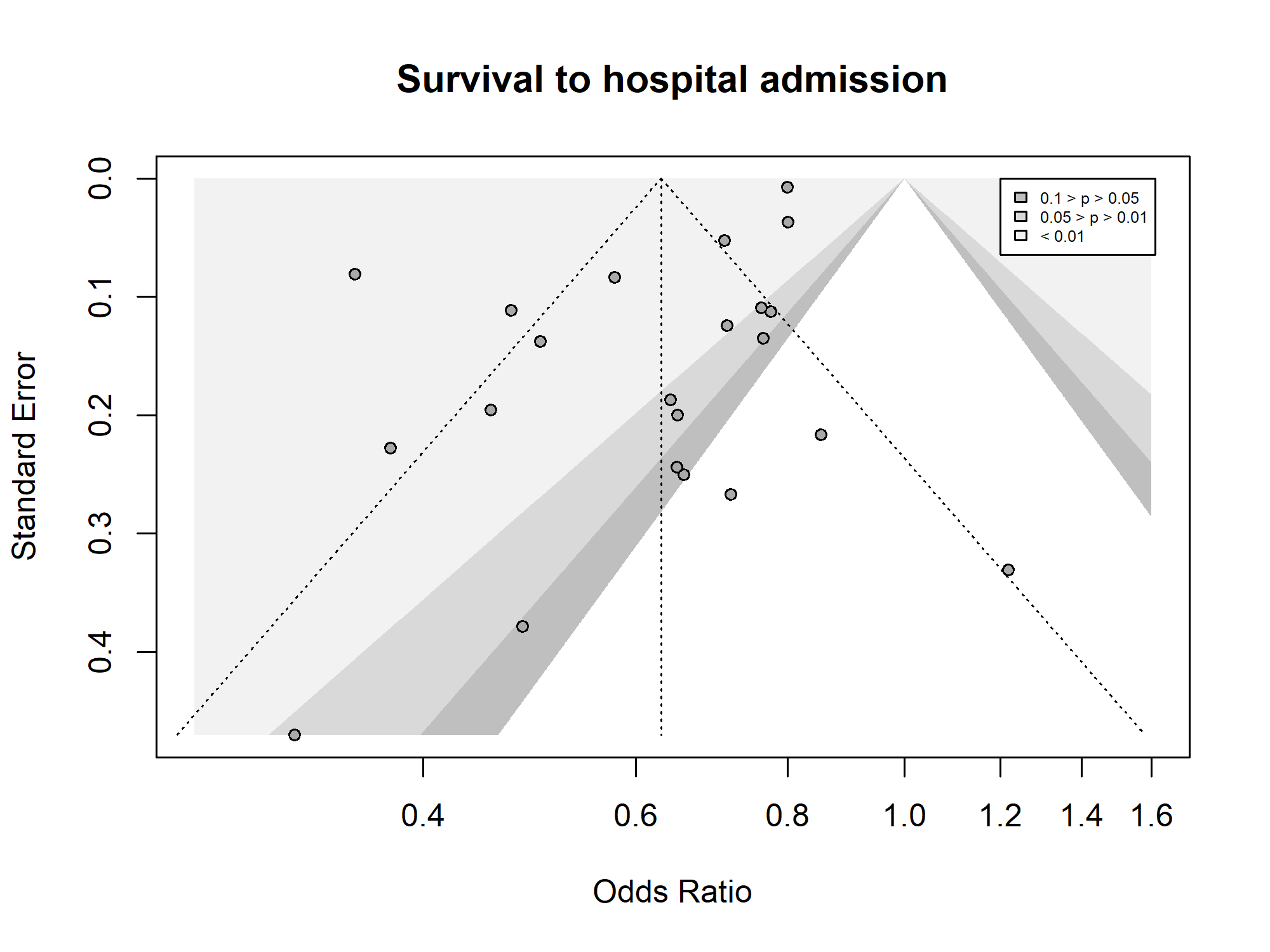


(c) Survival to hospital admission


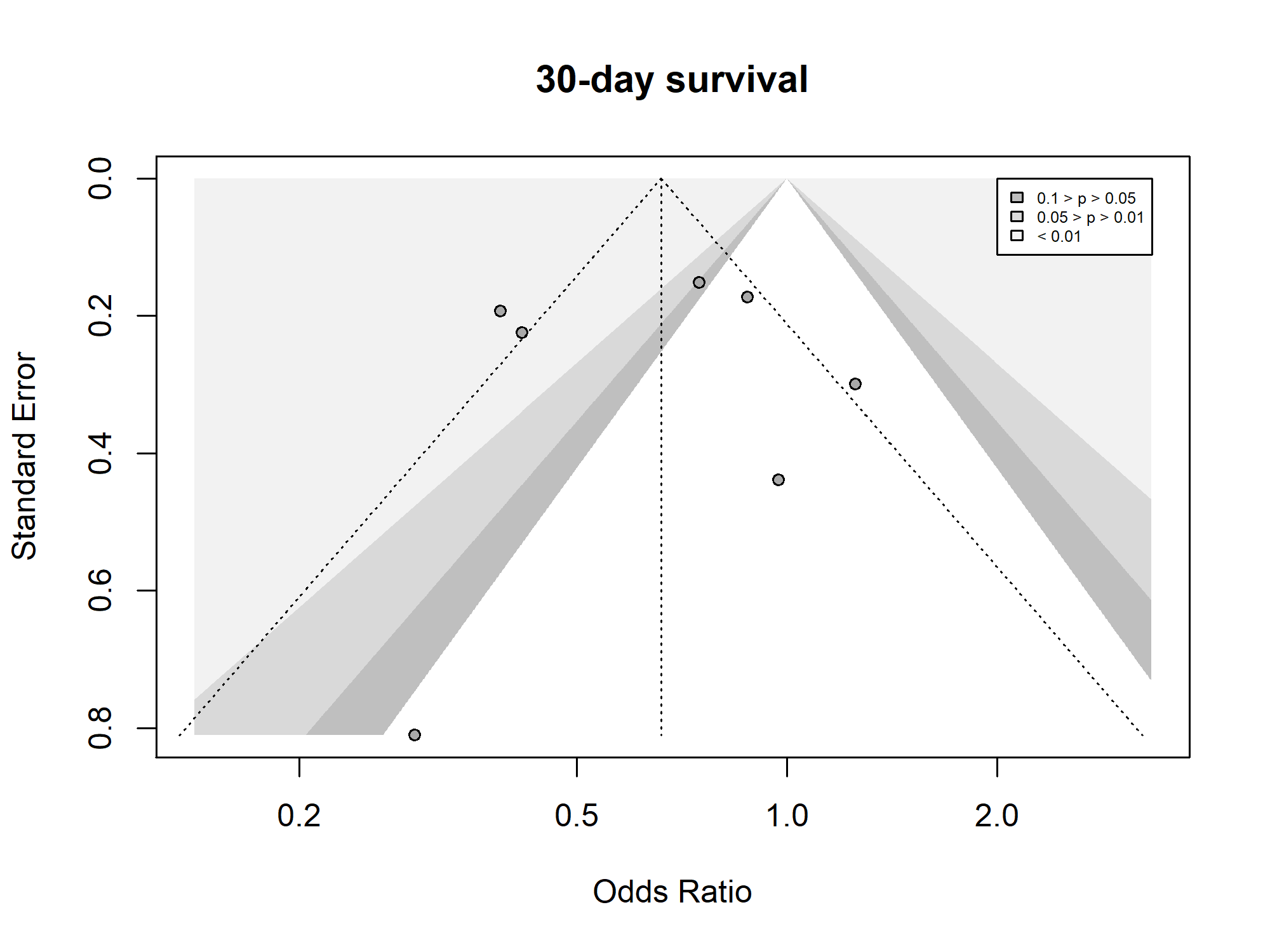


(d) 30-day survival


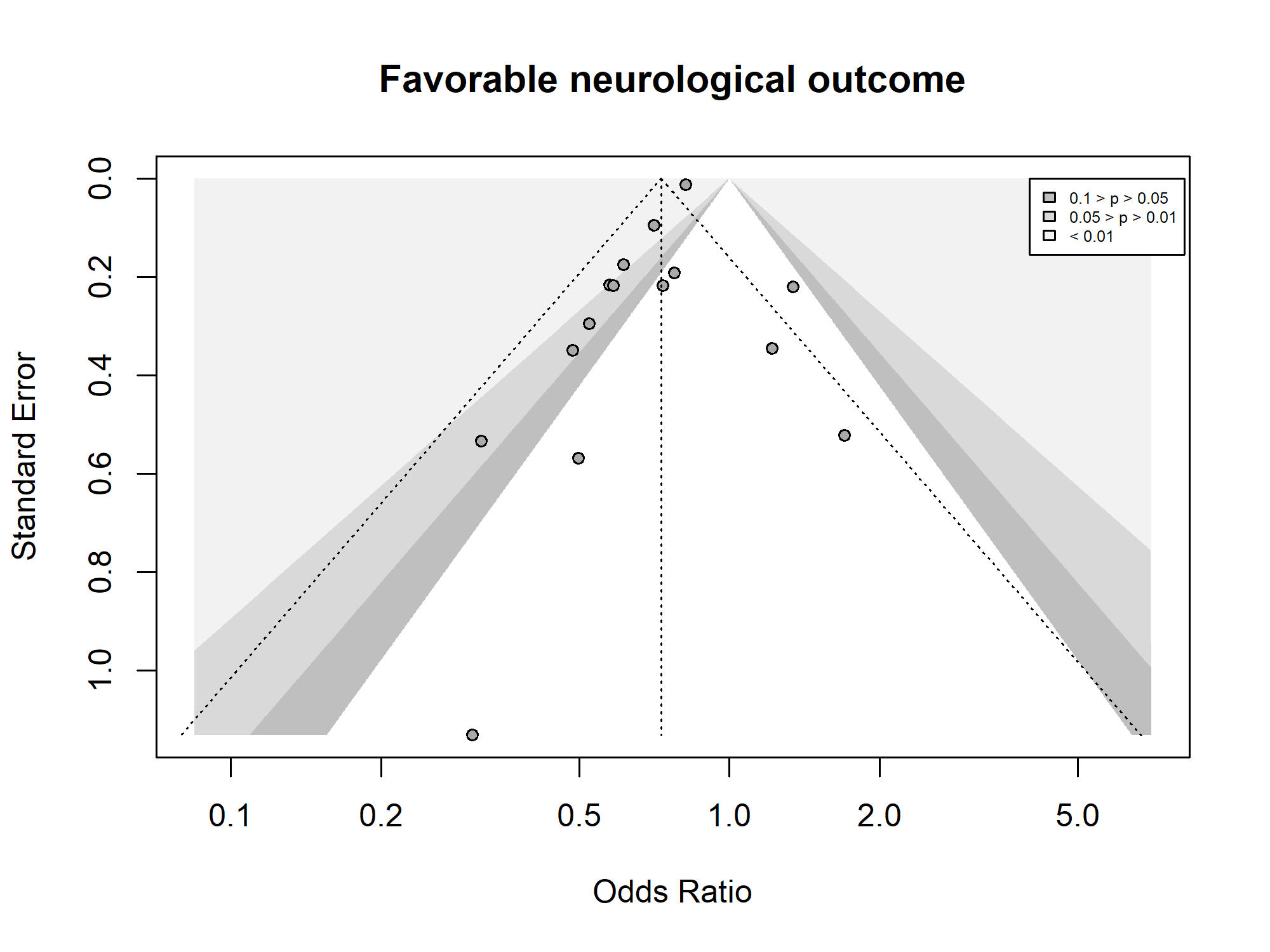


(e) Favorable neurological outcome


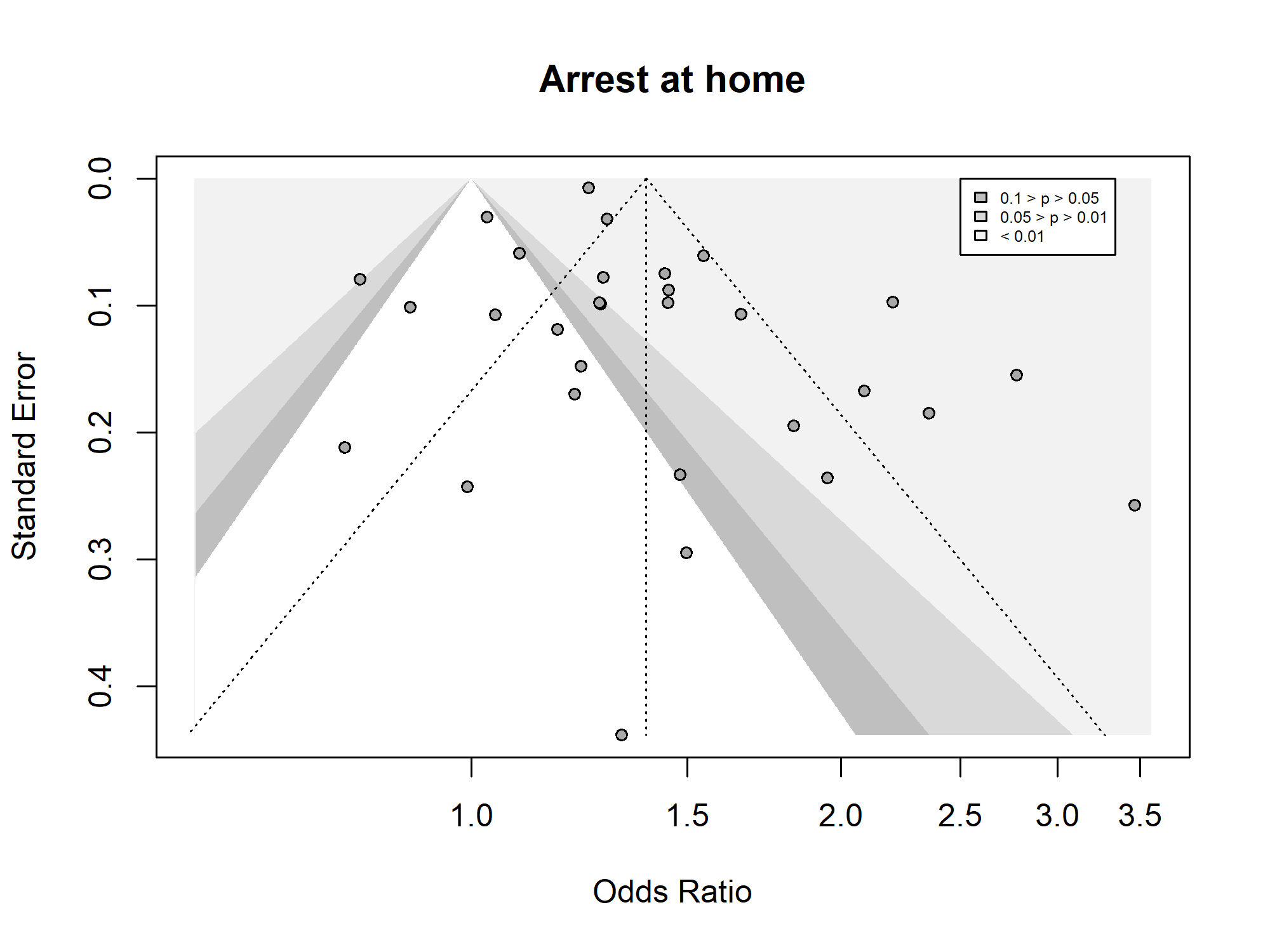


(f) Arrest at home


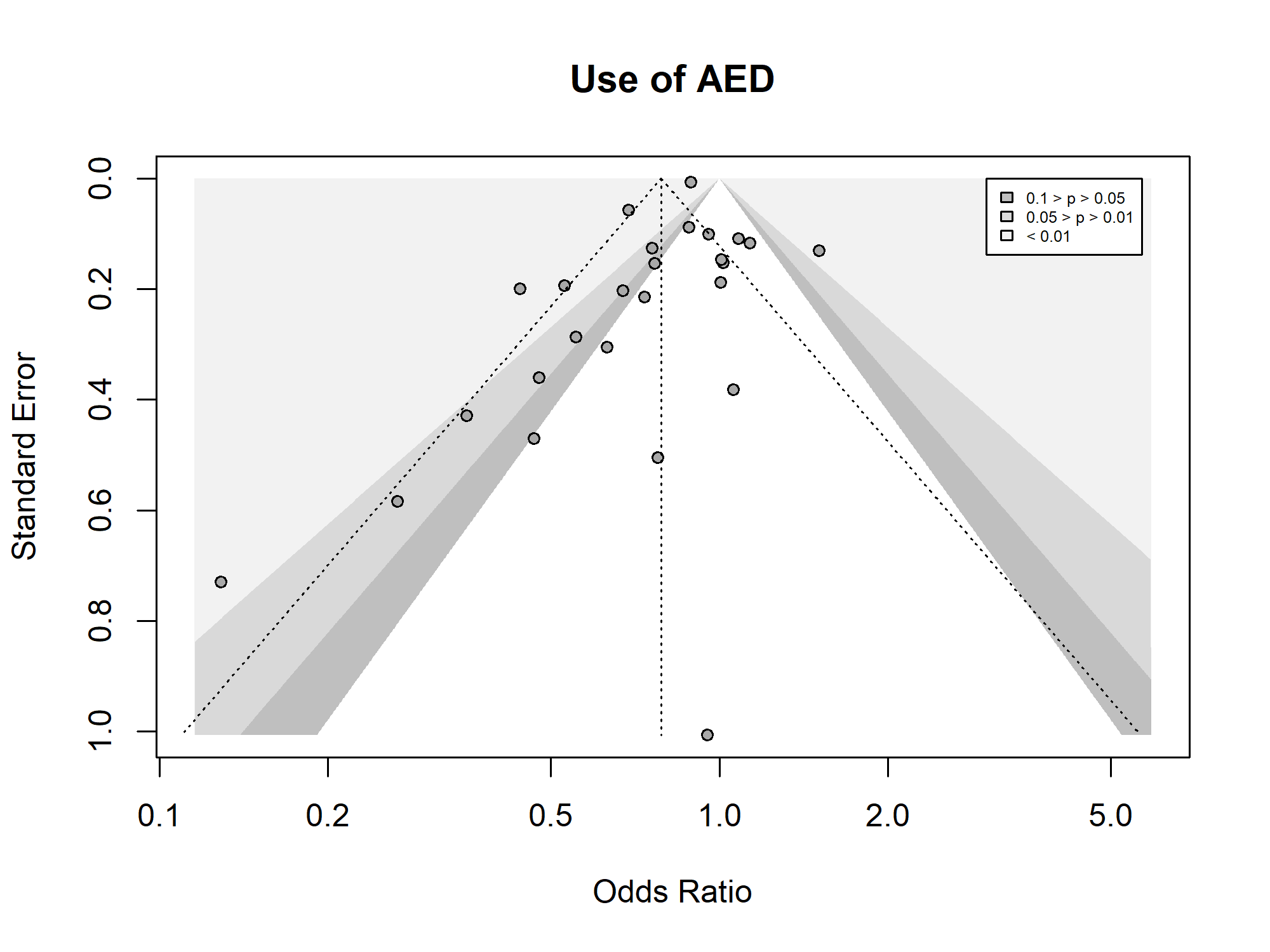


(g) Use of automated external defibrillators.


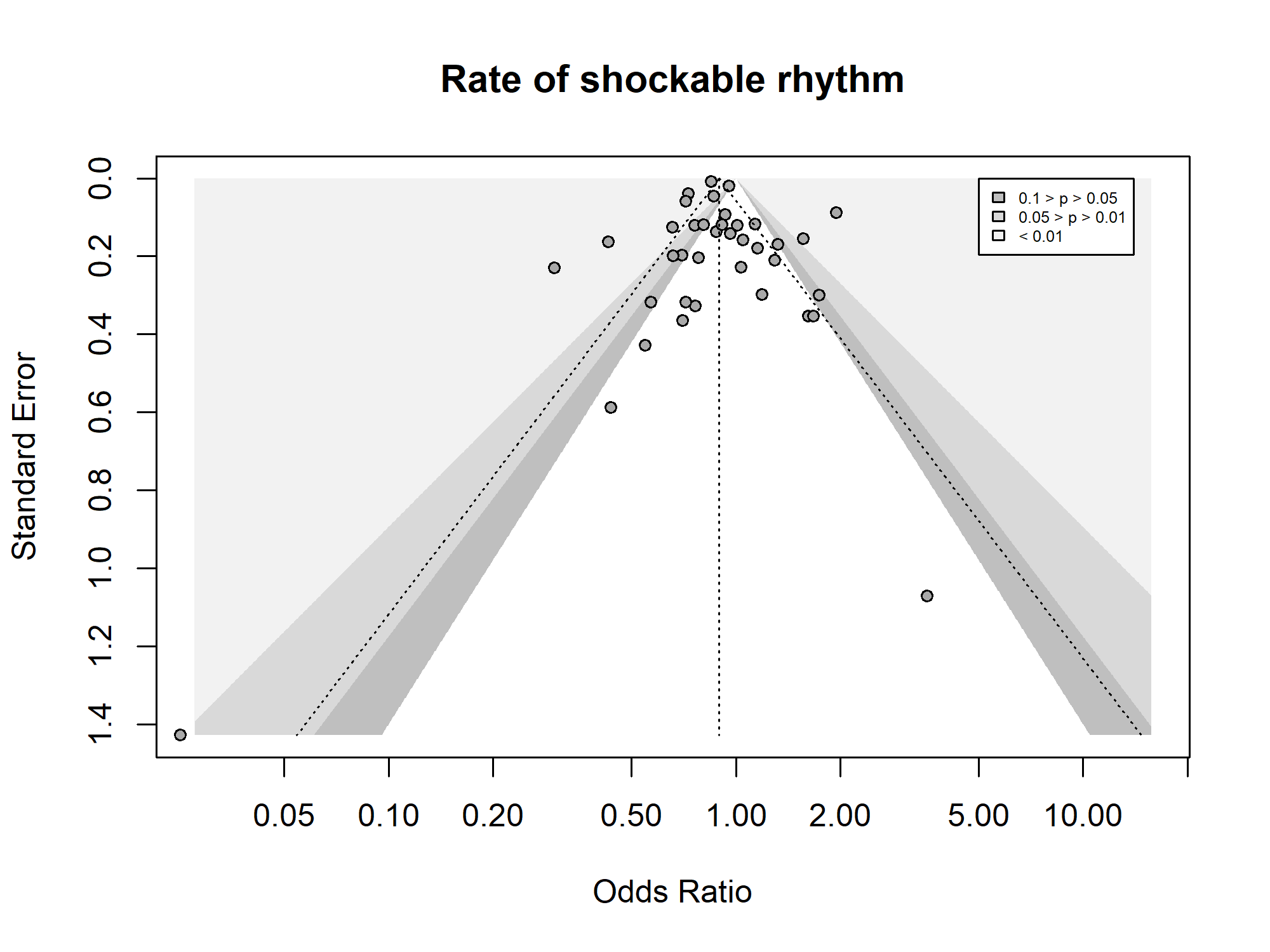


(h) Shockable rhythm.


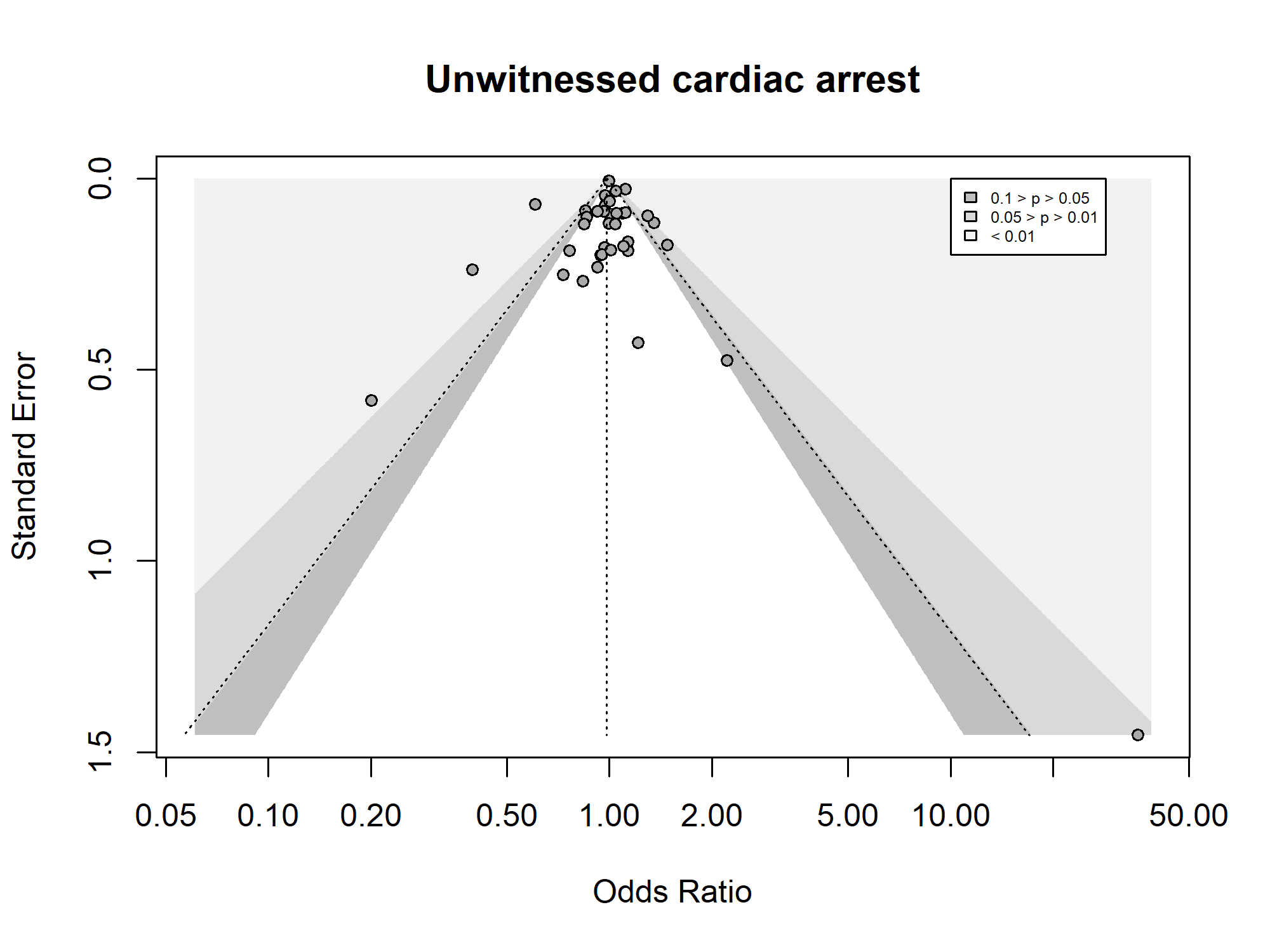


(i) Unwitnessed cardiac arrest.


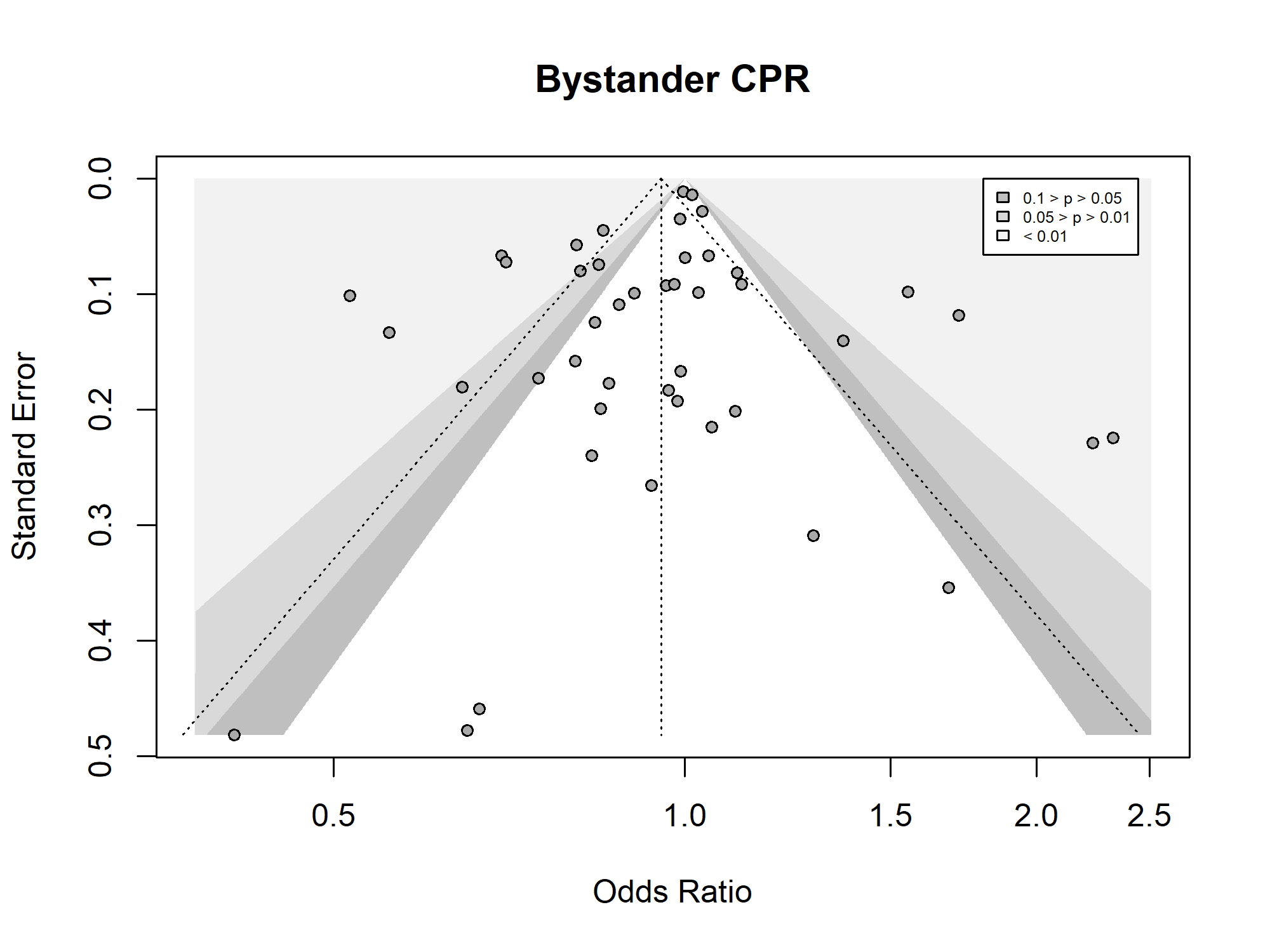


(j) Bystander CPR


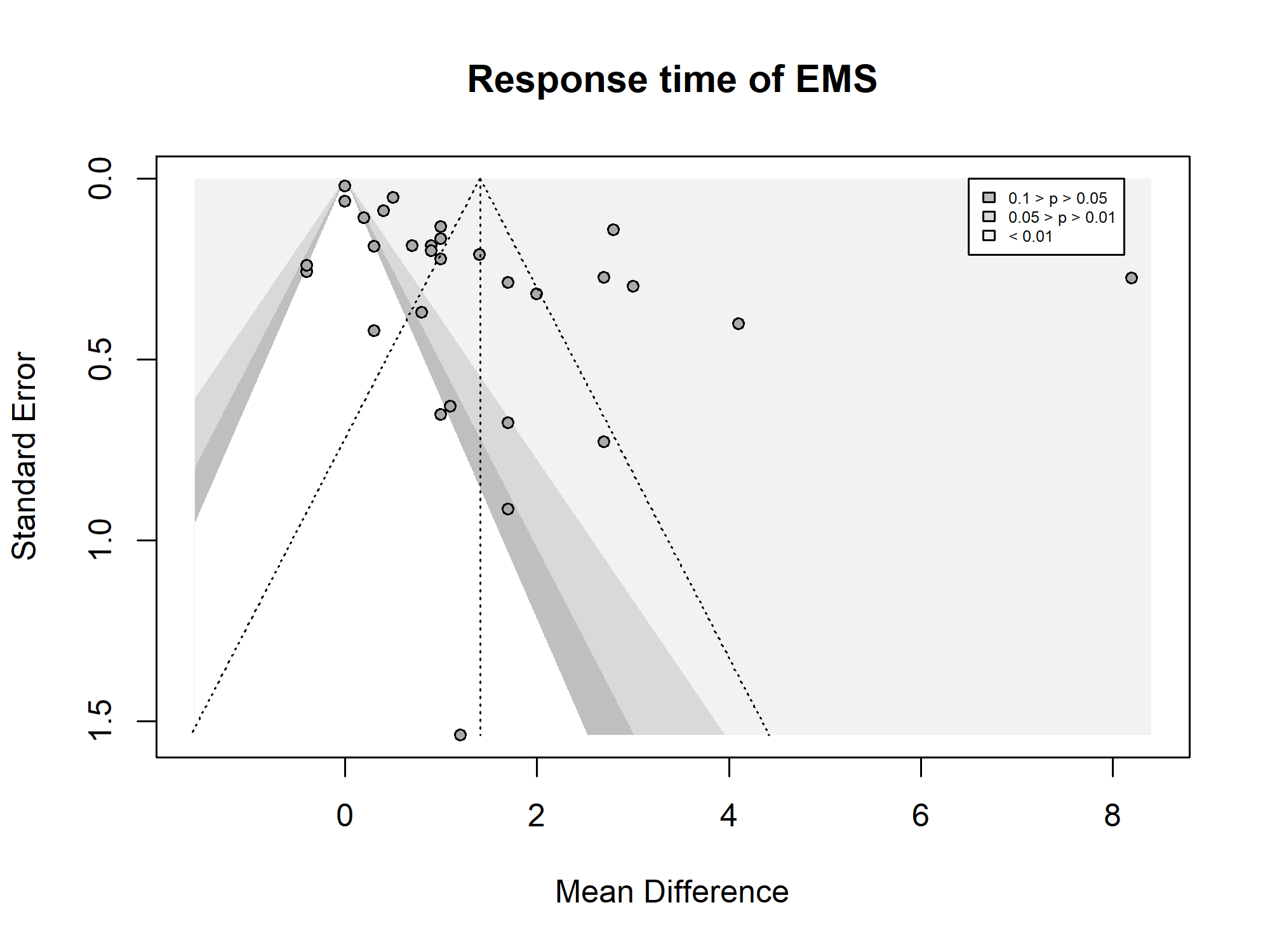


(k) EMS response time


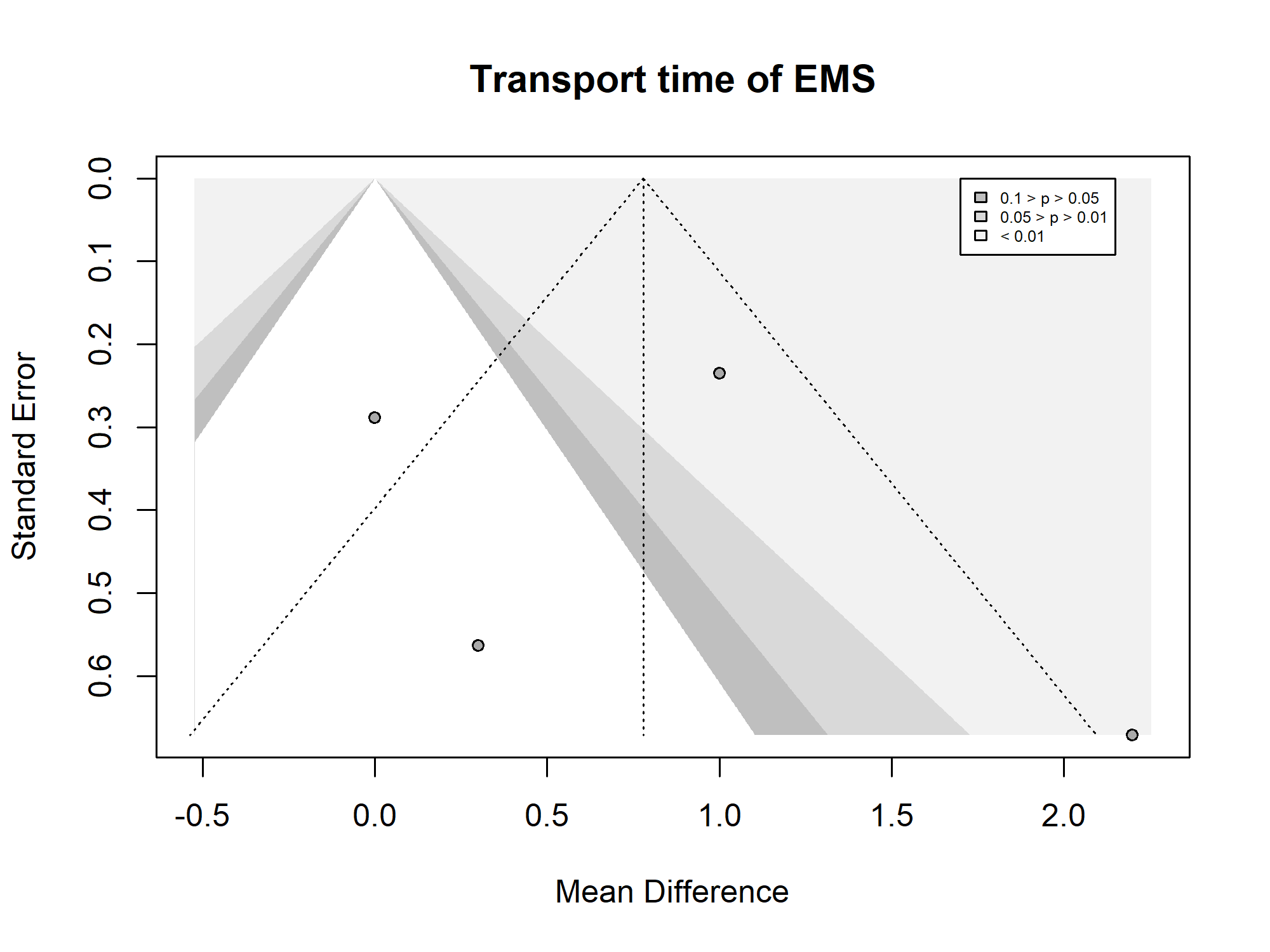


(l) EMS transport time


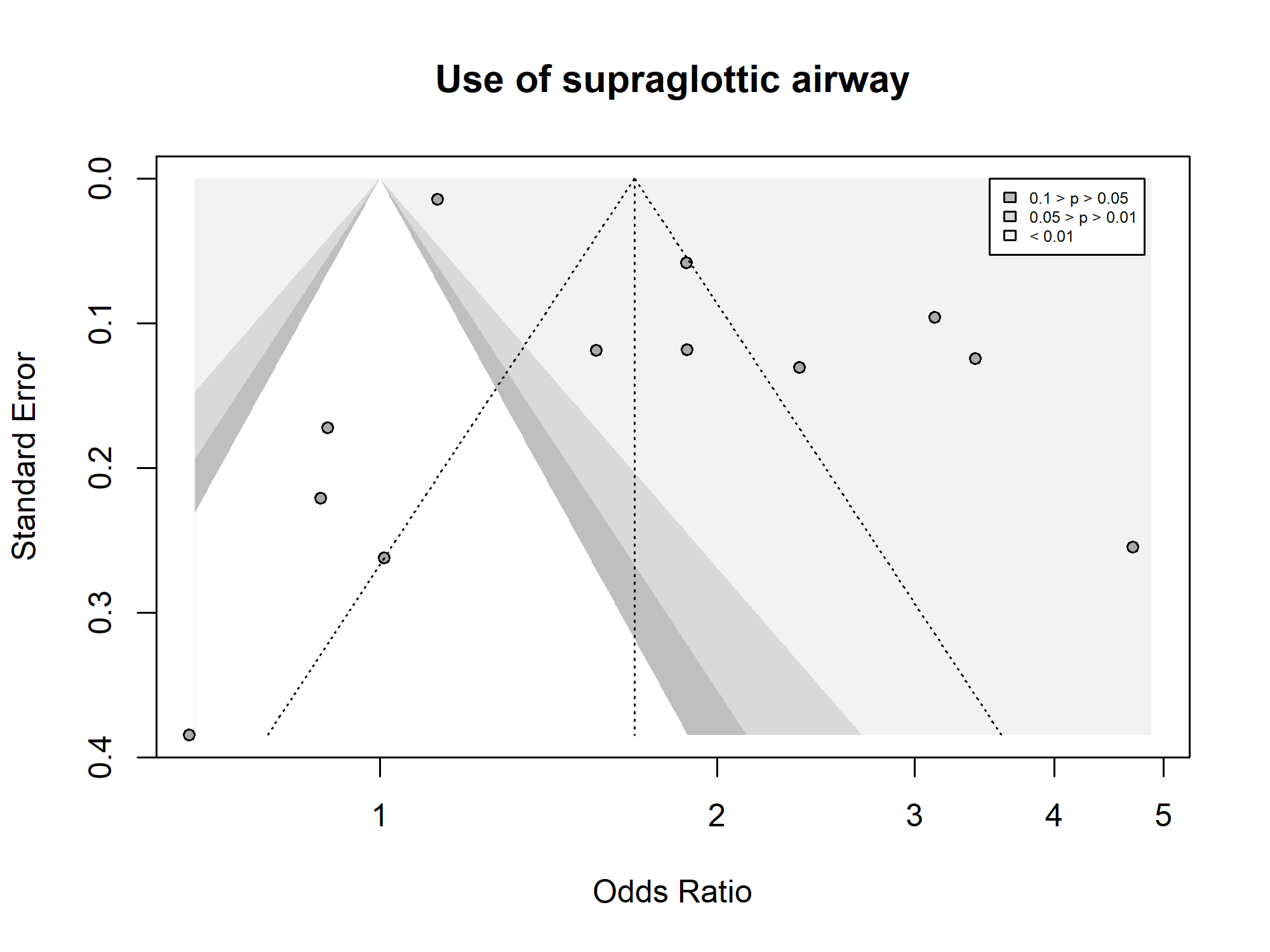


(m) Supraglottic airway device


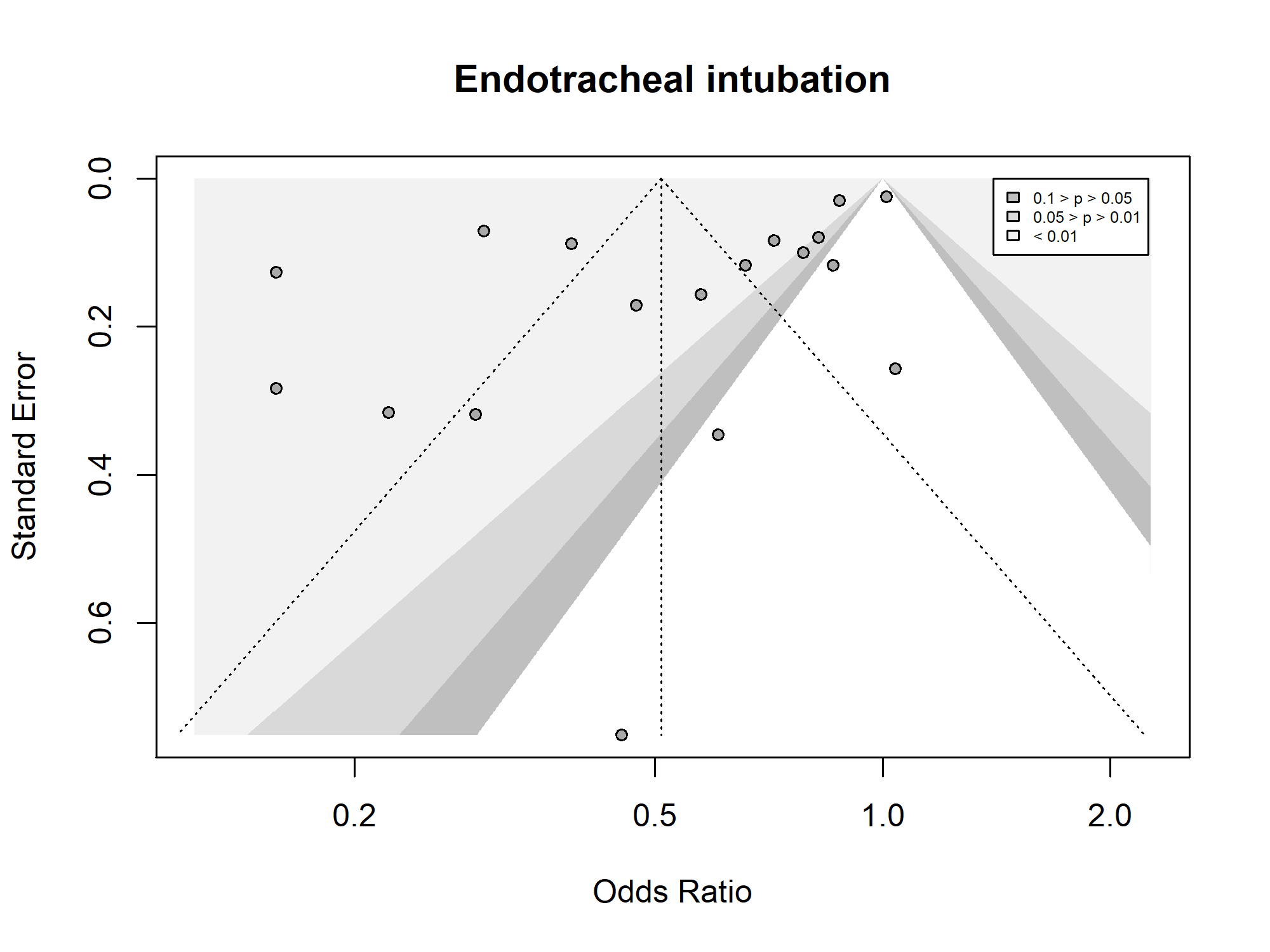


(n) Endotracheal intubation


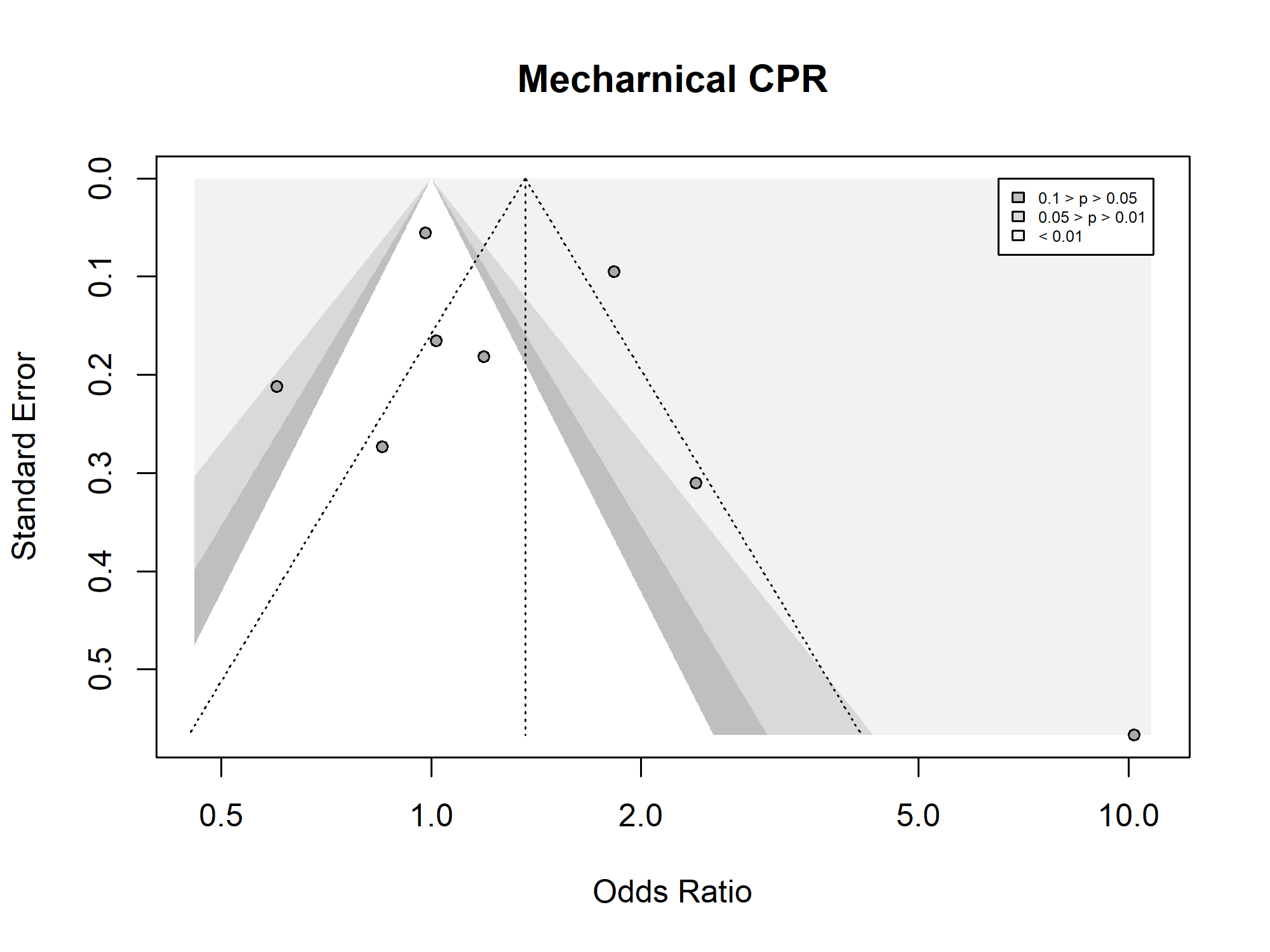


(o) Mechanical CPR


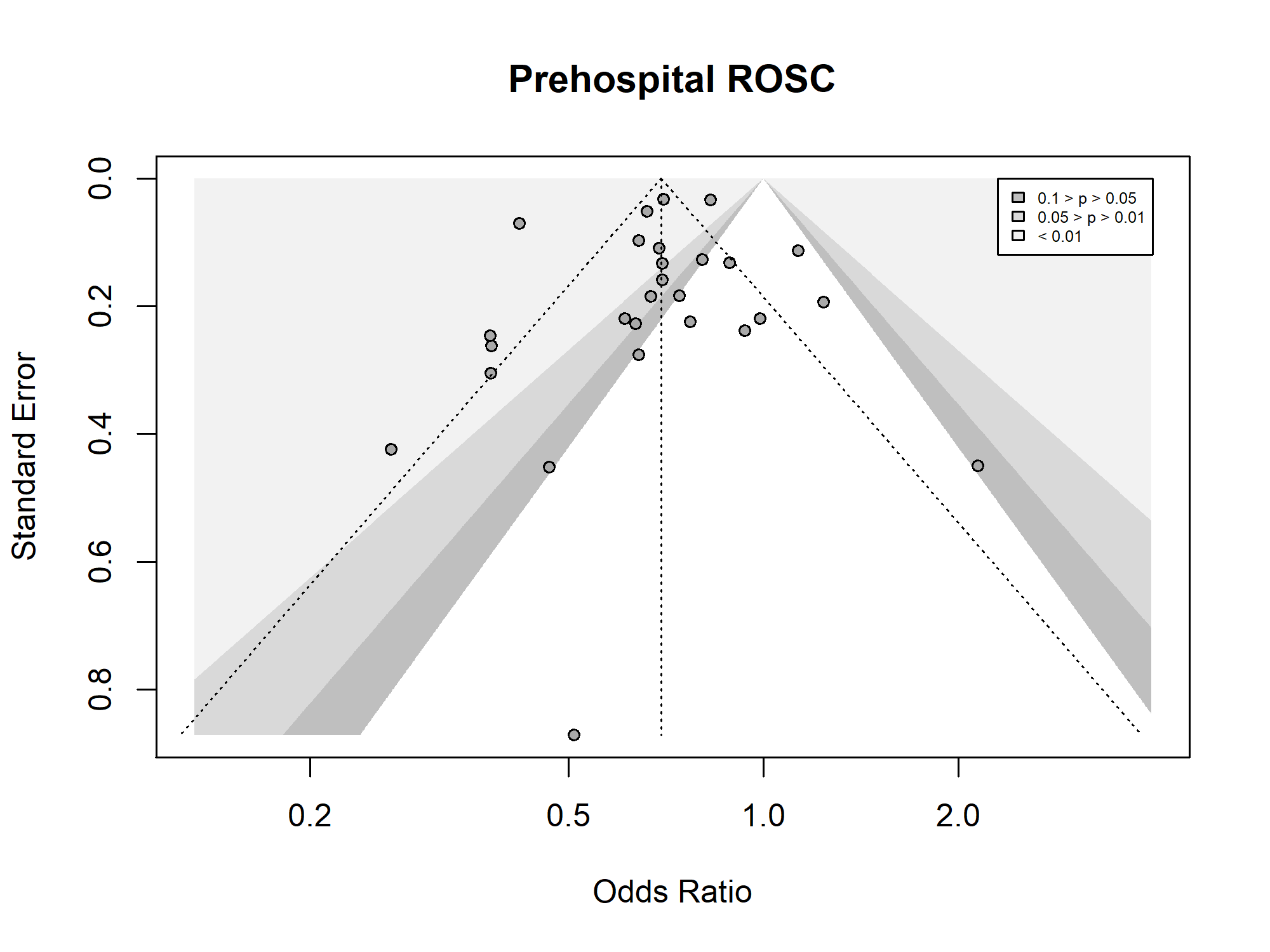


(p) Prehospital return of spontaneous circulation


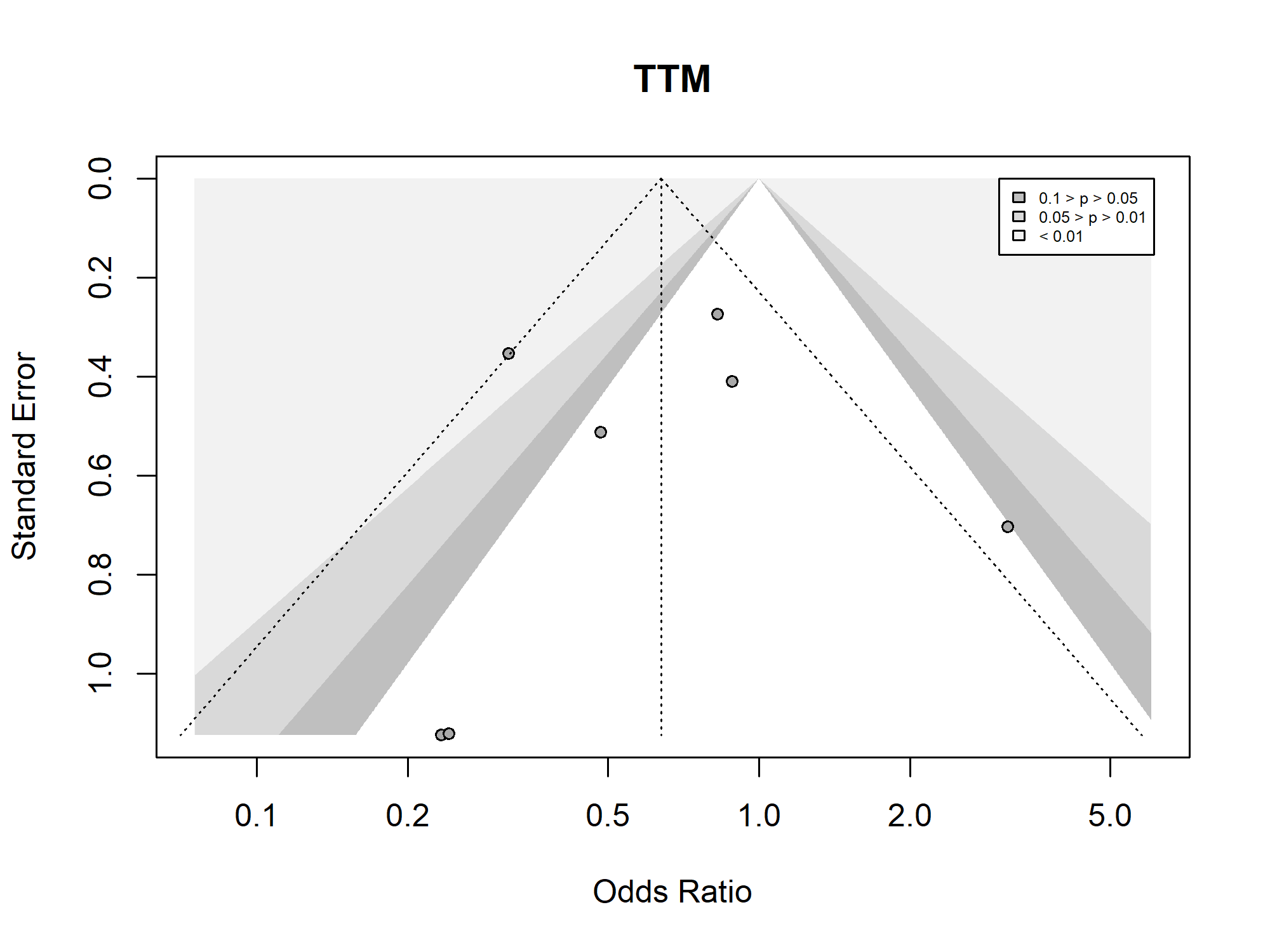


(q) Target temperature management
